# Fusion of Bipolar Tetraether Lipid Membranes Without Enhanced Leakage of Small Molecules

**DOI:** 10.1038/s41598-019-55494-z

**Published:** 2019-12-18

**Authors:** Geoffray Leriche, Dillan Stengel, David Onofrei, Takaoki Koyanagi, Gregory P. Holland, Jerry Yang

**Affiliations:** 1 Department of Chemistry and Biochemistry, University of California, San Diego, La Jolla, California 92093-0358 USA; 2 Department of Chemistry and Biochemistry, San Diego State University, San Diego, California 92182-1030 USA

**Keywords:** Chemistry, Membrane structure and assembly

## Abstract

A major challenge in liposomal research is to minimize the leakage of encapsulated cargo from either uncontrolled passive permeability across the liposomal membrane or upon fusion with other membranes. We previously showed that liposomes made from pure Archaea-inspired bipolar tetraether lipids exhibit exceptionally low permeability of encapsulated small molecules due to their capability to form more tightly packed membranes compared to typical monopolar lipids. Here, we demonstrate that liposomes made of synthetic bipolar tetraether lipids can also undergo membrane fusion, which is commonly accompanied by content leakage of liposomes when using typical bilayer-forming lipids. Importantly, we demonstrate calcium-mediated fusion events between liposome made of glycerolmonoalkyl glycerol tetraether lipids with phosphatidic acid headgroups (**GMGTPA**) occur without liposome content release, which contrasts with liposomes made of bilayer-forming **EggPA** lipids that displayed ~80% of content release under the same fusogenic conditions. NMR spectroscopy studies of a deuterated analog of **GMGTPA** lipids reveal the presence of multiple rigid and dynamic conformations, which provide evidence for the possibility of these lipids to form intermediate states typically associated with membrane fusion events. The results support that biomimetic GMGT lipids possess several attractive properties (e.g., low permeability and non-leaky fusion capability) for further development in liposome-based technologies.

## Introduction

Membrane fusion is a key event in many cellular processes in all three domains of life (Bacteria, Eukarya and Archaea). Exocytosis, fertilization, hormone secretion, neuronal signaling and viral infection of host cells are a few of the many biological processes found in living organisms that rely on some form of membrane fusion^1^. The process of membrane fusion varies widely in different systems, but the same basic steps exist for all mechanisms and starts with an aggregation phase to bring two membranes in close contact. Next, fusion of the outer leaflets of each membrane is thought to lead to the formation of a hemifused, “stalk-like” intermediate^2^. Finally, reorganization of the inner lipid leaflet results in pore opening and mixing of inner aqueous contents to complete the fusion process^3^. Studies have led to this stalk-hemifusion-pore hypothesis for the mechanism of membrane fusion of bacterial and eukaryotic lipid membranes^4,5^. However, it is not clear whether the same mechanistic pathway for fusion is accessible to membranes made of membrane-spanning bipolar lipids commonly found in Archaea.

Archaea have evolved mechanically and chemically robust lipid membranes that are thought to help with survival in extreme environments (e.g., low pH, high temperature and osmotic pressure)^6^. Membranes made of Archaeal lipids are known to exhibit high stability and low solute permeability thanks to unique ether-containing lipids with isoprene side chains^7^. The plasma membranes of Archaea are generally mixtures of bipolar tetraether lipids and monopolar diether lipids^8^. It has been shown that the fraction of bipolar tetraether lipids in Archaeal membranes increases with higher temperatures and varies among species (~90–95% in thermoacidophiles, 0–50% in methanogens, and virtually absent in halophiles)^9^. Archaeal membranes become more stable and less permeable to ions or solute as the fraction of tetraether lipids increases^10,11^. Membrane mixtures of extracted Archaeal lipids are generally stable against aggregation and are non-fusogenic unless exposed to the combination of acidic pH, high temperature, calcium ions and glycosidase^12,13^. Although the molecular mechanism for fusion of Archaeal lipid membranes is unknown, the fusogenic activity of Archaeal lipid membranes is often linked to the presence of bilayer-forming diether lipids, which can be found in most, if not all, extracted Archaeal lipid mixtures^9,14^. The fusogenic properties of membranes made of pure bipolar tetraether lipids has not been previously reported.

**Figure 1:**
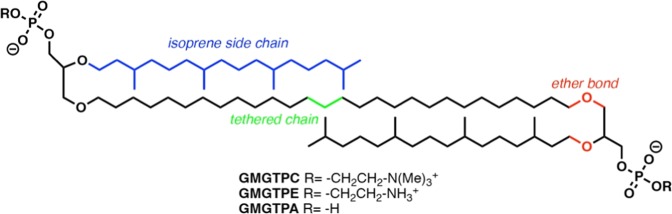
Chemical structure of bipolar tetraether lipids **GMGTPC**, **GMGTPE** and **GMGTPA**.

We and other groups have previously reported the synthesis of a series of glycerol monoalkyl glycerol tetraether lipids (GMGT) that incorporate some key structural features (e.g., ether linkages between a glycerol moiety in the headgroup and the lipid sidechains, tethering of lipid sidechains, and incorporation of rings within the tethered lipid sidechains) found in natural Archaeal lipids (Fig. 1)^15–19^. These synthetic lipids have shown great potential for biotechnological applications and especially in the field of liposomal drug/gene delivery^20–24^. Unlike extracted lipid mixtures, synthetic lipids offer the advantage of complete control of membrane lipid composition and, therefore, allow for systematic evaluation of the effects on membrane permeability of specific lipid structural features (e.g., tethering of lipid sidechains^18^, incorporation of rings in the tethered lipid sidechain^16^, inclusion of different polar headgroups^15^, and incorporation of different untethered sidechains^25^). Using the structure of natural Archaeal lipids as inspiration, we developed successive generations of tetraether lipids with reduced membrane permeability to small ions or organic solutes when compared to commercial bilayer-forming lipids^25^. While membranes comprised of tetraether lipids show great stability in typical bilayer membrane disrupting conditions, the stability of these biomimetic membranes has never been reported under fusogenic conditions.

Herein, we explore the calcium-induced fusion of liposomal membranes made of pure bipolar tetraether lipids with phosphatidic acid (PA) headgroups (**GMGTPA**) (Fig. 1). We also synthesized two new tetraether lipids incorporating fluorescent dyes to conduct lipid-mixing assays based on 7-nitro-2,1,3-benzoxadiazole-4-yl (NBD) and lissamine rhodamine B (Rho) fluorescence resonance energy transfer (FRET), and we use content mixing experiments based on 8-aminonaphthalene-1,3,6-trisulfonic acid (ANTS) and p-xylene-bis-pyridinium bromide (DPX) energy transfer to evaluate membrane leakage under typical membrane fusion conditions. NMR spectroscopy studies of a deuterated analog of **GMGTPA** lipids provide molecular insight on conformational constitution of pure bipolar PA lipids. The results support that biomimetic bipolar tetraether lipids have advantageous properties that could be useful for making robust liposomes with low leakage under a variety of environmental conditions.

## Results and Discussion

Liposomal membrane fusion is generally induced by fusogenic agents (or fusogens) that can either be large macromolecules such as proteins/peptides or small molecules or ions^3^. Fusogens are typically required to overcome the energetic barrier for propagation through the intermediate states along the membrane fusion pathway. Liposomal aggregation, which is a prerequisite for fusion, requires removal of the aqueous environment associated with the polar lipid headgroups and is expected to be one of the most energetically demanding steps along the fusion pathway^3^. Compared with other phospholipids, liposomes formed from lipids with phosphatidylcholine (PE) headgroups contribute to membrane fusion because of the low hydration of the PE headgroup compared to other common headgroups. However, preliminary studies showed that bipolar tetraether lipids with PE headgroups (**GMGTPE**, Fig. 1) do not induce liposomal membrane fusion or aggregation even when PEG^26^ or ethanol^27^ were used to enhance water removal at the surface of the membrane. We, therefore, directed our attention to bipolar tetraether lipids with PA headgroups (**GMGTPA**, Fig. 1). Liposomes prepared from PA lipids are generally less prone to aggregation than other phospholipids due to the electrostatic repulsion between the anionic lipid headgroups. However, this electrostatic repulsion can be diminished in the presence of divalent ions that can bind to the PA headgroups. Calcium ions (Ca^2+^), for instance, are powerful fusogen agents for membranes comprised of lipids with PA headgroups, as they are thought to shield the negative charge on PA and displace water on the surface of the membrane^28^.

### Design and synthesis of fluorescently-labelled lipids

**Figure 2:**
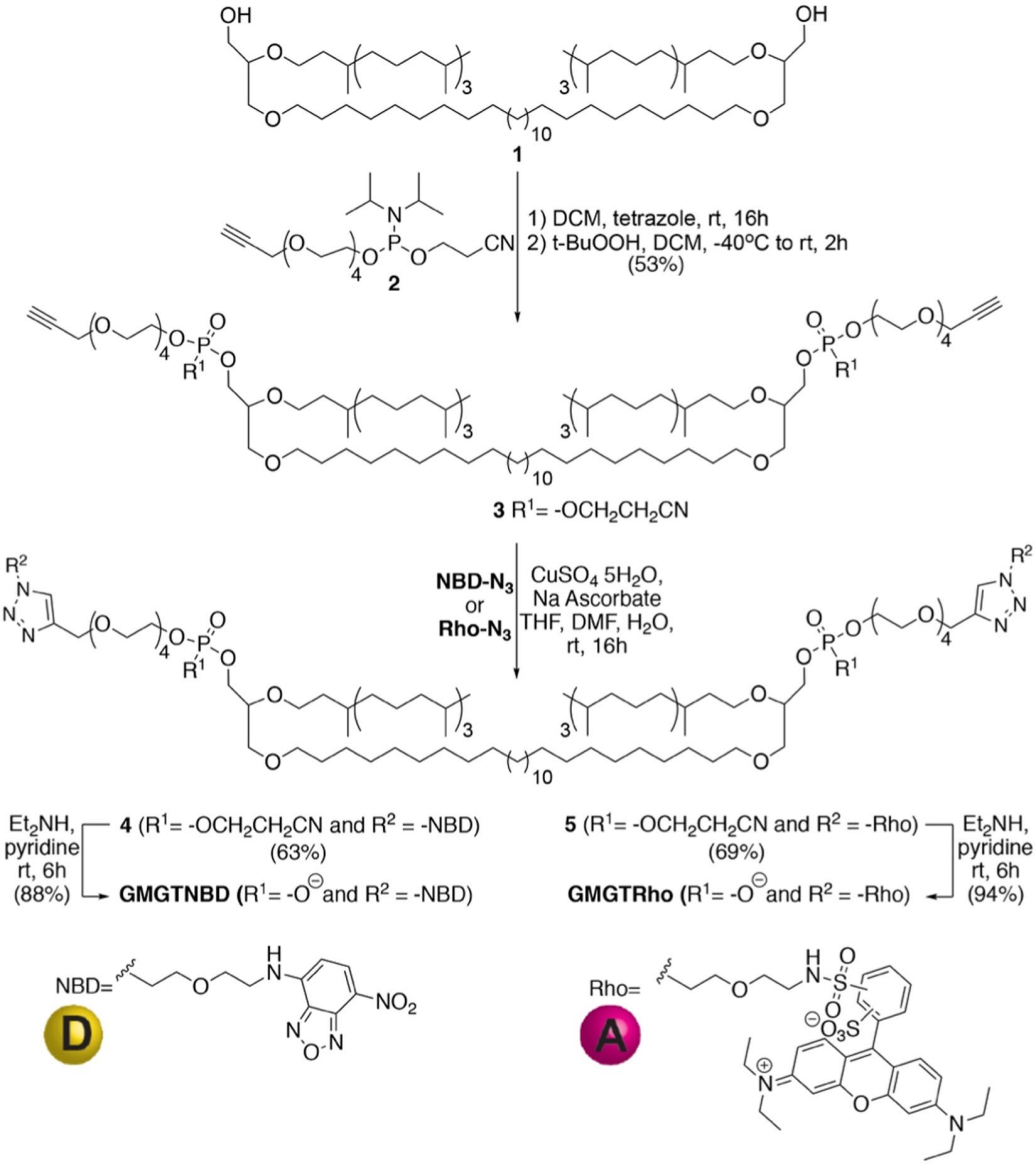
Synthesis of fluorescently-labelled lipids **GMGTNBD** and **GMGTRho**.

Fusion of liposomes is generally monitored by measuring liposome size distribution and using fluorescence spectroscopy assays in order to discriminate fusion from aggregation^29^. Fusion is typically confirmed when both the mixing of membrane lipids and the mixing of the aqueous contents of the fused liposomes is observed. The lipid mixing assay developed by Struck *et al*. is based on an NBD–rhodamine Förster resonance energy transfer (FRET) and requires two fluorescent lipids labelled with either Rho or NBD^29^. To prevent lipid exchange and to maintain membranes containing only pure bipolar tetraether lipids, we used tetraether lipids with fluorophores at both polar ends in the design of a lipid mixing assay. We synthesized the GMGT tetraether lipid core **1** in 9 synthetic steps as described previously^22^, which comprised a 28‐carbon aliphatic chain and two untethered phytanyl groups attached to a glycerol backbone through ether linkages (Fig. 2). Then, we used phosphoramidite chemistry to bis-functionalize diol **1** with alkyne phosphate headgroups (Fig. 2). Mono-propargylated tetraethylene glycol was generated and reacted with 2-cyanoethyl N,N,N′,N′-tetraisopropyl-phosphorodiamidite to form **2** in good yield (see the supporting information for experimental details of the synthesis). Phosphoramidite **2** was then reacted with lipid core **1** before the resulting intermediate was oxidized into the corresponding phosphate‐triester **3**. Azide analogs of NBD (**NBD-N**_**3**_) and Rho (**Rho-N**_**3**_) were prepared and reacted with alkyne **3** in the presence of copper(II) sulfate and sodium ascorbate to form protected lipids **4** and **5** in 63% and 69% yield, respectively. Finally, removal of 2-cyanoethyl groups from the phosphate backbone under basic conditions led to the formation of **GMGTNBD** and **GMGTRho** lipids (in 88% and 94% yield, respectively).

### Calcium-Induced lipid mixing of liposomes

**Figure 3:**
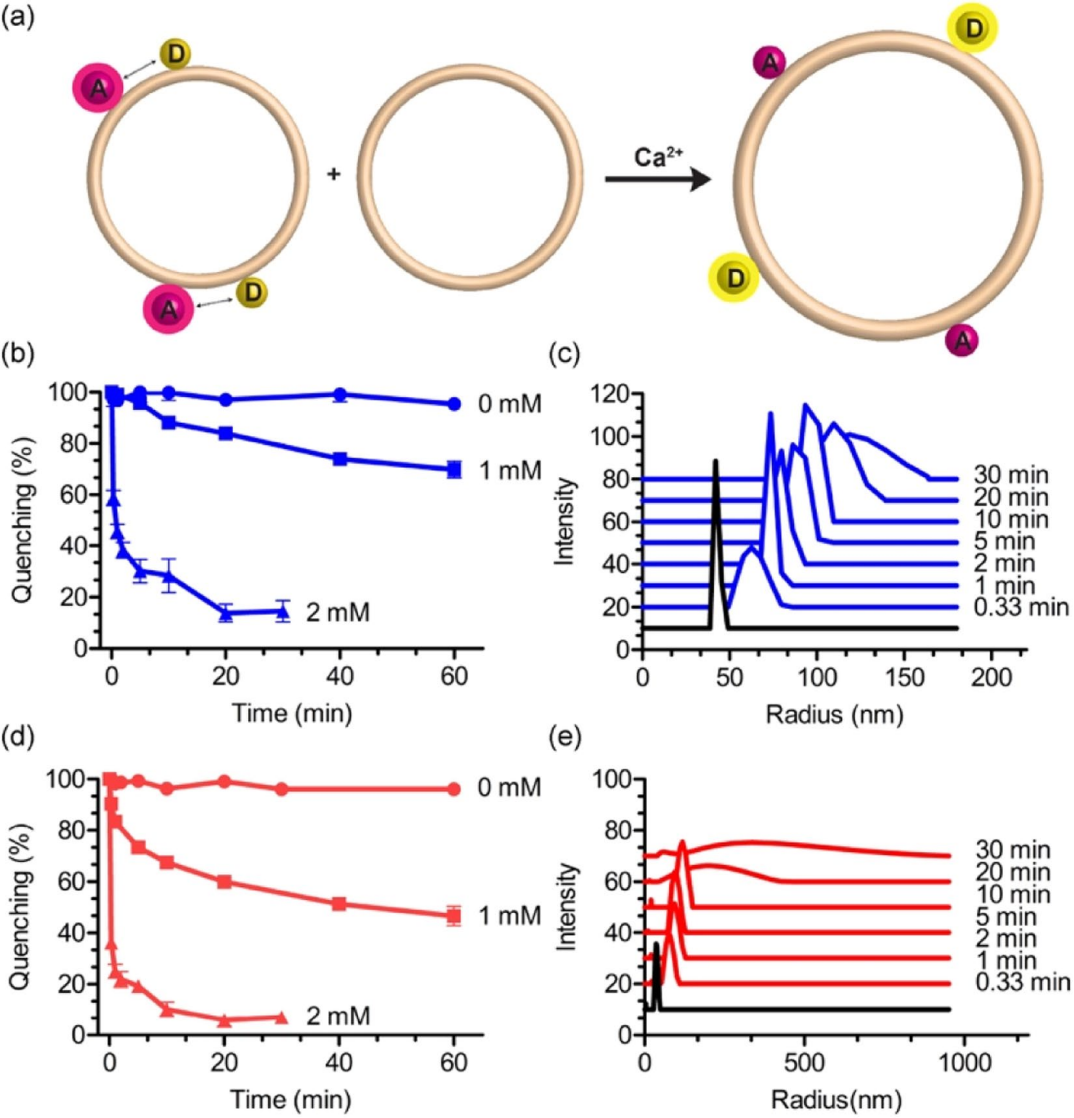
Lipid mixing experiments. (**a**) Scheme of the lipid mixing assay based on fluorescence resonance energy transfer (FRET). The average spatial separation of the donor (D) and acceptor (A) fluorescent probes increases upon fusion of labeled membranes with unlabeled membranes, resulting in decreased efficiency of proximity-dependent FRET. Liposomes consisted of **GMGTPA** (**b**) and **EggPA** (**d**), containing the donor (NBD) and acceptor (Rho) dye, were mixed with non-labeled liposomes (ratio 1:10), and the increase in donor fluorescence emission (at 530 nm) was monitored with and without added Ca^2+^ (1 and 2 mM). (**c,e**) Size distribution of liposomes in the presence of 2 mM Ca^2+^ concentration. The hydrodynamic radius of the liposomes made of **GMGTPA** (**c**) and **EggPA** (**e**) were measured by dynamic light scattering and compared to liposome without Ca^2+^ (black traces). All experiments were carried at room temperature in TES buffer (10 mM, 2 mM Histidine, 0.1 mM EDTA, NaCl 100 mM, pH 7.4) with 110 μM of total lipid concentration. For each time point, an aliquot was taken and quenched with EDTA (100 mM) prior to measurement. Error bars represent s.d. (N = 3).

In the lipid mixing experiment, liposomes made of PA lipids labelled with a combination of 0.05 mol% of NBD-lipid (FRET donor) and 0.05 mol% of Rho-lipid (FRET acceptor) are mixed with unlabelled liposomes at a 1:10 ratio (Fig. 3a)^30^. Fluorescence resonance energy transfer decreases when the average spatial separation of the fluorescent probes is increased upon mixing of labelled liposomes with unlabelled liposomes. We prepared two batches of liposomes from **GMGTPA** lipids (with and without **GMGTNBD** and **GMGTRho** lipids, 0.05 mol% each lipid) by hydration of thin films in TES buffer, followed by extrusion. This preparation afforded liposomes with an average hydrodynamic radius of about 45 nm, as determined by dynamic light scattering (DLS). Then, the liposomes were incubated with different concentration of calcium (1 and 2 mM of CaCl_2_) and NBD dequenching (emission at ~530 nm) was measured at different time points (Fig. 3b). Complete mixing of all the lipids was estimated by the addition, at the end of the experiment, of detergent (E8C12) that disrupts the liposomes and provides a final fluorescence signal that serves as an estimate of complete dequenching of NBD. The fusion/aggregation process was quenched by the addition of ethylenediaminetetraacetic acid (EDTA) and the average radius of the liposomes was measured by DLS for each time point (Fig. 3c). An identical protocol was followed to study the fusion of liposomes made of **EggPA** using commercially available NBD and Rho bilayer-forming lipids (Fig. 3d,e).

In the absence of calcium, neither liposomes made from pure **GMGTPA** nor **EggPA** showed decrease in FRET efficiency, indicating no spontaneous lipid exchange between fluorescently labelled and unlabelled liposomes (Fig. 3b,d). However, a rapid and concentration-dependent increase of NBD fluorescence (corresponding to decreased quenching as shown in Fig. 3b,d) was observed for both lipids systems in the presence of calcium. The extent of lipid mixing between **EggPA**-containing liposomes increased more rapidly compared to **GMGTPA**-containing liposomes (e.g., ~47% and ~70% of NBD-dequenching for **GMGTPA** and **EggPA**, respectively, after 1 hour in the presence of 1 mM CaCl_2_). Increase in NBD fluorescence was accompanied by a large change in liposome size distribution, supporting calcium-mediated aggregation (Fig. 3c,e). Similar to the results observed from the decrease of FRET after mixing (Fig. 3b,d), the change in size distribution for liposomes made from **EggPA** appeared qualitatively larger than for liposomes made from **GMGTPA**. Thus, these results demonstrate that membranes made from tetraether lipids can undergo lipid mixing concomitantly with aggregation. However, lipid mixing alone is not necessarily evidence of fusion, since the FRET-based assay cannot distinguish between stages of hemifusion and full fusion^31^. We, therefore, next investigated the capability of liposomes to mix content upon addition of calcium.

### Calcium-induced content mixing and leakage of liposomes

**Figure 4:**
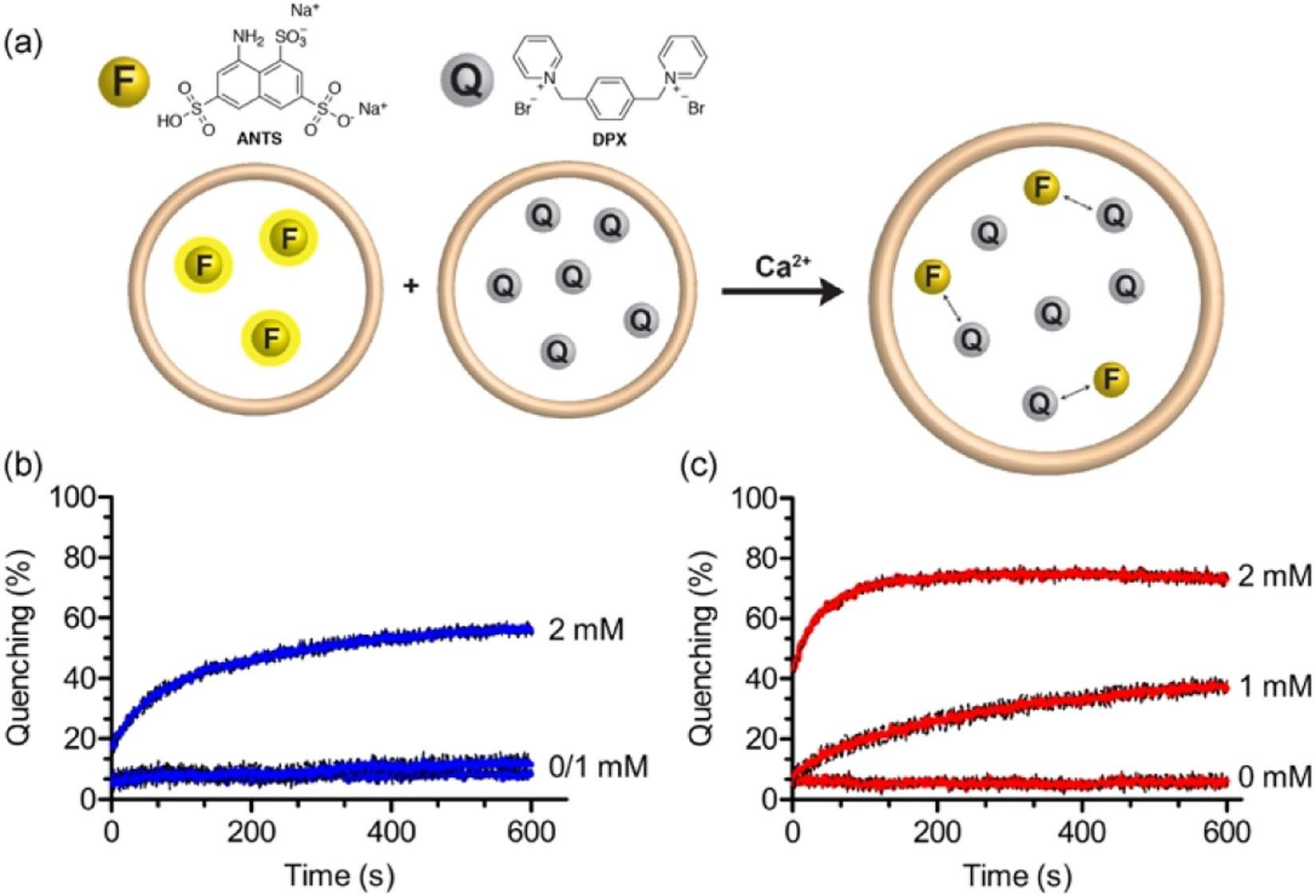
Content mixing experiments. (**a**) Scheme of the content mixing assay based on the collisional fluorescence quenching of the polyanionic fluorophore ANTS (F) and the cationic quencher DPX (Q). Liposomes consisted of **GMGTPA** (**b**) and **EggPA** (**c**) containing ANTS (40 mM) were mixed with liposomes containing DPX (90 mM) at a 1:1 ratio, and the quenching of ANTS fluorescence emission (at 530 nm) from liposomal content mixing was monitored with and without Ca^2+^ (1 and 2 mM). All experiments were carried at room temperature in TES buffer (10 mM, 2 mM Histidine, 0.1 mM EDTA, NaCl 100 mM, pH 7.4) with 50 μM of total lipid concentration. Error bars represent s.d. (N = 3).

A commonly used assay to monitor content mixing of liposomes upon fusion is based on the collisional quenching of the polyanionic fluorophore ANTS by the cationic quencher DPX (Fig. 4a)^32^. Briefly, when two populations of liposomes loaded with either ANTS or DPX are mixed, the extent of fusion is directly monitored by measuring quenching of ANTS fluorescence. In this assay, any leakage of content from liposomes into the surrounding solution does not cause fluorescence quenching due to the accompanying high dilution of ANTS and DPX.

**Figure 5:**
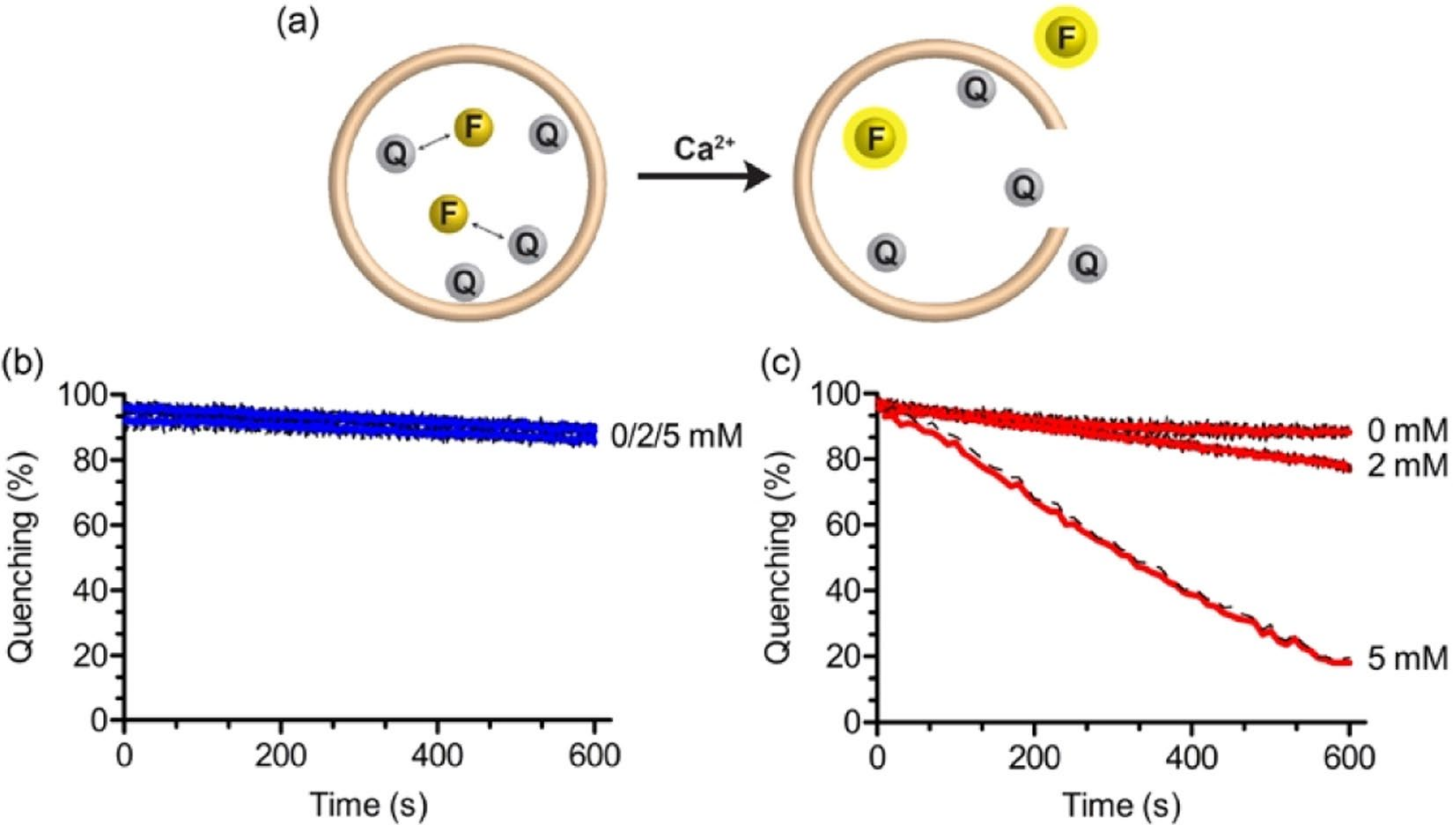
Content leakage experiments. (**a**) Scheme of the content release assay based on the collisional quenching of the polyanionic fluorophore ANTS (F) by the cationic quencher DPX (Q). Liposomes consisted of **GMGTPA** (**b**) or **Egg-PA** (**c**) containing both ANTS (20 mM) and DPX (45 mM) were mixed and the decrease of quenching of ANTS fluorescence (at 530 nm) due to content leakage was monitored with or without added Ca^2+^ (1 and 2 mM). All experiments were carried at room temperature in TES buffer (10 mM, 2 mM Histidine, 0.1 mM EDTA, NaCl 100 mM, pH 7.4) with 50 μM of total lipid concentration. Error bars represent s.d. (N = 3).

We prepared ANTS and DPX-loaded liposomes by passive encapsulation using thin-film hydration and then purified them through a sephadex G25 resin to remove unencapsulated dyes. A third preparation of liposomes co-encapsulating ANTS and DPX was prepared and used to provide a final fluorescence signal that represents what we would expect if we observed complete content mixing after liposomal fusion. ANTS and DPX-liposomes were mixed (1:1 ratio) and fluorescence emission of ANTS was measured at 530 nm while size distribution of liposomes was monitored by DLS (see Fig. S1 in the supporting information). In the presence of calcium ions, rapid quenching of ANTS fluorescence was observed for both liposomal systems comprised of either **GMGTPA** or **EggPA** (Fig. 4b,c). These results, thus, confirms the ability of liposomal membranes of bipolar tetraether lipids to mix intraliposomal content, and, therefore, fuse upon addition of calcium. We attribute the small differences in the observed kinetics of content mixing and the sensitivity to calcium ions for both lipid systems, compared to the lipid mixing assay, to differences in liposome concentration required for each assay (110 μM and 50 μM total lipid concentration for the lipid mixing assay and the content mixing assay, respectively). Nevertheless, membranes made from **GMGTPA** lipids exhibited slow kinetics for lipid and content mixing when compared to bilayer forming **EggPA** lipids. For content mixing, both liposomal systems showed an initial rapid decrease (i.e., increased quenching) in fluorescence followed by a plateau of fluorescence intensity, possibly reflecting either slower fusion kinetic rates after a burst caused by initial aggregation and/or dye leakage upon aggregation/fusion (Fig. 4b,c). In order to examine fusion-induced leakage of liposomal content, we next studied membrane leakage using liposomes co-encapsulating ANTS and DPX. In this assay, ANTS fluorescence is expected to increase upon liposomal leakage because quenching by DPX will be diminished when either molecule leaks into the surrounding medium (Fig. 5a).

Incubation of liposomes co-encapsulating ANTS and DPX in buffer only (i.e., no added calcium) showed that both lipids were able to retain ANTS and DPX without leakage over the course of 10 minutes (Fig. 5b,c). As expected, liposomes made from **EggPA** displayed strong leakage upon fusion in the presence of added calcium (20% and 80% leakage of content after 10 minutes in the presence of 2 and 5 mM CaCl_2_, respectively). However, we did not observe any leakage of content with liposomes made from **GMGTPA** in presence of calcium (i.e., under experimental conditions where we observe aggregation and fusion of liposomes). Whereas calcium-induced leakage due to fusion is well-known with bilayers made of lipids with PA headgroups^28^, the results demonstrate that liposomes made from **GMGTPA** are able to undergo non-leaky fusion events. These surprising results prompted us to examine whether different conformations of **GMGTPA** lipids in membranes could provide additional molecular insight on the mechanism for the observed fusion events.

### Design and synthesis of deuterated lipids for lipid conformation studies

**Figure 6:**
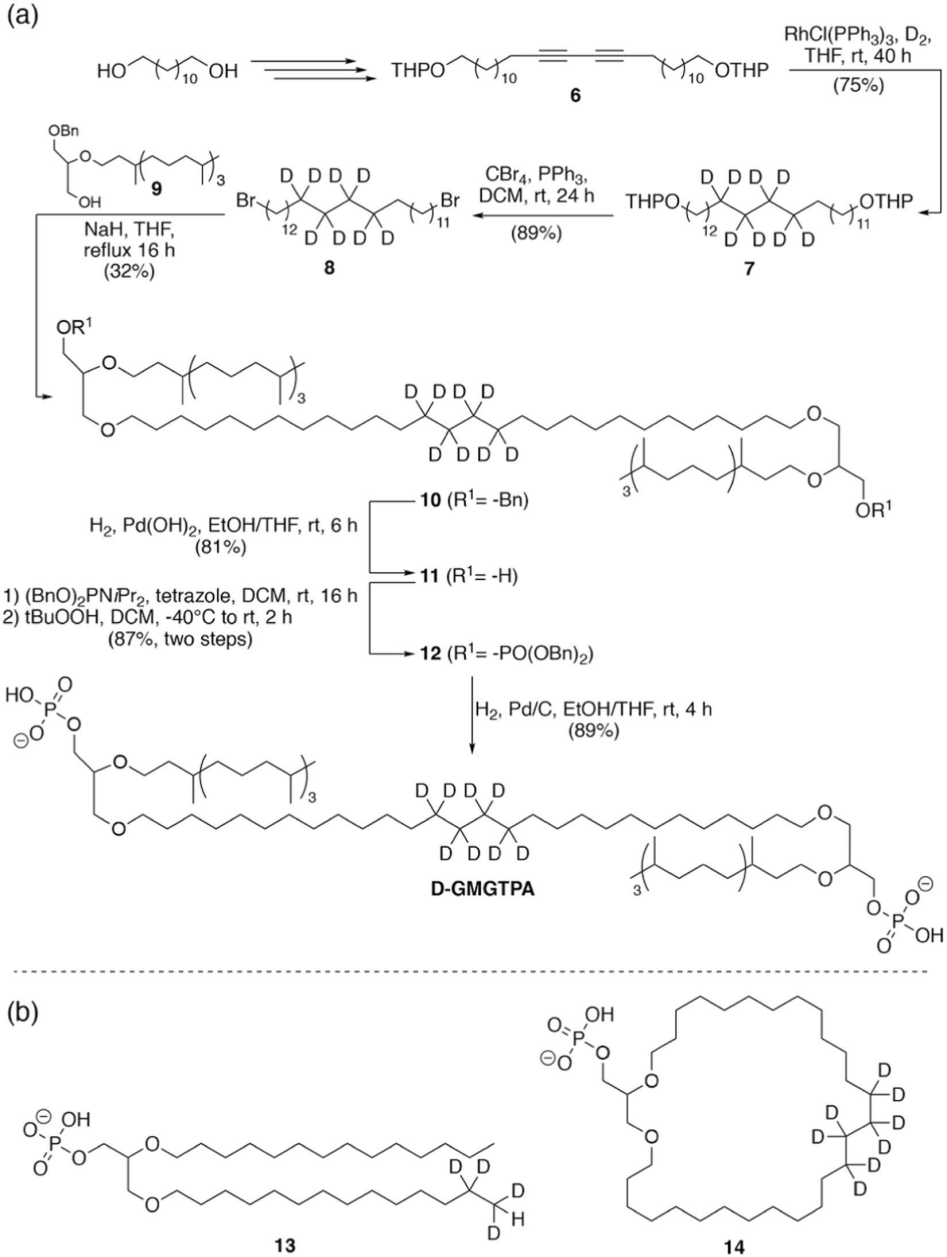
Synthesis and structure of deuterated PA lipids. (**a**) Synthesis of **D-GMGTPA**. (**b**) chemical structures of deuterated bilayer forming lipids **13** and **14** (spanning model and looping model, respectively).

Previous studies using deuterated lipids and solid-state ^2^H NMR spectroscopy suggested that hemicyclic tetraether lipids have different lipid orders when organized as membranes^33,34^. A less ordered conformer (estimated by the splitting patterns of the de-Paked spectra in the ^2^H NMR spectrum) has previously been assigned to a looping conformation (i.e., U-shaped conformation with both polar headgroups on the same leaflet of the membrane), whereas a highly ordered splitting pattern has been assigned to a fully membrane spanning conformation. Unfortunately, lipids used in these previous studies do not possess isoprene side chains or PA headgroups as in **GMGTPA**, making it difficult to assume that **GMGTPA** lipids adopt essentially the same distribution or type of conformations as other hemicyclic tetraether lipids reported in the literature. Therefore, we synthesized a new set of deuterated lipids in order to estimate the order and dynamics of lipid membranes made from **GMGTPA**, including an octa-deuterated analogue of **GMGTPA** labelled in the middle of the tethered chain (**D-GMGTPA**, Fig. 6a), and two model bilayer-forming lipids **13** and **14**, that could potentially mimic spanning or looping conformations of **D-GMGTPA**, respectively (Fig. 6b)^33^.

A general outline of the synthesis of the deuterated lipid **D-GMGTPA** is shown in Fig. 6a. Dialkyne **6** was prepared from dodecandiol by dimerization of an acetylenic derivative under Glaser coupling conditions, and reduction of the formed 1,3-diyne with deuterium in the presence of Wilkinson’s catalyst led to the formation of **7**. Bromination of **7** with carbon tetrabromide as a bromine source afforded deuterium labelled 1,28-dibromooctacosane **8** in good yield. This dibromide was reacted with glycerol scaffold **9**, incorporating a phytanyl side chain, in the presence of sodium hydride to form the tetraether core **10**. After debenzylation of **10** under reductive conditions, the lipid with protected phosphate headgroups **12** was generated by reaction of diol **11** with dibenzyl N,N-diisopropylphosphoramidite and subsequent oxidation of the resulting intermediate with tert-butyl peroxide. Finally, palladium-catalysed hydrogenation of **12** gave **D-GMGTPA** lipid in 89% yield. The two model diether bilayer-forming lipid cores, for spanning and looping conformations, were synthesized following the strategy used by Cuccia *et al*.^33^, and the PA headgroups were introduced in an analogous manner as shown in Fig. 6a (see Figs. S2 and S3 and additional details in the supporting information for the synthesis of lipids **13** and **14**).

### ^2^H-NMR spectroscopy studies of D-GMGTPA

^2^H NMR spectroscopy provides dynamic and organizational data about individual C–^2^H bonds and is used to characterize the motions associated with a number of macromolecules, including lipids^35^. The coupling of the deuteron with the local C–^2^H electric field gradient produces a splitting pattern whose peak-to-peak splitting (Δv) is a direct measure of the dynamics of the nuclei pair, where a larger Δv corresponds to a more rigid C–^2^H bond vector, and a smaller Δv corresponds to a more dynamic one. For oriented or powder-type systems like those found in lipid vesicles, the segmental order parameter (S_CD_) quantifies the average structure of the C–^2^H bond vectors along the lipid chain with respect to the bilayer normal, where a higher S_CD_ corresponds to a C–^2^H vector that is on average more perpendicular with the bilayer normal.

**Figure 7:**
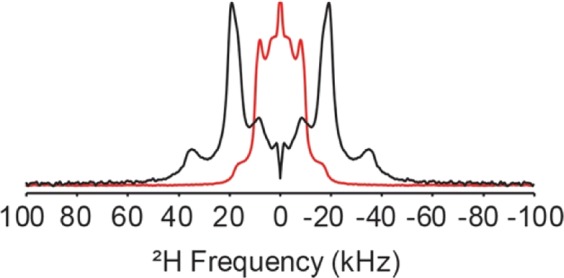
^2^H NMR powder pattern (red) and de-Paked (black) spectrum for bipolar tetraether lipid **D-GMGTPA** at 30 °C.

Figure 7 shows the static ^2^H NMR spectra of the bipolar tetraether lipid **D-GMGTPA**. Four sets of splitting patterns are observed for the de-Paked spectra with an approximate splitting, Δv, of 18, 34, 40, and 70 kHz corresponding to order parameters, S_CD_, of 0.07, 0.13, 0.15 and 0.27, respectively. When the bipolar tetraether ^2^H splitting patterns are compared to the spanning and looping controls (lipids **13** and **14**, respectively), we find that the splitting for the bipolar tetraether lipid is generally smaller compared to the looping model or the penultimate CD_2_ group of the spanning model, which serves as a reference for typical dynamics observed for a centrally located CD_2_ group in a diether bilayer (see Fig. S4 in the supporting information for powder-type and de-Paked spectra of all three lipids). In the bipolar tetraether lipid, the untethered chain is comprised of isoprene units, which could impart increased chain dynamics compared to their acyl counterparts. Previous work investigating the ^2^H splitting patterns for deuterated tethered lipids have proposed that splitting patterns less than or equal to ~40 kHz generally correspond to looping conformations and splittings near 70 kHz correspond to spanning conformers^33,35,36^. Applying these conformational interpretations of ^2^H splitting patterns to **D-GMGTPA**, we can infer that a more rigid, fully membrane spanning conformation only constitutes 16% of the lipid membrane, with the remaining 84% of lipids in more dynamic environments (which could include looping conformations). ^31^P NMR data collected on these lipids also agree that **D-GMGTPA** displays increased headgroup dynamics in the form of narrower chemical shift anisotropy (CSA) powder patterns when compared to either the spanning or looping models **13** and **14** (Fig. S5).

The presence of at least some **GMGTPA** lipids in looping conformations could allow the formation of two distinct leaflets of a bilayer, and could make it possible for the co-existence of monolayer and bilayer domains in the membrane. Similar to bilayer domains formed by diether lipids in natural Archaeal membranes, we propose that the dynamic bilayer domains formed by **GMGTPA** lipids in a looping-like conformation could contribute to the formation of a hemi-fused, “stalk-like” intermediate leading to the observed calcium-mediated fusion events.

## Conclusions

We have, thus, demonstrated that bipolar tetraether lipids with phosphatidic acid headgroups, **GMGTPA**, have fusogenic capabilities in presence of calcium ions. The kinetics of fusion appeared to be slower for the bipolar tetraether lipid when compared with a commercial bilayer-forming lipid, **EggPA**. While the singly tethered lipid reported here is distinct from natural lipid mixtures found in Archaea, the observation that liposomes comprising pure bipolar **GMGTPA** can undergo membrane fusion contrasts previous literature reports that suggest the presence of monopolar lipids are required for fusion of Archaeal lipid membranes^9,14^. Additionally, liposomes made from **EggPA** lipids show significant leakage of encapsulated content upon fusion, whereas liposomes formed from **GMGTPA** displayed complete retention of encapsulated material under the same fusogenic conditions. These findings suggest that tetraether lipids could be a valuable liposomal platform for, for instance, the delivery of encapsulated drugs to cells where membrane fusion-induced leakage poses a challenge. Solid state ^2^H NMR studies support that **GMGTPA** lipids may adopt both spanning or looping conformations, providing a plausible mechanistic explanation for the fusion of membranes made from pure bipolar tetraether lipids through the formation of a hemifused intermediate. Additional studies are underway aimed at elucidating the detailed structure of **GMGTPA** and other relevant lipids in order to help explain their remarkable integrity with respect to permeability and leakage under a wide range of environmental conditions.

## Methods

### Lipid synthesis and NMR

See supporting information.

### Lipid mixing assay

10 mg/mL liposome solution was prepared by first dissolving 5 mg of lipids (Egg-PA mixed with 0.05 mol% of each PE-NBD and PE-Lissamine or **GMGTPA** mixed with 0.05 mol% of each **GMGTNBD** and **GMGTRho**) into a 5 mL round bottom flask in a DCM/MeOH (7/3) solution. A thin lipid film was achieved by evaporating the solvent using a rotary evaporator (BUCHI RE111) then dried further over a high-vacuum pump (Welch 1402) for 4 hrs. The lipid film was then hydrated, using a solution comprised of 2 mM TES, 2 mM Histidine, 100 mM NaCI and 0.1 mM EDTA, pH 7.4 (*Buffer 1*), by vortexing the solution for 30 seconds followed by sonication in a water bath sonicator (Branson 2510) for 30 mins. After sonication, the liposome suspension underwent 5 freeze thaw cycles that consisted of 2 mins at −78 °C followed by 2 mins at 50 °C. The lipid solution was then extruded (Avanti mini-extruder) through 200 nm and 100 nm polycarbonate membrane (25 times each) followed by another extrusion with a 50 nm polycarbonate membrane 51 times. The exact same procedure was used to prepare non-labelled liposomes using either **GMGTPA** or **Egg-PA**. All lipid solutions were then stored at 4 °C. Phospholipid concentrations were determined using a phosphate assay as described by Barlett and modified by Barenholz^37^, and liposome radius were measured by DLS.

In a typical assay with a total lipid concentration of 110 μM, NBD/Rho-labelled liposomes (120 μL at 100 μM) and non-labeled liposomes (120 μL at 1 mM) were mixed in *buffer 1* (960 μL) and incubated at room temperature in an Eppendorf tube. For each time point, an aliquot (135 μL) was taken and quenched with EDTA (15 μL, 100 mM in *buffer 1*) prior to fluorescence measurement. At the end of the experiment, a final aliquot (135 μL) was mixed with 15 μL of *buffer 1* containing 100 mM EDTA and 10% w/w E8C12.

The NBD emission was monitored at 530 nm with the excitation wavelength set at 460 nm. The fluorescence scale was calibrated such that:

- 100% NBD quenching value (0% lipid mixing) corresponded to the initial fluorescence of the labeled vesicles in absence of calcium.
- 0% NBD quenching value (0% lipid mixing) corresponded to the fluorescence of the labeled vesicles in presence addition of detergent (E8C12).

All measurements were performed at room temperature in a 96 well plate (100 μL per well, see section 2) and done in triplicate. For each time point, the aliquot quenched with EDTA was further diluted in *buffer 1* (4-times) and the average radius of the liposomes was measured by DLS.

### Content mixing assay

Three liposomes suspensions (ANTS, DPX and ANTS-DPX) were prepared as described by Düzgüneş *et al*.^30^ In three different 5 mL round bottom flasks, 5 mg of lipids (**EggPA** or **GMGTPA**) were first dissolved in a DCM/MeOH (7/3) solution. Thin lipid films were achieved by evaporating the solvent using a rotary evaporator (BUCHI RE111) then dried further over a high-vacuum pump (Welch 1402) for 4 hrs. The lipid films were then hydrated, using a solution comprised of either:

- 25 mM aminonaphthalene trisulfonic acid (ANTS; Life Technologies), 40 mM NaCl, 10 mM TES, pH 7.4 (*Buffer 2*)
- 90 mM p-xylene bis(pyridinium) bromide (DPX, Life Technologies), 10 mM TES, pH 7.4 (*Buffer 3*).
- A mixture of *Buffer 2* and *Buffer 3* (1:1).

After being vortexed for 30 seconds followed by sonication in a water bath sonicator (Branson 2510) for 30 mins, the different liposome suspensions underwent 5 freeze thaw cycles that consisted of 2 mins at −78 °C followed by 2 mins at 50 °C. The resulting suspensions were then extruded (Avanti mini-extruder) through 200 nm and 100 nm polycarbonate membrane (25 times each) followed by another extrusion with a 50 nm polycarbonate membrane 51 times. The lipid solutions were then stored at 4 °C. Before every experiment, liposomes were purified on sephadex G25 equilibrated with TES buffer (100 mM NaCl, 0.1 mM EDTA, 10 mM TES, pH 7.4, *Buffer 4*). Briefly 100 μL of liposome stock solution was diluted in 400 μL of *buffer 4*, and the resulting solution was purified through two successive sephadex G25. Phospholipid concentrations were determined using a phosphate assay^37^ and liposome radius were measured by DLS.

In a typical assay with a total lipid concentration of 50 μM, the fluorescence of ANTS liposomes (40 μL at 625 μM) and DPX liposomes (40 μL at 625 μM) in *buffer 4* (920 μL) is set to 100%. The fluorescence of ANTS–DPX liposomes (80 μL at 625 μM in 920 μL of *buffer 4*) is set to 0%. These liposomes represent the theoretical fusion product of all of the liposomes in the assay. Fusion was monitored as the decrease in fluorescence of ANTS.

$${\rm{F}}({\rm{t}})=100\times \frac{{{\rm{I}}}_{0}-{{\rm{I}}}_{t}}{{{\rm{I}}}_{0}-{{\rm{I}}}_{\infty }}$$

The extent of fusion, F(t), as a percentage of maximal fluorescence, is given by Equation (1), where the fluorescence intensity at time t is I_t_, and the fluorescence intensities of ANTS liposomes and ANTS–DPX liposomes are I_0_ and I_∞_, respectively. ANTS leakage upon fusion was assessed by monitoring the increase of ANTS fluorescence of ANTS–DPX liposomes in presence or absence of calcium ions. For experiments in presence of Ca^2+^ ions, CaCl_2_ was directly dissolved into *buffer 4* to generate 5 mM, 2 mM or 1 mM Ca^2+^ solutions.

For fluorescence measurements, the ANTS emission was monitored at 530 nm with the excitation wavelength set at 360 nm. All kinetic experiments were performed at room temperature in a 96 well plate (100 μL per well, see section 1) and done in triplicate. At the end of each kinetic experiment, the fusion and aggregation process were quenched by the addition of EDTA (10 μL, 100 mM in *Buffer 4*) and the average radius of the liposomes was measured by DLS.

### Solid state NMR experiments

#### Sample preparation

Lipids were dried under vacuum overnight prior to hydration. Then, ^2^H-depleted water was added to make a 50% w/w mixture. The samples were subjected to 5 freeze (−78 °C), thaw (50 °C) cycles and sonicated for 1 hour before recording the ^2^H NMR spectra. Sample tubes were closed with Teflon plugs.

#### ^2^H-NMR spectroscopy

Static ^2^H NMR experiments were performed with a 600 MHz Bruker Avance III HD NMR spectrometer equipped with a broad band 4 mm MAS probe. A COM-II quadrupolar echo sequence with a 4.27 μs 90° pulse width with composite pulses (90°_x_180°_−x_ 90°_x_ 135°_−x_ 45°_x_ – τ – 90°_y_ 180°_−y_ 90°_y_ 135°_−y_ 45°_y_ – τ/2 – acquisition) was used with an echo delay of τ = 20 μs^38^, a spectral width of 400 kHz, 16384 scans, 4096 points, and a recycle delay of 0.5 s.

#### Analysis

De-Paking was done using a MATLAB (MathWorks, Natick, MA) script developed by the M. Brown group at The University of Arizona. The order parameter (S_CD_) was calculated using the equation below:

$$\Delta {v}_{Q}=\frac{3}{2}X|{S}_{CD}||{P}_{2}(\cos \,\theta )|$$

where $X\equiv ({e}^{2}qQ/h)$ = 167 kHz, *P*_2_ is the second order Legendre polynomial, and θ = 0°, corresponding to the bilayer normal perpendicular with the magnetic field.

## Supplementary information

Supplementary information
