# Supplementary material for: Fusion of Bipolar Tetraether Lipid Membranes Without Enhanced Leakage of Small Molecules
Source: Sci Rep. 2019 Dec 18;9:19359. doi: 10.1038/s41598-019-55494-z (PMC6920354; doi:10.1038/s41598-019-55494-z)
Supplement: Supplementary file 1 — Supplementary information [file 41598_2019_55494_MOESM1_ESM.pdf]

Supporting Information

Fusion of Bipolar Tetraether Lipid Membranes Without Enhanced Leakage of Small Molecules

Geoffray Leriche,[a] Dillan Stengel,[b] David Onofrei,[b] Takaoki Koyanagi,[a] Gregory P. Holland[b] and Jerry Yang[a]*

[a] Department of Chemistry and Biochemistry University of California San Diego, La Jolla, California 92093-0358, (USA), [b] Department of Chemistry and Biochemistry San Diego State University, San Diego, California 92182-1030, (USA)

E-mail: Jerryyang@ucsd.edu

Table of Content

1. Supplemental Figures 2

2. General Information 5

3. Synthesis 6

4. References 19

5. NMR Spectra 21

1. Supplemental Figures

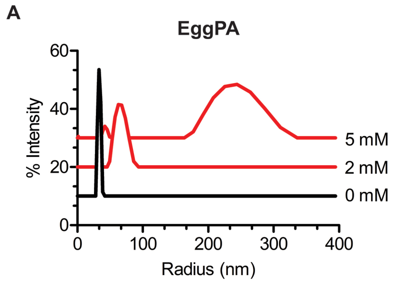

Fig. S1 Hydrodynamic radius of liposomes made of EggPA (A) or GMGTPA (B) measured by dynamic light scattering after content mixing experiments. Experiments were carried at room temperature in TES buffer (10 mM, 2 mM Histidine, 0.1 mM EDTA, NaCl 100 mM, pH 7.4) supplemented with 2 or 5 mM $CaCl_2$. Liposomes in TES buffer without Ca^{2+} were used as controls (black traces). Each sample had been quenched with EDTA (100 mM) prior to measurement in order to stop the aggregation process.

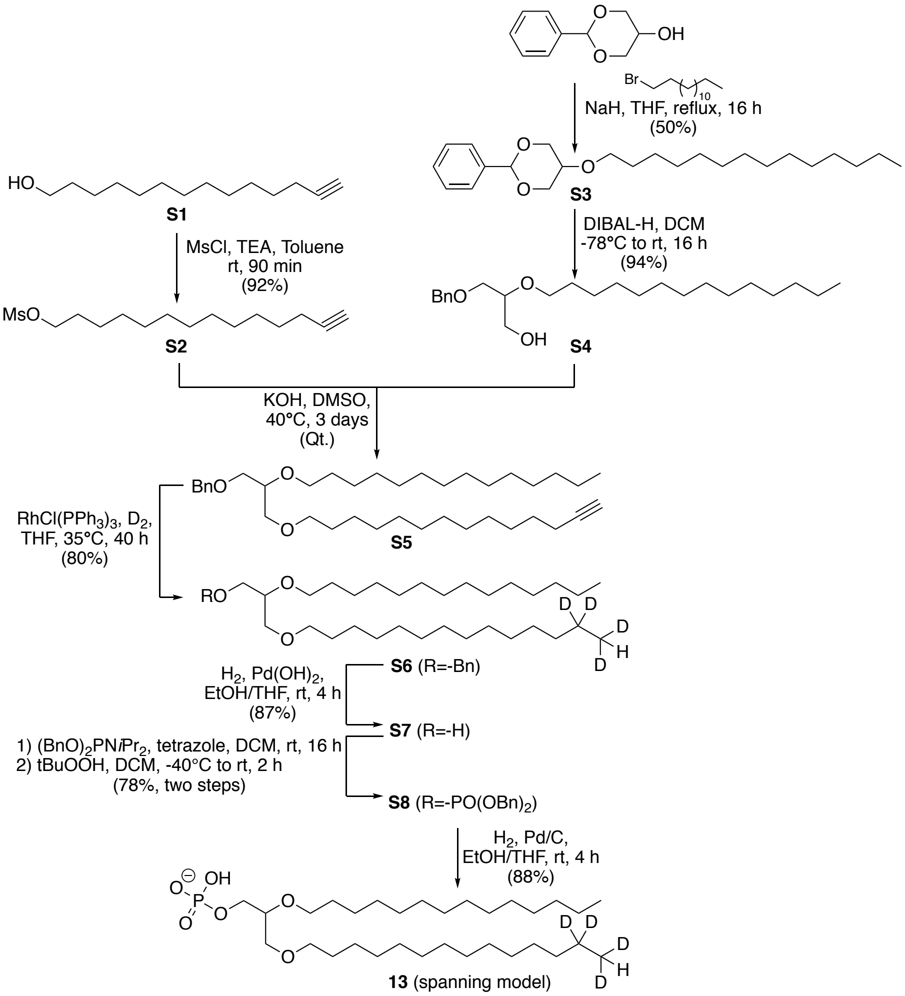

Fig. S2 Synthesis of deuterated lipid **13** (spanning model)

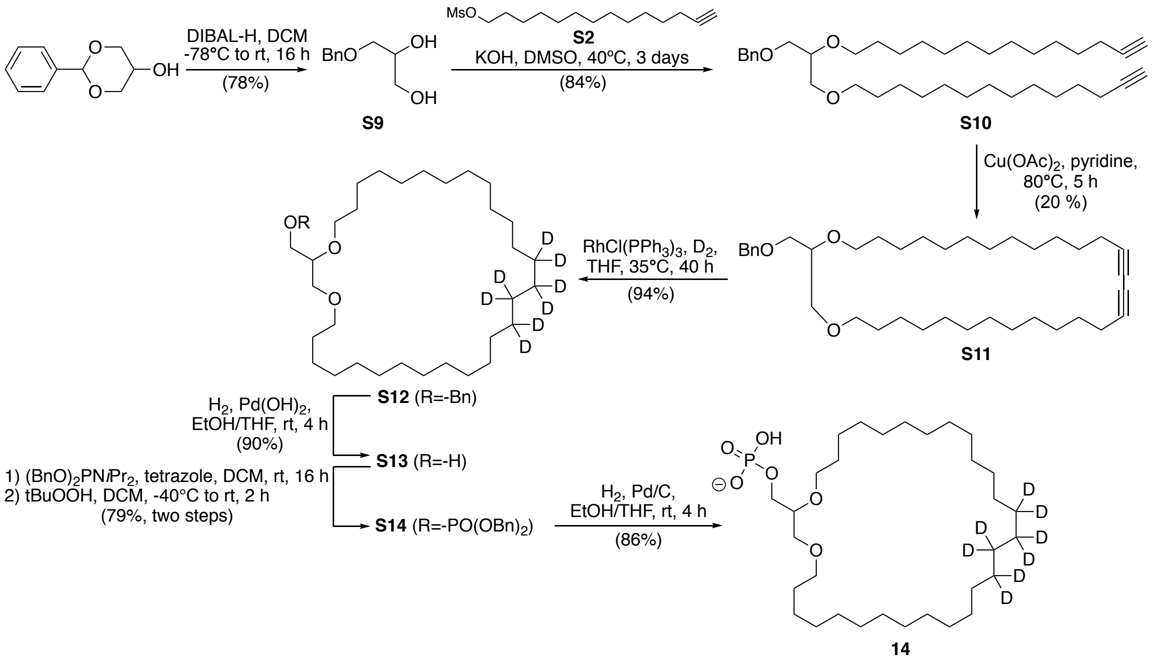

Fig. S3 Synthesis of deuterated lipid **14** (looping model)

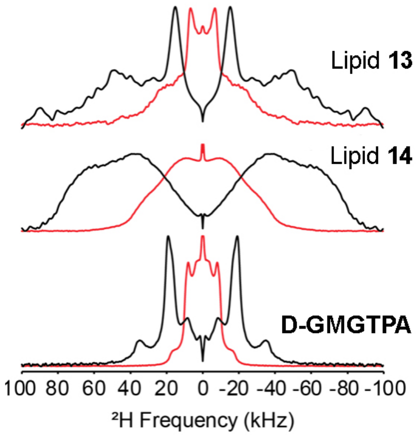

Fig. S4. ^{2}H powder-type (red traces) and de-Paked (black traces) spectra of spanning model (lipid **13**), looping model (lipid **14**), and bipolar tetraether **D-GMGTPA** lipids at 30°C.

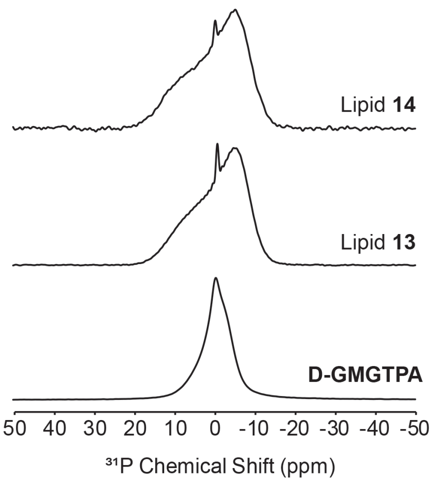

Fig. S5. ^{31}P spectra of looping model (lipid **14**), spanning model (lipid **13**), and bipolar tetraether **D-GMGTPA** at 30°C; 10240 scans, 1024 points, and a recycle delay of 2.5 s. A typical experiment used a 90° pulse width of 3.6μs, and a 412 ppm spectral width. All data were processed with 100 Hz of line broadening.

2. General Information

All reagents were purchased from commercial sources and used without further purification. EggPA (#840101P), PE-NDB (#810144P) and PE-Lissamine (#810158P) lipids were purchased from Avanti Polar Lipids. EggPA lipid was stored under Argon at -20°C and used within 3 months of purchase. Glassware was dried at 115°C overnight. Air and moisture-sensitive reagents were transferred using a syringe or stainless steel cannula. Intermediates were purified over silica (60Å, particle size 40-63 μm) purchased from Dynamic Adsorbents, Inc. Reactions were monitored by thin-layer chromatography (TLC) using 0.25 mm silica gel plates (60F-254) from Dynamic Adsorbents, Inc. Deuterated solvents were purchased from Cambridge Isotope Laboratories, Inc. ^{1}H, ^{2}H, ^{13}C, ^{31}P NMR spectra were obtained on either JEOL ECA 500 spectrometer or Varian 500MHz spectrometer. Chemical shifts are reported in ppm relative to residual solvent. The FID file was analyzed using NMRnotebook version 2.70 build 0.10 by NMRTEC.

Dynamic Light Scattering (DLS) measurements were performed on a Wyatt DynaPro NanoStar (Wyatt Technology, Santa Barbara, CA) instrument using a disposable cuvette (Eppendorf UVette 220 nm – 1,600 nm) and data processed using Wyatt DYNAMICS V7 software. Each analysis involved an average of 10 measurements. The data was exported for final plotting using GraphPad Prism 5 (GraphPad Software, Inc., La Jolla, CA).

Low resolution MS analysis was performed on a Micromass Quattro Ultima triple quadrupole mass spectrometer with an electrospray ionization (ESI) source. High resolution MS analysis was performed using Agilent 6230 Accurate-Mass TOFMS with an electrospray ionization (ESI) source by Molecular Mass Spectrometry Facility (MMSF) in the department of chemistry and biochemistry at University of California, San Diego.

Fluorescence measurements were taken on a Perkin Elmer Enspire multimode plate reader (Corning 96-well, half area, non-treated black polystyrene plates were used). The data were exported for final plotting using GraphPad Prism 5 (GraphPad Software, Inc., La Jolla, CA).

3. Synthesis

3-((28-(3-hydroxy-2-((3,7,11,15-tetramethylhexadecyl)oxy)propoxy)octacosyl) oxy)-2-((3,7,11,15-tetramethylhexadecyl) oxy)propan-1-ol (**1**)

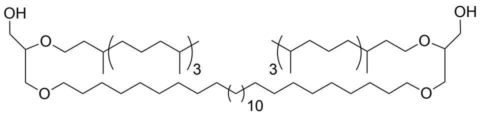

Compound **1** was prepared following a reported protocol.[1]

2-cyanoethyl (3,6,9,12-tetraoxapentadec-14-yn-1-yl) diisopropylphosphoramidite (**2**)

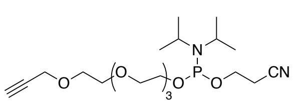

A solution of mono propargylated tetraethylene glycol[2] (0.47 g, 2.03 mmol) in 10 mL of degassed dichloromethane (DCM) and tetrazole (0.45 M in acetonitrile, 4.5 mL) was prepared. Then, a solution of 2-cyanoethyl N,N,N′,N′-tetraisopropylphosphorodiamidite (1.22 g, 4.06 mmol) in 10 mL of degassed DCM was added dropwise and the reaction mixture was stirred at room temperature (rt) for 2 h. The reaction was diluted with DCM and then washed with saturated aqueous $NaHCO_3$ solution, brine and then dried over $MgSO_4$. The solvent was evaporated under vacuum and purified over silica using hexane/ethyl acetate/triethylamine (8:2:0.5 then 0:10:0.5) as the eluent. Phospharamidite **2** was obtained (0.64 g, 73%) as a yellow oil.

Rf: 0.31 (hexane/ethyl acetate/triethylamine, 8:2:0.5); ^{1}H NMR (500 MHz, $CDCl_3$-d_1) δ 4.17 (d, J= 2.4 Hz, 2H), 3.87-3.75 (m, 4H), 3.71-3.55 (m, 18H), 2.62 (ddd, J= 2.4, 6.4, 6.7 Hz, 2H), 2.40 (dd, J= 2.4, 2.5 Hz, 1H), 1.29-1.24 (m, 2H), 1.18-1.13 (m, 12H); ^{13}C NMR (126 MHz, $CDCl_3$-d_1) δ 117.9, 79.8, 74.7, 71.4, 71.3, 70.8, 70.6, 69.3, 62.8, 62.7, 58.8, 58.6, 58.5,43.3, 43.2, 24.8, 24.8, 24.7, 23.6, 23.0, 20.5, 20.4; ^{31}P NMR (202 MHz, $CDCl_3$-d_1) δ 149.2.

Dialkyne phosphate triester (**3**)

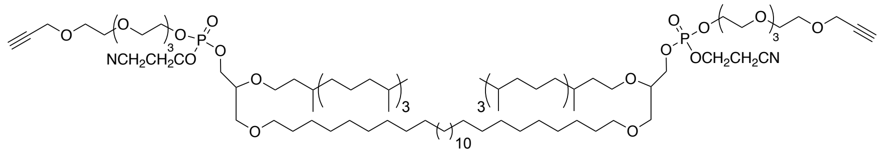

A solution of **1** (95 mg, 0.08 mmol) in 2 mL of degassed dichloromethane (DCM) and tetrazole (0.45 M in acetonitrile, 1.7 mL) was prepared. Then, a solution of **2** (181 mg, 0.4 mmol) in 2 mL of degassed DCM was added dropwise and the reaction mixture was stirred at room temperature for 16 h. After the reaction was cooled to -40°C, a tert-Butyl hydroperoxide solution (5-6 M in decane, 0.3 mL) was added and the reaction mixture was allowed to warm up to rt over 2 h. The solvent was evaporated over vacuum and the resulting residue was purified over silica using ethyl acetate/methanol (EtOAc/MeOH) (95:5) as the eluent. Phosphate triester **3** was obtained as a clear oil (82 mg, 53%).

Rf: 0.43 (EtOAc/MeOH, 95:5); ^{1}H NMR (500 MHz, $CDCl_3$-d_1) δ 4.25-4.05 (m, 16H), 3.69-3.39 (m, 42H), 2.76 (d, J= 6.4 Hz, 4H), 2.42-2.40 (m, 2H), 1.57-1.44 (m, 10H), 1.33-1.02 (m, 90H), 0.83-0.80 (m, 30H); ^{13}C NMR (126 MHz, $CDCl_3$-d_1) δ 116.8, 79.7, 79.7, 74.7, 72.7, 71.9, 70.7, 70.6, 70.5, 70.5, 70.4, 70.0, 69.5, 69.6, 69.2, 69.1, 69.0, 67.5, 67.3, 67.3, 67.2, 62.0, 62.0, 61.7, 58.5, 39.4, 37.7, 37.6, 37.5, 37.437.4, 37.2, 37.1, 32.9, 29.9, 29.8, 29.7, 29.6, 28.0, 26.2, 24.9, 24.6, 24.5, 22.8, 22.7, 19.8, 19.8, 19.7, 19.6, 19.6; ^{31}P NMR (202 MHz, $CDCl_3$-d_1) δ -0.87; ESI-MS: 1830.3 $[M+H]^+$ and 1852.4 $[M+Na]^+$; HRMS: 1852.3592 calcd for $[C_{102}H_{194}N_2O_{20}P_2Na]^+$ 1852.3586 found.

tert-butyl (2-(2-azidoethoxy)ethyl)carbamate (**t-Boc-N-Amido-PEG1-azide**)

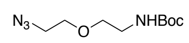

Linker **t-Boc-N-Amido-PEG1-azide** was prepared following a reported protocol.[3]

NBD-N_3

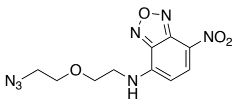

Trifluoracetic acid (0.2 mL) was added to a solution of **t-Boc-N-Amido-PEG1-azide** (99 mg, 0.43 mmol) in DCM (1.8 mL), and the reaction mixture was stirred at rt for 4 h. Upon completion of the reaction, excess acid and solvent was removed under vacuum, and the residue was dried in vacuo overnight. The resulting product was used without further purification and dissolved in dry tetrahydrofuran (THF) (5 mL), and then NBD-Cl (90 mg, 0.45 mmol) and triethylamine (1.29 mmol, 0.2 mL) were added successively. The reaction was allowed to proceed with stirring at rt for 1 h and removal of the solvent in vacuo gave a brown liquid which was further purified over silica using hexane/EtOAc (75:25 to 25:75) as eluent. **NBD-N_3** (51 mg, 40%) was obtained as a brown oil.

Rf: 0.68 (hexane/EtOAc, 1:1); ^{1}H NMR (500 MHz, $CDCl_3$-d_1) δ 8.42 (d, J= 8.7 Hz, 1H), 6.75 (brs, 1H), 6.21 (d, J= 8.7 Hz, 1H), 3.85 (d, J= 5.2 Hz, 2H), 3.74-3.71 (m. 4H), 3.49 (t, J= 4.9 Hz, 2H); ^{13}C NMR (126 MHz, $CDCl_3$-d_1) δ 144.4, 144.3, 144.0, 136.7, 124.0, 99.2, 70.4, 68.6, 50.8, 43.8; ESI-MS: 294.2 $[M+H]^+$.

Rho-N_3

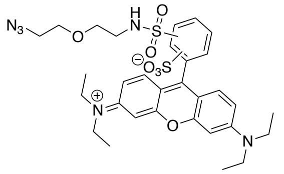

Trifluoracetic acid (0.2 mL) was added to a solution of **t-Boc-N-Amido-PEG1-azide** (97 mg, 0.42 mmol) in DCM (1.8 mL), and the reaction mixture was stirred at rt for 4 h. Upon completion of the reaction, excess acid and solvent was removed under vacuum, and the residue was dried in vacuo overnight. The resulting product was used without further purification and dissolved in a mixture of dry DCM and dimethylformamide (DMF) (4 mL, 1:5). Then, lissamine rhodamine B sulfonyl chloride (219 mg, 0.38 mmol), 4-dimethylaminopyridine (5 mg, 0.04 mmol) and N,N-diisopropylethylamine (2.28 mmol, 0.4 mL) were added successively. The reaction was allowed to proceed with

stirring at rt for 16 h and removal of the solvent in vacuo gave a dark liquid which was further purified over silica using DCM/MeOH (9:1) as eluent. An isomeric mixture of **Rho-N$_3$** (62 mg, 22%) was obtained as a purple solid.

Rf: 0.43 (DCM/MeOH, 95:5); ^{1}H NMR (500 MHz, MeOD-d$_4$/CDCl$_3$-d$_1$ 1:1) δ 8.82 (s, 0.6H), 8.73 (s, 0.4H), 8.36 (dd, *J*= 7.9, 1.6 Hz, 0.4H); 8.20 (dd, *J*= 7.9, 1.6 Hz, 0.6H); 7.51 (d, *J*= 7.6 Hz, 1H), 7.29-7.24 (m, 2H), 7.08-7.01 (m, 2H), 6.98-6.94 (m, 2H), 3.89-3.66 (m, 11H), 3.63-3.47 (m, 2.4H), 3.42-3.40 (m, 0.6H), 3.37-3.18 (m, 2H), 1.47-1.38 (m, 12H); ^{13}C NMR (126 MHz, MeOD-d$_4$/CDCl$_3$-d$_1$ 1:1) δ 158.8, 158.7, 157.7, 156.6, 156.5, 148.9, 147.0, 143.3, 142.0, 134.8, 133.7, 133.4, 132.7, 131.9, 131.6, 130.4, 128.7, 127.5, 127.1, 114.9, 114.5, 96.9, 96.9, 70.6, 70.5, 51.4, 51.3, 46.7, 46.6, 43.6, 43.5, 12.9; ESI-MS: 671.1 $[M+H]^+$ 693.1 $[M+Na]^+$.

Protected NBD lipid (**4**)

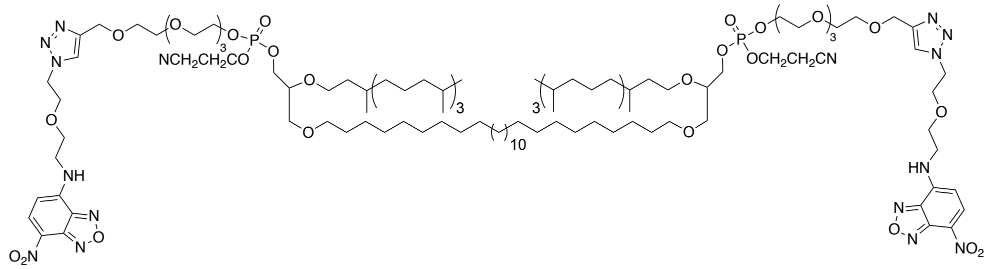

To a round bottom flask, compound **3** (17 mg, 0.09 mmol), copper (**II**) sulfate (1.3 mg, 0.09 mmol), sodium ascorbate (1.8 mg, 00.09 mmol), tris[(1-benzyl-1H-1,2,3-triazol-4-yl)methyl]amine (0.5 mg) and **NBD-N$_3$** (11 mg, 0.04 mmol) were dissolved in 2 mL of a mixture of DMF/THF/water (2:1:1). The reaction was stirred overnight and solvent was removed in vacuo. The resulting residue was purified first by column chromatography on Sephadex LH 20 (DCM/MeOH 1:1). Then, a second purification over silica using EtOAc/MeOH (95:5 to 85:15) as eluent gave compound **4** (15 mg, 63%) as an orange solid.

Rf: 0.58 (EtOAc/MeOH, 9:1); ^{1}H NMR (500 MHz, MeOD-d$_4$/CDCl$_3$-d$_1$ 1:1) δ 8.17 (d, *J*= 8.7 Hz, 2H), 7.49 (s, 2H), 5.97 (d, *J*= 8.7 Hz, 2H), 4.25-4.23 (m, 8H), 3.98-3.86 (m, 10H), 3.80-3.75 (m, 2H), 3.59 (t, *J*= 4.9 Hz, 4H), 3.46-3.28 (42H), 3.19-3.12 (m, 8H), 2.54 (t, *J*= 5.9 Hz, 4H), 1.29-1.18 (m, 10H), 1.06-0.90 (m, 76H), 0.82-0.72 (m, 14H), 0.56-0.51 (m, 30H); ^{31}P NMR (202 MHz, CDCl$_3$-d$_1$) δ -1.34; ESI-MS: 1231.1 $[M+2Na]^{2+}$; HRMS: 1230.7615 calcd for $[C_{122}H_{216}N_{16}O_{28}P_2Na_2]^{2+}$ 1230.7601 found.

Protected Rhodamine lipid (**5**)

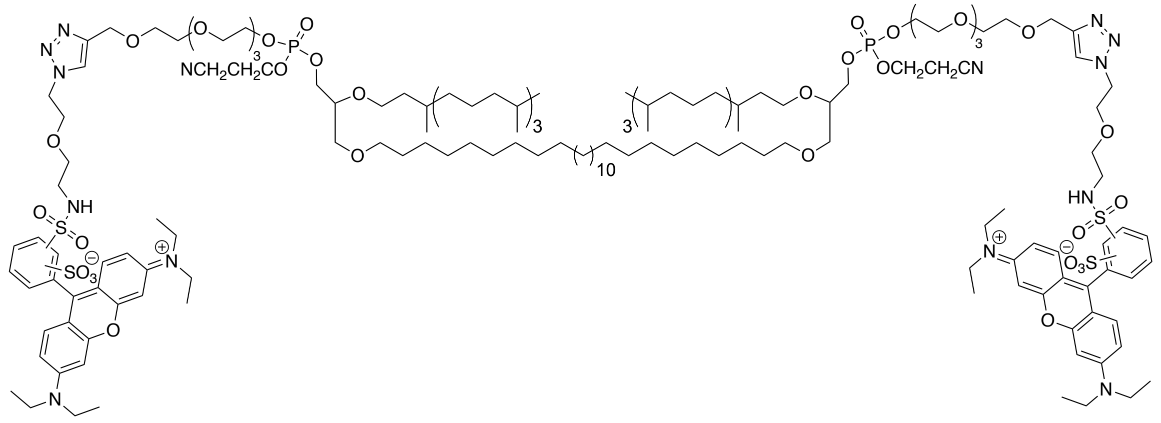

Compound **5** was prepared following the protocol used for the synthesis of **4**. Crude was successively purified on Sephadex LH 20 (DCM/MeOH 1:1) and silica (DCM/MeOH 9:1) to give protected lipid **5** (22 mg, 69%) as a purple solid.

Rf: 0.21 (DCM/MeOH, 95:15); ^{1}H NMR (500 MHz, MeOD-d_4/$CDCl_3$-d_1 1:1) δ 8.53-8.26 (m, 2H), 7.90 (dd, *J*= 7.9, 1.6 Hz, 0.9H), 7.71 (dd, *J*= 7.9, 1.6 Hz, 1.1H), 7.64-7.61 (m, 2H), 7.04-7.01 (m, 2H), 6.79-6.77 (m, 4H), 6.62-6.46 (m, 8H), 4.26-4.18 (m, 6H), 3.94-3.84 (m, 10H), 3.78-3.74 (m, 2H), 3.51-3.44 (m, 4H), 3.39-3.23 (m, 52H), 3.21-3.02 (m, 14H), 2.89 (t, *J*= 4.9 Hz, 2H), 2.80-2.71 (m, 2H), 2.53-2.51 (m, 4H), 1.28-1.15 (m, 10H), 1.04-0.86 (m, 98H), 0.83-0.71 (m, 16H), 0.55-0.49 (m, 30H); ^{31}P NMR (202 MHz, $CDCl_3$-d_1) δ -1.37; ESI-MS: 1586.2 $[M+2H]^{2+}$ 1608.6 $[M+2Na]^{2+}$; HRMS: 1607.8986 calcd for $[C_{164}H_{270}N_{14}O_{34}P_2S_4Na_2]^{2+}$1607.8967 found.

NBD lipid (**GMGTNBD**)

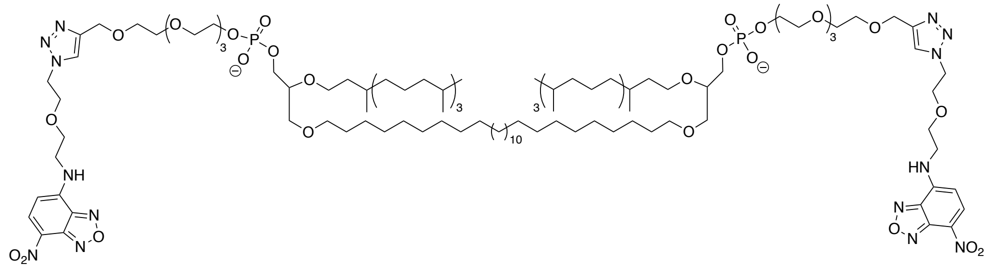

To a solution of **4** (15 mg, 0.006 mmol) in pyridine (1.8 mL) was added diethylamine (0.2 mL) and the reaction was stirred at rt for 6 h. Upon completion of the reaction, solvent was removed under vacuum, and the residue was successively purified on silica (DCM/MeOH 9:1 to 6:4) and Sephadex LH 20 (DCM/MeOH 1:1). Lipid **4** (13 mg, 88%) was obtained as an orange solid.

^{1}H NMR (500 MHz, MeOD-d_4/$CDCl_3$-d_1 1:1) δ 8.19-8.17 (m, 2H), 7.55 (s, 2H), 5.99-5.98 (m, 2H), 4.29-4.25 (m, 6H), 3.71-3.14 (m, 66H), 1.32-1.23 (m, 10H), 1.06-0.76 (m, 90H), 0.57-0.53 (m, 30H); ^{31}P NMR (202 MHz, MeOD-d_4/$CDCl_3$-d_1 1:1) δ -1.4; ESI-MS: 1199.9 $[M-2H+4Na]^{2+}$; HRMS: 1177.7349 calcd for $[C_{116}H_{210}N_{14}O_{28}P_2Na_2]^{2+}$ 1177.7337 found.

Rhodamine lipid (**GMGTRho**)

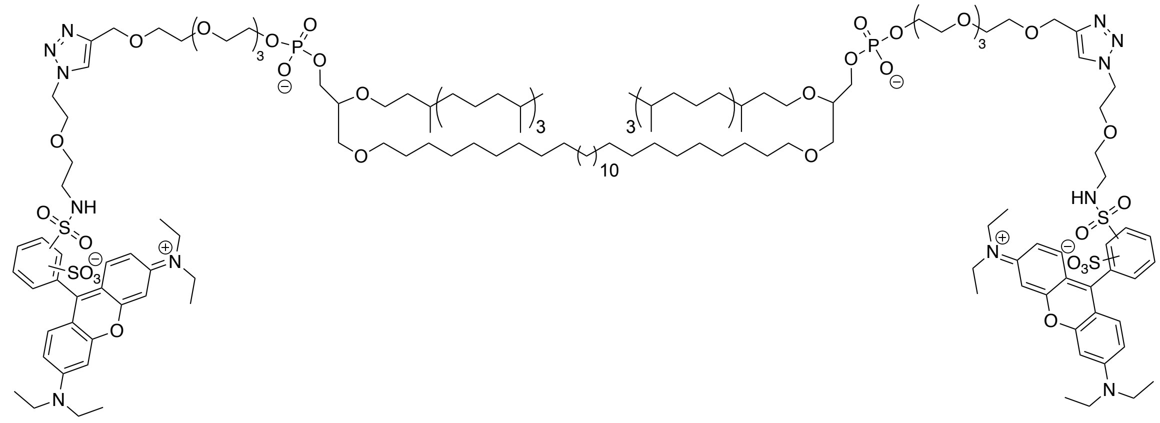

Lipid **GMGTRho** was prepared following the protocol used for the synthesis of **GMGTNBD**. Crude was successively purified on silica (DCM/MeOH 95:5 to 7:3) and Sephadex LH 20 (DCM/MeOH 1:1) to give **GMGTRho** (20 mg, 94%) as a purple solid.

^{1}H NMR (500 MHz, MeOD-d_4/$CDCl_3$-d_1 1:1) δ 8.36-8.28 (m, 2H), 7.92-7.91 (m, 1H), 7.74-7.65 (m, 3H), 7.04 (t, *J*= 7.9, 2H), 6.63-6.48 (m, 8H), 4.27-4.18 (m, 4H), 3.73-3.03 (m, 74H), 2.91-2.89 (m, 2H), 2.78-2.69 (m, 2H), 1.28-1.15 (m, 10H), 1.01-0.73 (m, 114H), 0.55-0.48 (m, 30H); ^{31}P NMR (202 MHz, MeOD-d_4/$CDCl_3$-d_1 1:1) δ -2.2; ESI-MS: 1578.0 $[M-2H+4Na]^{2+}$; HRMS: 1576.8540 calcd for $[C_{158}H_{262}N_{12}O_{34}P_2S_4Na_4]^{2+}$ 1576.8529 found.

1,28-bis((tetrahydro-2H-pyran-2-yl)oxy)octacosa-13,15-diyne (**6**)

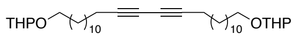

Compound **6** was prepared from dodecandiol following a reported protocol.[1]

1,28-bis((tetrahydro-2H-pyran-2-yl)oxy)octacosane-13,13,14,14,15,15,16,16-d_8 (**7**)

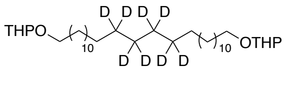

Diyne **6** (1.55 g, 2.65 mmol) was dissolved in degassed THF (26 mL) and Wilkinson catalyst (0.49 g, 0.53 mmol) was added. The reaction was stirred under deuterium atmosphere at 30 °C for 40 h. Solvent was evaporated and the resulting residue was purified by silica gel column chromatography using hexane/EtOAc (100:0 to 95:5) as eluent. Compound **7** (1.20 g, 75%) was obtained as a white solid.

Rf: 0.38 (hexane/EtOAc 95:5); ^{1}H NMR (500 MHz, $CDCl_3$-d_1) δ 4.54 (dd, *J*= 4.4, 2.8 Hz, 2H), 3.85-3.81 (m, 2H), 3.71-3.67 (m, 2H), 3.48-3.44 (m, 2H), 3.36-3.31 (m, 2H), 1.82-1.76 (m, 2H), 1.70-1.65 (m, 2H), 1.57-1.46 (m, 12H), 1.32-1.21 (m, 40H); ^{13}C NMR (126 MHz, $CDCl_3$-d_1) δ 99.0, 67.9, 62.5, 31.0, 29.9, 29.9, 29.9, 29.9, 29.8, 29.7, 29.6, 29.0-28.4 (m), 26.4, 25.7, 19.9; ^{2}H NMR (100 MHz, $CDCl_3$-d_1) δ 1.16 (brs, 8D). ESI-MS: 623.6 $[M-2H+Na]^{-}$; HRMS: 625.5981 calcd for $[C_{38}H_{66}D_8O_4Na]^{+}$ 625.5981 found.

1,28-dibromooctacosane-13,13,14,14,15,15,16,16-d_8 (**8**)

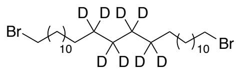

Carbon tetrabromide (2.40 g, 7.10 mmol) was added to a suspension of **7** (1.42 g, 2.35 mmol) in dry DCM (36 mL). After stirring for 10 min, the solution was cooled down and triphenylphosphine (3.70 g, 14.10 mmol) was added. The mixture was stirred 24 h at rt and purified by silica gel column chromatography using DCM/hexane (1:1) as the eluent. Dibromoalkane **8** (1.17 g, 89%) was obtained as a white solid.

Rf: 0.90 (DCM/hexane 1:1); ^{1}H NMR (500 MHz, $CDCl_3$-d_1) δ 3.38 (t, J= 6.9 Hz, 4H), 1.86-1.81 (m, 4H), 1.43-1.38 (m, 4H), 1.30-1.24 (m, 36H); ^{13}C NMR (126 MHz, $CDCl_3$-d_1) δ 90.6, 89.5, 86.3, 86.3, 82.2, 86.1, 85.4, 84.9; ^{2}H NMR (100 MHz, $CDCl_3$-d_1) δ 1.19 (brs, 8D).

3-(benzyloxy)-2-((3,7,11,15-tetramethylhexadecyl)oxy)propan-1-ol (**9**)

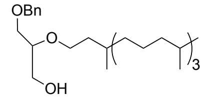

Compound **9** was synthesized following a reported protocol.[4]

18,51-bis((benzyloxy)methyl)-2,6,10,14,55,59,63,67-octamethyl-17,20,49,52-tetraoxaoctahexacontane-33,33,34,34,35,35,36,36-d_8 (**10**)

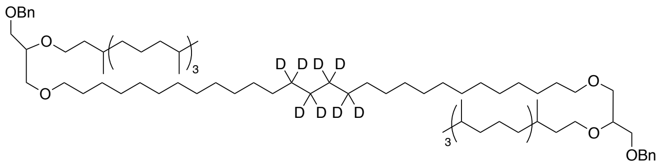

To a cold solution of **9** (2.14 g, 4.63 mmol) in dry THF (21 mL), sodium hydride (0.30 g, 12.50 mmol) was added portionwise. The solution was stirred for 1 h at room temperature and cooled down again. A solution of dibromoalkane **8** (1.04 g, 1.86 mmol) in dry THF (21 mL) was added and the reaction was stirred at reflux for 16 h. The reaction was quenched with water and the solvent was removed under vacuum. The aqueous residue was extracted with DCM (3x50 mL), washed successively with water (1x100 mL) and brine (1x100 mL), then dried over Na_2SO_4. The resulting residue was purified by column chromatography on silica gel using hexane/EtOAc (100:0 to 95:5) as eluent and compound **10** (0.78 g, 32%) was obtained as a clear oil.

Rf: 0.26 (hexane/EtOAc 95:5); ^{1}H NMR (500 MHz, $CDCl_3$-d_1) δ 7.33-7.24 (m, 10H), 4.55 (s, 4H), 3.64-3.41 (m, 18H), 1.65-1.49 (m, 10H), 1.39-1.04 (m, 82H), 0.87-0.83 (m, 30H); ^{13}C NMR (126 MHz, $CDCl_3$-d_1) δ 138.6, 128.5, 127.7, 127.6, 78.1, 73.5, 71.8, 70.9, 70.4, 69.0, 39.5, 37.7, 37.6, 37.6, 37.5, 37.5, 37.3, 37.3, 33.0. 29.9, 29.8, 29.7, 29.0-28.6 (m), 28.1, 26.3, 25.0, 24.7, 24.6, 22.9, 22.8, 19.9, 19.9, 19.8; ^{2}H NMR (100 MHz, $CDCl_3$-d_1) δ 1.20 (brs, 8D).; ESI-MS: 1341.3 $[M+NH_4]^+$; HRMS: 1341.3212 calcd for $[C_{88}H_{154}D_8O_6NH_4]^+$ 1341.3200 found.

3-((28-(3-hydroxy-2-((3,7,11,15-tetramethylhexadecyl)oxy)propoxy)octacosyl-13,13,14,14,15,15,16,16-*d*8)oxy)-2-((3,7,11,15-tetramethylhexadecyl)oxy)propan-1-ol (**11**)

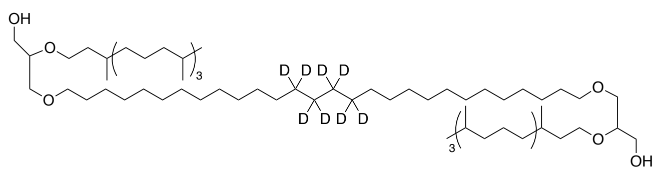

Compound **10** (583 mg, 0.44 mmol) was dissolved in a degassed mixture of EtOH/THF (1:1, 30 mL) and 20% $Pd(OH)_2$ (58 mg, 10% w/w) was added. The reaction was stirred under hydrogen atmosphere at room temperature for 6 hours. The catalyst was removed by filtration through a pad of celite, and the resulting residue was purified by column chromatography on silica gel using hexane/EtOAc (9:1 to 8:2) as the eluent. Diol **11** (409 mg, 81%) was obtained as a white solid.

Rf: 0.52 (hexane/EtOAc 8:2); ^{1}H NMR (500 MHz, $CDCl_3$-d_1) δ 3.67-3.65 (m, 18H), 2.38 (s, 2H), 1.59-1.44 (m, 10H), 1.34-1.01 (m, 82H), 0.83-0.79 (m, 30H); ^{13}C NMR (126 MHz, $CDCl_3$-d_1) δ 78.6, 72.0, 71.1, 71.0, 68.8, 63.2, 63.1, 39.5, 37.7, 37.6, 37.5, 37.5, 37.5, 37.5, 37.4, 37.3, 37.2, 33.0, 32.9, 30.0, 30.0, 29.9, 29.9, 29.9, 29.9, 29.9, 29.8, 29.8, 29.7, 28.1, 26.3, 25.0, 25.0, 24.7, 24.6, 24.5, 22.9, 22.8, 19.9, 19.9, 19.8, 19.8, 19.8; ^{2}H NMR (100 MHz, $CDCl_3$-d_1) δ 1.17 (brs, 8D); ESI-MS: 1143.8 $[M+H]^+$; HRMS: 1144.2007 calcd for $[C_{74}H_{143}D_8O_6]^+$ 1144.2005 found.

tetrabenzyl (((octacosane-1,28-diyl-13,13,14,14,15,15,16,16-*d*8)bis(oxy))bis(2-((3,7,11,15-tetramethylhexadecyl)oxy)propane-3,1-diyl)) bis(phosphate) (**12**)

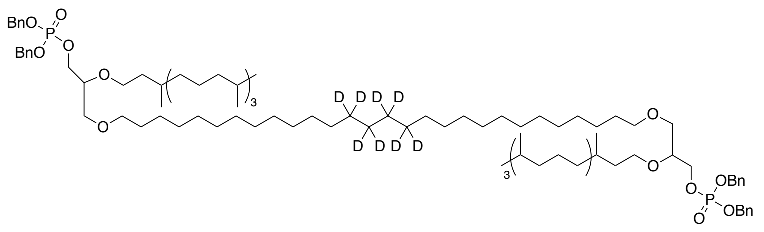

A solution of **11** (51 mg, 0.044 mmol) in degassed DCM (1 mL) and tetrazole (0.45 M in acetonitrile, 0.8 mL) was prepared. Then, a solution of dibenzyl N,N-diisopropylphosphoramidite (61 mg, 0.178 mmol) in degassed DCM (1 mL) was added dropwise and the reaction mixture was stirred at room temperature for 16 h. After the reaction was cooled to -40°C, a tert-Butyl hydroperoxide solution (5-6 M in decane, 0.2 mL) was added and the reaction mixture was allowed to warm up to rt over 2 h. The solvent was evaporated over vacuum and the resulting residue was purified over silica using hexane/EtOAc (9:1 to 6:4) as the eluent. Compound **12** was obtained as a clear oil (67 mg, 87%).

Rf: 0.41 (hexane/EtOAc 7:3); ^{1}H NMR (500 MHz, $CDCl_3$-d_1) δ 7.33-7.31 (m, 20H), 5.05-5.02 (m, 8H), 4.12-4.08 (m, 2H), 4.03-3.98 (m, 2H), 3.56-3.49 (m, 6H), 3.42-3.36 (m, 8H), 1.57-1.46 (m, 10H), 1.33-1.03 (m, 82H), 0.85-0.80 (m, 30H); ^{13}C NMR (126 MHz, $CDCl_3$-d_1) δ 136.1, 136.0, 128.7, 128.7, 128.1, 100.1, 72.0, 69.9, 69.4, 63.4, 69.2, 69.2, 67.3, 67.2, 39.6, 37.8, 37.7, 37.6, 37.5, 37.3, 37.2, 33.0, 30.0, 29.9, 29.8, 29.7, 26.3, 25.0, 24.7, 24.6, 22.9, 22.8, 20.0, 19.9, 19.8, 19.8,

19.7; ^{31}P NMR (202 MHz, $CDCl_3$-d_1) δ -0.32; ESI-MS: 1665.0 $[M+H]^+$; HRMS: 1664.3212 calcd for $[C_{102}H_{169}D_8O_{12}P_2]^+$ 1664.3186 found.

((octacosane-1,28-diyl-13,13,14,14,15,15,16,16-*d*8)bis(oxy))bis(2-((3,7,11,15-tetramethylhexadecyl)oxy)propane-3,1-diyl) bis(hydrogen phosphate) (**D-GMGTPA**)

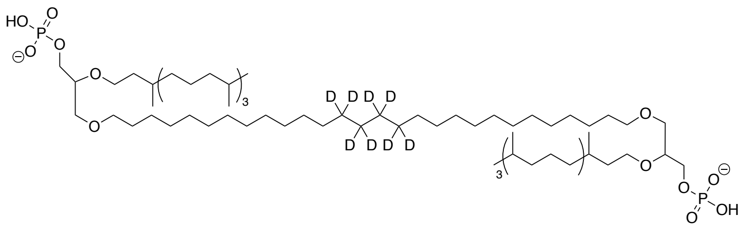

Protected lipid **12** (328 mg, 0.20 mmol) was dissolved in a degassed mixture of EtOH/THF (2:1, 36 mL) and Pd/C (60 mg, 20% w/w) was added. The reaction was stirred under hydrogen atmosphere at room temperature for 4 h. The catalyst was removed by filtration through a pad of celite, and the resulting residue was purified on Sephadex LH 20 (DCM/MeOH 1:1). Lipid **D-GMGTPA** (229 mg, 89%) was obtained as a colorless oil.

Rf: 0.02 (DCM/MeOH 8:2); ^{1}H NMR (500 MHz, MeOD-d_4/$CDCl_3$-d_1 1:1) δ 3.98-3.97 (m, 4H), 3.66-3.43 (m, 14H), 1.60-1.48 (m, 10H), 1.38-1.03 (m, 82H), 0.87-0.82 (m, 30H); ^{13}C NMR (126 MHz, MeOD-d_4/$CDCl_3$-d_1 1:1) δ 77.5, 70.9, 69.8, 69.6, 66.3, 40.1, 38.3, 38.2, 38.1, 38.1, 38.0, 37.8, 37.7, 37.6, 33.5, 30.5, 30.4, 30.3, 30.3, 30.2, 30.1, 28.7, 26.8, 25.5, 25.1, 25.1, 23.1, 23.0, 20.2, 20.2, 20.1, 20.0, 20.0; ^{31}P NMR (202 MHz, MeOD-d_4/$CDCl_3$-d_1 1:1) δ 0.70; ^{2}H NMR (100 MHz, MeOD-d_4/$CDCl_3$-d_1 1:1) δ 1.17 (brs, 8D); ESI-MS: 1302.1 $[M-H]^-$; HRMS: 1302.1188 calcd for $[C_{74}H_{143}D_8O_{12}P_2]^-$ 1302.1168 found.

tetradec-13-yn-1-ol (**S1**)

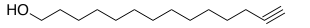

Compound **S1** was prepared from dodecandiol following a reported protocol.[1]

tetradec-13-yn-1-yl methanesulfonate (**S2**)

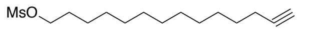

A cold solution of **S1** (1.43 g, 6.80 mmol) in toluene (9 mL) and TEA (0.95 mL, 6.8 mmol) was prepared. Then, methanesulfonyl chloride (0.53 mL, 6.8 mmol) was added dropwise and the reaction mixture was stirred at room temperature for 90 minutes. Water (50 mL) was added and the reaction mixture was extracted with EtOAc (3x50 mL), over Na_2SO_4. The resulting residue was purified by column chromatography on silica gel using hexane/EtOAc (9:1 to 8:2) as eluent and compound **S2** (1.80 g, 93%) was obtained as a white solid.

Rf: 0.45 (hexane/EtOAc, 8:2); ^{1}H NMR spectrum matched previously reported data.[5]

2-(cyclohexa-2,4-dien-1-yl)-5-(tetradecyloxy)-1,3-dioxane (**S3**)

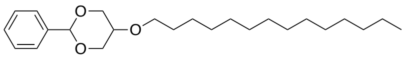

To a cold solution of 1,3-O-benzylideneglycerol (1.66 g, 9.2 mmol) in dry THF (20 mL), sodium hydride (0.62 g, 25.8 mmol) was added portionwise. The solution was stirred for 1 h at room temperature and cooled down again. A solution of 1-bromotetradecane (3.01 g, 11.0 mmol) in dry THF (16 mL) was added and the reaction was stirred at reflux for 16 h. The reaction was quenched with water and the solvent was removed under vacuum. The aqueous residue was extracted with DCM (3x50 mL), washed successively with water (1x100 mL) and brine (1x100 mL), then dried over Na_2SO_4. The resulting residue was purified by column chromatography on silica gel using hexane/EtOAc (98:2 to 95:5) as eluent and compound **S3** (1.88 g, 50%) was obtained as a white solid.

Rf: 0.22 (hexane/EtOAc 95:5); ^{1}H NMR (500 MHz, $CDCl_3$-d_1) δ 7.52-7.50 (m, 2H), 7.35-7.30 (m, 3H), 5.53 (s, 1H), 4.32-4.30 (m, 2H), 4.04-4.00 (m, 2H), 3.53 (t, *J*= 6.8 Hz, 2H), 3.23-3.22 (1H), 1.67-1.61 (m, 2H), 1.38-1.26 (m, 22H), 0.88 (t, *J*= 6.9 Hz, 2H); ^{13}C NMR (126 MHz, $CDCl_3$-d_1) δ 138.4, 128.9, 128.3, 126.3, 101.4, 70.7, 69.2, 69.1, 32.1, 29.9, 29.8, 29.8, 29.6, 29.5, 26.3, 22.8, 14.3; ESI-MS: 377.5 $[M+H]^+$.

3-(benzyloxy)-2-(tetradecyloxy)propan-1-ol (**S4**)

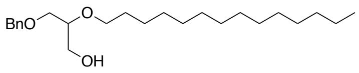

A solution of **S3** (1.73 g, 4.25 mmol) in dry DCM (11 mL) was cooled to -78°C. Then, DIBAL-H (10.6 mmol) in DCM (1M, 11 mL) was added dropwise to the cold solution and reacted for 16 h at rt. After the reaction was complete, a small amount of MeOH was used to carefully quench the remaining DIBAL-H followed by the addition of aqueous NaOH (5 M, 15 mL). The solution was then extracted using diethylether (Et_2O) (3x50 mL) and then washed with water (1x100 mL). The extracted organic layer was dried using Na_2SO_4 and the solvent was removed under vacuum. The crude was purified over silica using hexane/EtOAc (8:2) as the eluent and alcohol **S4** (1.51 g, 94%) was obtained as a clear oil.

Rf: 0.31 (hexane/EtOAc 8:2); ^{1}H NMR (500 MHz, $CDCl_3$-d_1) δ 7.31-7.24 (m, 5H), 4.51 (s, 2H), 3.71-3.49 (m, 7H), 2.67 (s, 1H), 1.57-1.55 (m, 2H), 1.34-1.21 (m, 22H), 0.88 (t, *J*= 6.7 Hz, 3H); ^{13}C NMR (126 MHz, $CDCl_3$-d_1) δ 138.0, 128.3, 127.5, 127.5, 78.7, 73.4, 70.4, 69.9, 62.5, 31.9, 30.0, 29.7, 29.7, 29.6, 29.6, 29.6, 29.5, 29.3, 26.0, 22.6,14.1; ESI-MS: 379.2 $[M+H]^+$ and 396.2 $[M+NH_4]^+$; HRMS: 401.3026 calcd for $[C_{24}H_{42}O_3Na]^+$ 401.3021 found.

((3-(tetradec-13-yn-1-yloxy)-2-(tetradecyloxy)propoxy)methyl)benzene (**S5**)

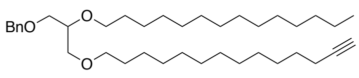

A suspension of KOH (0.42 g, 7.60 mmol) in dry DMSO (24 mL) was stirred at room temperature for 30 minutes. The mixture was cooled with ice water and a solution of **S4** (0.95 g, 2.52 mmol) and **S2** (1.09 g, 3.78 mmol) in dry DMSO (24

mL) was added. The mixture was then stirred at 40 °C for 3 days. Water (500 mL) was added and the mixture was extracted with EtOAc (5x100 mL). The combined organic layers were washed with water (2x200 mL), brine (200 mL) and dried over Na_2SO_4. Purification by silica gel column chromatography using hexane/EtOAc (95:5) as the eluent yielded **S5** (1.52 g, Qt.) as a colorless oil.

Rf: 0.43 (hexane/EtOAc, 95:5); ^{1}H NMR (500 MHz, $CDCl_3$-d_1) δ 7.45-7.27 (m, 5H), 4.57 (s, 2H), 3.63-3.43 (m, 9H), 2.19 (td, *J*= 7.1, 2.7 Hz, 2H), 1.94 (t, *J*= 2.7 Hz, 2H), 1.60-1.50 (m, 6H), 1.42-1.25 (m, 38H), 0.90 (t, *J*= 6.9 Hz, 3H); ^{13}C NMR (126 MHz, $CDCl_3$-d_1) δ 138.6, 128.4, 127.7, 127.6, 84.8, 78.1, 73.5, 71.8, 70.9, 70.7, 70.4, 68.2, 32.1, 30.3, 29.9, 29.8, 29.8, 29.7, 29.5, 29.3, 28.9, 28.6, 26.3, 22.9, 18.5, 14.3; ESI-MS: 571.5 $[M+H]^+$ and 588.4 $[M+NH_4]^+$; HRMS: 593.4904 calcd for $[C_{38}H_{66}O_3Na]^+$ 593.4906 found.

((3-((tetradecyl-13,13,14,14-*d*4)oxy)-2-(tetradecyloxy)propoxy)methyl)benzene (**S6**)

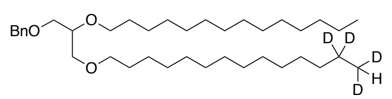

Alkyne **S5** (1.52 g, 2.62 mmol) was dissolved in degassed THF (34 mL) and Wilkinson catalyst (0.42 g) was added. The reaction was stirred under deuterium atmosphere at 35 °C for 40 h. Solvent was evaporated and the resulting residue was purified by silica gel column chromatography using hexane/EtOAc (100:0 to 95:5) as eluent. Compound **S6** (1.21 g, 80%) was obtained as a clear oil.

Rf: 0.56 (hexane/EtOAc, 95:5); ^{1}H NMR (500 MHz, $CDCl_3$-d_1) δ 7.42-7.33 (m, 5H), 4.63 (s, 2H), 3.67-3.50 (m, 9H), 1.69-1.61 (m, 4H), 1.40-1.33 (m, 42H), 0.97 (t, *J*= 6.8 Hz, 3H), 0.92 (brs, 1H); ^{13}C NMR (126 MHz, $CDCl_3$-d_1) δ 138.6, 128.4, 127.7, 127.6, 78.1, 73.5, 71.8, 70.9, 70.7, 70.4, 32.1, 31.8, 30.3 29.9, 29.8, 29.7, 29.6, 29.5, 29.3, 22.9, 22.0-21.8 (m), 14.3, 13.7-13.1 (m); ^{2}H NMR (100 MHz, $CDCl_3$-d_1) δ 1.36 (brs, 2D), 0.86 (*J*= 2.0 Hz, 2D); ESI-MS: 579.6 $[M+H]^+$ and 601.6 $[M+Na]^+$; HRMS: 601.5468 calcd for $[C_{38}H_{66}D_4O_3Na]^+$ 601.5460 found.

3-((tetradecyl-13,13,14,14-*d*4)oxy)-2-(tetradecyloxy)propan-1-ol (**S7**)

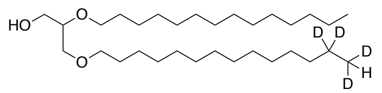

Compound **S6** (1.21 g, 2.09 mmol) was dissolved in a degassed mixture of EtOH/THF (1:1, 60 mL) and 20% $Pd(OH)_2$ (120 mg, 10% w/w) was added. The reaction was stirred under hydrogen atmosphere at room temperature for 4 hours. The catalyst was removed by filtration through a pad of celite, and the resulting residue was purified by column chromatography on silica gel using hexane/EtOAc (9:1 to 8:2) as the eluent. Alcohol **S7** (0.89 g, 87%) was obtained as a white solid.

Rf: 0.57 (hexane/EtOAc, 8:2); ^{1}H NMR (500 MHz, $CDCl_3$-d_1) δ 3.66-3.36 (m, 9H), 2.45 (brs, 1H), 1.53-1.47 (m, 4H), 1.25-1.09 (m, 42H), 0.82 (t, *J*= 6.8 Hz, 3H), 0.77 (brs, 1H); ^{13}C NMR (126 MHz, $CDCl_3$-d_1) δ ; 78.6, 71.9, 71.0, 70.5, 63.0, 32.1, 31.8, 30.2, 29.8, 29.8, 29.6, 29.5, 29.5, 26.3, 22.1-21.5 (m), 22.8, 14.2, 13.7-13.1 (m); ^{2}H NMR (100 MHz, $CDCl_3$-d_1) δ 1.20 (brs, 2D), 0.80 (*J*= 2.0 Hz, 2D); ESI-MS:

489.6 [M+H]$^+$ and 511.6 [M+Na]$^+$; HRMS: 511.4999 calcd for $[C_{31}H_{60}D_4O_3Na]^+$ 511.4994 found.

dibenzyl (3-((tetradecyl-13,13,14,14-*d*4)oxy)-2-(tetradecyloxy)propyl) phosphate (**S8**)

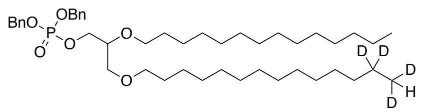

A solution of **S7** (572 mg, 1.17 mmol) in degassed DCM (27 mL) and tetrazole (0.45 M in acetonitrile, 10.4 mL) was prepared. Then, a solution of dibenzyl N,N-diisopropylphosphor-amidite (807 mg, 2.34 mmol) in degassed DCM (27 mL) was added dropwise and the reaction mixture was stirred at room temperature for 16 h. After the reaction was cooled to -40°C, a tert-Butyl hydroperoxide solution (5-6 M in decane, 2.2 mL) was added and the reaction mixture was allowed to warm up to rt over 2 h. The solvent was evaporated over vacuum and the resulting residue was purified over silica using hexane/EtOAc (9:1 to 8:2) as the eluent. Compound **S8** was obtained as a clear oil (687 mg, 78%).

Rf: 0.30 (hexane/EtOAc, 8:2); ^{1}H NMR (500 MHz, $CDCl_3$-d_1) *δ* 7.33-7.30 (m, 10H), 5.05-5.02 (m, 4H), 4.13-3.98 (m, 2H), 3.56-3.36 (m, 7H), 1.54-1.47 (m, 4H), 1.28-1.20 (m, 42H), 0.86 (t, *J*= 6.8 Hz, 3H), 0.81 (brs, 1H); ^{13}C NMR (126 MHz, $CDCl_3$-d_1) *δ* 136.0, 135.9, 128.6, 128.5, 128.0, 77.3, 77.2, 71.9, 70.8, 69.8, 69.3, 69.3, 67.2, 67.1, 32.1, 31.8, 30.1, 29.8, 29.8, 29.6, 29.5, 29.5, 26.2, 26.2, 22.8, 22.1-21.5 (m), 14.3, 13.7-13.1 (m); ^{31}P NMR (202 MHz, $CDCl_3$-d_1) *δ* -0.07; ^{2}H NMR (100 MHz, $CDCl_3$-d_1) *δ* 1.24 (brs, 2D), 0.84 (*J*= 2.0 Hz, 2D); ESI-MS: 749.6 [M+H]$^+$ and 771.6 [M+Na]$^+$; HRMS: 749.5782 calcd for $[C_{45}H_{74}D_4O_6P]^+$ 749.5781 found.

3-((tetradecyl-13,13,14,14-*d*4)oxy)-2-(tetradecyloxy)propyl hydrogen phosphate (**13**)

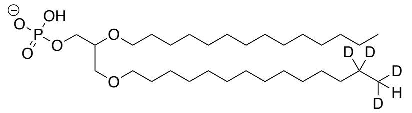

Protected lipid **S8** (522 mg, 0.70 mmol) was dissolved in a degassed mixture of EtOH/THF (2:1, 45 mL) and Pd/C (52 mg, 10% w/w) was added. The reaction was stirred under hydrogen atmosphere at room temperature for 4 h. The catalyst was removed by filtration through a pad of celite, and the resulting residue was purified on Sephadex LH 20 (DCM/MeOH 7:3). Lipid **13** (350 mg, 88%) was obtained as a white solid.

^{1}H NMR (500 MHz, $CDCl_3$-d_1) *δ* 4.06-3.98 (m, 2H), 3,64-3,43 (m, 7H), 1.56-1.48 (m, 4H), 1.39-1.11 (M, 42H), 0.84 (t, *J*= 6.8 Hz, 3H), 0.79 (brs, 1H); ^{13}C NMR (126 MHz, $CDCl_3$-d_1) *δ* 77.8, 72.2, 71.2, 70.3, 66.2, 32.1, 31.9, 30.0, 29.9, 29.8, 29.7, 29.6, 29.6, 26.2, 26.1, 22.9, 22.2-21.6 (m), 14.3, 13.7-13.1 (m); ^{31}P NMR (202 MHz, $CDCl_3$-d_1) *δ* 1.43; ^{2}H NMR (100 MHz, $CDCl_3$-d_1) *δ* 1.24 (brs, 2D), 0.83 (*J*= 2.0 Hz, 2D); ESI-MS: 567.6 [M-H]$^-$; HRMS: 567.4697 calcd for $[C_{31}H_{60}D_4O_6P]^-$ 567.4702 found.

3-(benzyloxy)propane-1,2-diol (**S9**)

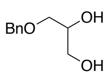

A solution of 1,3-O-benzylideneglycerol (1.50 g, 8.3 mmol) in dry DCM (34 mL) was cooled to -78°C. Then, DIBAL-H (33.0 mmol) in DCM (1M) was added dropwise to the cold solution and reacted for 16 h at rt. After the reaction was complete, a small amount of MeOH was used to carefully quench the remaining DIBAL-H followed by the addition of aqueous NaOH (5 M, 40 mL). The solution was then extracted using diethyl ether (Et_2O) (3x100 mL) and then washed with water (1x100 mL). The extracted organic layer was dried using Na_2SO_4 and the solvent was removed under vacuum. The crude was purified over silica using EtOAc as the eluent and alcohol **S9** (1.18 g, 78%) was obtained as a clear oil.

Rf: 0.43 (EtOAc); ^{1}H NMR spectrum matched previously reported data.[6]

((2,3-bis(tetradec-13-yn-1-yloxy)propoxy)methyl)benzene(**S10**)

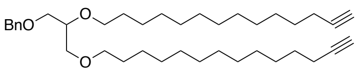

A suspension of KOH (0.59 g, 10.5 mmol) in dry DMSO (20 mL) was stirred at room temperature for 30 minutes. The mixture was cooled with ice water and a solution of **S9** (0.38 g, 2.1 mmol) and **S2** (1.8 g, 6.2 mmol) in dry DMSO (20 mL) was added. The mixture was then stirred at 40 °C for 3 days. Water (400 mL) was added and the mixture was extracted with EtOAc (5x75 mL). The combined organic layers were washed with water (2x100 mL), brine (100 mL) and dried over Na_2SO_4. Purification by silica gel column chromatography using hexane/EtOAc (95:5) as the eluent yielded **S10** (1.52 g, 84%) as a colorless oil.

Rf: 0.42 (hexane/EtOAc, 95:5); ^{1}H NMR (500 MHz, $CDCl_3$-d$_1$) δ 7.39-7.32 (m, 5H), 4.61 (s, 2H), 3.66-3.46 (m, 9H), 2.22 (ddd, *J*= 7.3, 7.0, 2.6 Hz, 4H), 2.22 (t, *J*= 2.6 Hz, 2H),1.63-1.54 (m, 8H), 1.45-1.29 (m, 32H); ^{13}C NMR (126 MHz, $CDCl_3$-d$_1$) δ 138.6, 128.4, 127.7, 127.6, 84.8, 78.1, 73.4, 71.8, 70.9, 70.7, 70.4, 68.2, 30.2, 29.8, 29.7, 29.6, 29.3, 28.9, 28.6, 28.6, 26.2, 18.5; ESI-MS: 567.2 $[M+H]^+$ and 584.2 $[M+NH_4]^+$; HRMS: 589.4591 calcd for $[C_{38}H_{62}O_3Na]^+$ 589.4593 found.

2-((benzyloxy)methyl)-1,4-dioxacyclodotriaconta-17,19-diyne (**S11**)

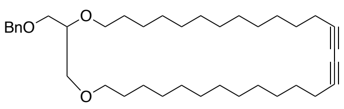

To a refluxing solution of Copper(II) acetate monohydrate (0.89 g, 4.40 mmol) in dry pyridine (60 mL), a solution of **S10** (0.5 g, 0.88 mmol) in dry pyridine (10 mL), was added over 4 hours. After an hour at 80 °C, the reaction mixture was cooled to room temperature, and acidified to pH 2 using a 1 M HCl (aq) solution. The mixture was extracted with EtOAc (3x100 mL) and combined organic layers were washed with 1% HCl solution (50 mL), water (50 mL), brine (50 mL) and dried over Na_2SO_4. Diyne **S11** (0.10 g, 20%) was obtained as a yellow oil after purification by silica gel column chromatography using hexane/EtOAc (98:2 to 90:10) as eluent.

Rf: 0.31 (hexane/EtOAc, 95:5); ^{1}H NMR (500 MHz, $CDCl_3$-d_1) δ 7.38-7.30 (m, 5H), 4.60 (s, 2H), 3.68-3.47 (m, 9H), 2.22 (t, J= 7.0 Hz, 4H), 1.63-1.28 (m, 40H); ^{13}C NMR (126 MHz, $CDCl_3$-d_1) δ 138.6, 128.5, 127.8, 127.7, 78.2, 77.7, 73.6, 71.8, 71.3, 70.8, 70.5, 65.7, 30.2, 29.9, 29.5, 29.4, 29.2, 28.7, 28.6, 28.2, 26.2, 26.1, 19.4; ESI-MS: 565.4 $[M+H]^+$ and 582.3 $[M+NH_4]^+$; HRMS: 587.4435 calcd for $[C_{38}H_{60}O_3Na]^+$ 587.4437 found.

2-((benzyloxy)methyl)-1,4-dioxacyclodotriacontane-17,17,18,18,19,19,20,20-*d*8 (**S12**)

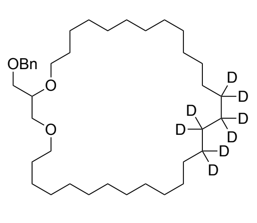

Alkyne **S11** (172 mg, 0.31 mmol) was dissolved in degassed THF (4 mL) and Wilkinson catalyst (50 mg, 0.06 mmol) was added. The reaction was stirred under deuterium atmosphere at 35 °C for 40 h. Solvent was evaporated and the resulting residue was purified by silica gel column chromatography using hexane/EtOAc (98:2 to 95:5) as eluent. Compound **S12** (170 mg, 94%) was obtained as a clear oil.

Rf: 0.36 (hexane/EtOAc, 95:5); ^{1}H NMR (500 MHz, $CDCl_3$-d_1) δ 7.33-7.24 (m, 5H), 4.54 (s, 2H), 3.62-3.43 (m, 9H), 1.57-1.53 (m, 4H), 1.34-1.23 (m, 40H); ^{13}C NMR (126 MHz, $CDCl_3$-d_1) δ 138.6, 128.5, 127.7, 127.7, 78.2, 73.6, 71.8, 71.3, 70.8, 70.4, 30.2, 29.8, 29.6, 29.5, 29.5, 29.4, 29.3, 29.1, 28.8, 28.1-27.4 (m), 26.3, 26.2; ^{2}H NMR (100 MHz, $CDCl_3$-d_1) δ 1.22 (brs, 8D); ESI-MS: 581.4 $[M+H]^+$ and 603.6 $[M+Na]^+$; HRMS: 603.5563 calcd for $[C_{38}H_{60}D_8O_3Na]^+$ 603.5564 found.

(1,4-dioxacyclodotriacontan-2-yl-17,17,18,18,19,19,20,20-*d*8)methanol (**S13**)

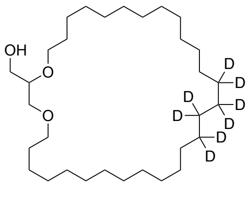

Compound **S12** (168 mg, 0.29 mmol) was dissolved in a degassed mixture of EtOH/THF (1:1, 60 mL) and 20% $Pd(OH)_2$ (17 mg, 10% w/w) was added. The reaction was stirred under hydrogen atmosphere at room temperature for 4 hours. The catalyst was removed by filtration through a pad of celite, and the resulting residue was purified by column chromatography on silica gel using hexane/EtOAc (9:1 to 8:2) as the eluent. Alcohol **S13** (128 mg, 90%) was obtained as a white gum and used without further characterization.

(1,4-dioxacyclodotriacontan-2-yl-17,17,18,18,19,19,20,20-*d*8)methyl dibenzyl phosphate (**S14**)

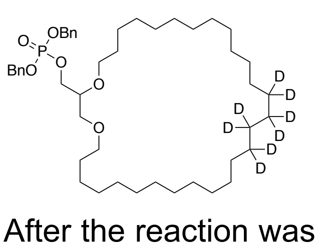

A solution of **S13** (128 mg, 0.26 mmol) in degassed DCM (6 mL) and tetrazole (0.45 M in acetonitrile, 2.3 mL) was prepared. Then, a solution of dibenzyl N,N-diisopropylphosphoramidite (180 mg, 0.52 mmol) in degassed DCM (6 mL) was added dropwise and the reaction mixture was stirred at room temperature for 16 h. After the reaction was cooled to -40°C, a tert-Butyl hydroperoxide solution (5-6 M

in decane, 0.4 mL) was added and the reaction mixture was allowed to warm up to rt over 2 h. The solvent was evaporated over vacuum and the resulting residue was purified over silica using hexane/EtOAc (9:1 to 8:2) as the eluent. Compound **S14** was obtained as a clear oil (155 mg, 79%).

Rf: 0.13 (hexane/EtOAc, 8:2); ^{1}H NMR (500 MHz, $CDCl_3$-d_1) *δ* 7.44-7.33 (m, 10H), 5.14-5.08 (m; 4H), 4.19-4.07 (m, 2H), 3.64-3.44 (m, 7H), 1.61-1.56 (m, 4H), 1.34-1.30 (m, 40H); ^{13}C NMR (126 MHz, $CDCl_3$-d_1) *δ* 136.0, 136.0, 128.7, 128.6, 128.0, 77.4, 77.3, 71.8, 70.7, 70.2, 69.4, 69.3, 67.1, 67.0, 30.0, 29.7, 29.6, 29.5, 29.4, 29.4, 29.3, 29.3, 29.2, 29.0, 28.7, 28.7, 28.1-27.3 (m), 26.1, 26.1; ^{31}P NMR (202 MHz, $CDCl_3$-d_1) *δ* -0.27; ^{2}H NMR (100 MHz, $CDCl_3$-d_1) *δ* 1.21 (brs, 8D); ESI-MS: 751.5 $[M+H]^+$ and 773.6 $[M+Na]^+$; HRMS: 751.5876 calcd for $[C_{45}H_{68}D_8O_6P]^+$ 751.5875 found.

(1,4-dioxacyclodotriacontan-2-yl-17,17,18,18,19,19,20,20-*d*8)methyl hydrogen phosphate (**14**)

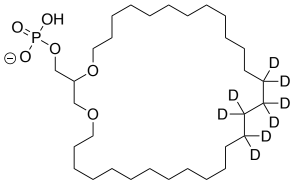

Protected lipid **S14** (155 mg, 0.21 mmol) was dissolved in a degassed mixture of EtOH/THF (2:1, 18 mL) and Pd/C (15 mg, 10% w/w) was added. The reaction was stirred under hydrogen atmosphere at room temperature for 4 h. The catalyst was removed by filtration through a pad of celite, and the resulting residue was purified on Sephadex LH 20 (DCM/MeOH 7:3). Lipid **S14** (101 mg, 86%) was obtained as a white solid.

^{1}H NMR (500 MHz, $CDCl_3$-d_1) *δ* 8.90 (brs, 2H), 4.06-3.97 (m, 2H), 3.65-3.43 (m, 7H), 1.56-1.50 (m, 4H), 1.31-1.11 (m, 40H); ^{13}C NMR (126 MHz, $CDCl_3$-d_1) *δ* 77.9, 72.1, 71.1, 70.5, 66.1, 50.4, 30.0, 29.8, 29.7, 29.6, 29.5, 29.5, 29.4, 29.3, 29.2, 29.1, 29.0, 28.7, 28.6, 27.8-27.3 (m), 26.2, 26.1; ^{31}P NMR (202 MHz, $CDCl_3$-d_1) *δ* 0.70; ^{2}H NMR (100 MHz, $CDCl_3$-d_1) *δ* 1.20 (brs, 8D); ESI-MS: 569.6 $[M-H]^-$; HRMS: 569.4792 calcd for $[C_{31}H_{54}D_8O_6P]^-$ 569.4792 found.

4. References

(1) Leriche, G.; Cifelli, J. L.; Sibucao, K. C.; Patterson, J. P.; Koyanagi, T.; Gianneschi, N. C.; Yang, J. *Org. Biomol. Chem.* **2017**, *15* (10), 2157–2162.

(2) Fujiwara, K.; Murata, Y.; Wan, T. S. M.; Komatsu, K. *Tetrahedron* **1998**, *54* (10), 2049–2058.

(3) Azagarsamy, M. A.; Yesilyurt, V.; Thayumanavan, S. *J. Am. Chem. Soc.* **2010**, *132* (13), 4550–4551.

(4) Febo-Ayala, W.; Morera-Félix, S. L.; Hrycyna, C. A.; Thompson, D. H. *Biochemistry* **2006**, *45* (49), 14683–14694.

(5) Nakamura, M.; Goto, R.; Tadokoro, T.; Shibakami, M. *J. Colloid Interface*

Sci. **2007**, *310* (2), 630–642.

(6) Zhang, Y. J.; Dayoub, W.; Chen, G. R.; Lemaire, M. *Green Chem.* **2011**, *13* (10), 2737–2742.

5. NMR spectra

Compound **2**

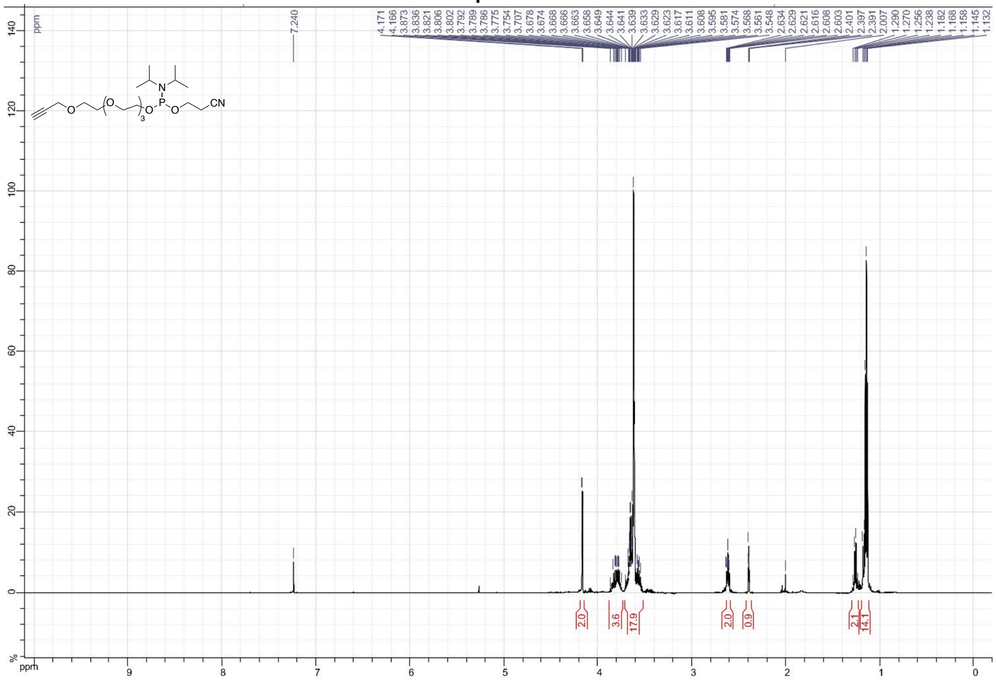

Figure S6. ^{1}H NMR Spectrum of compound **2**

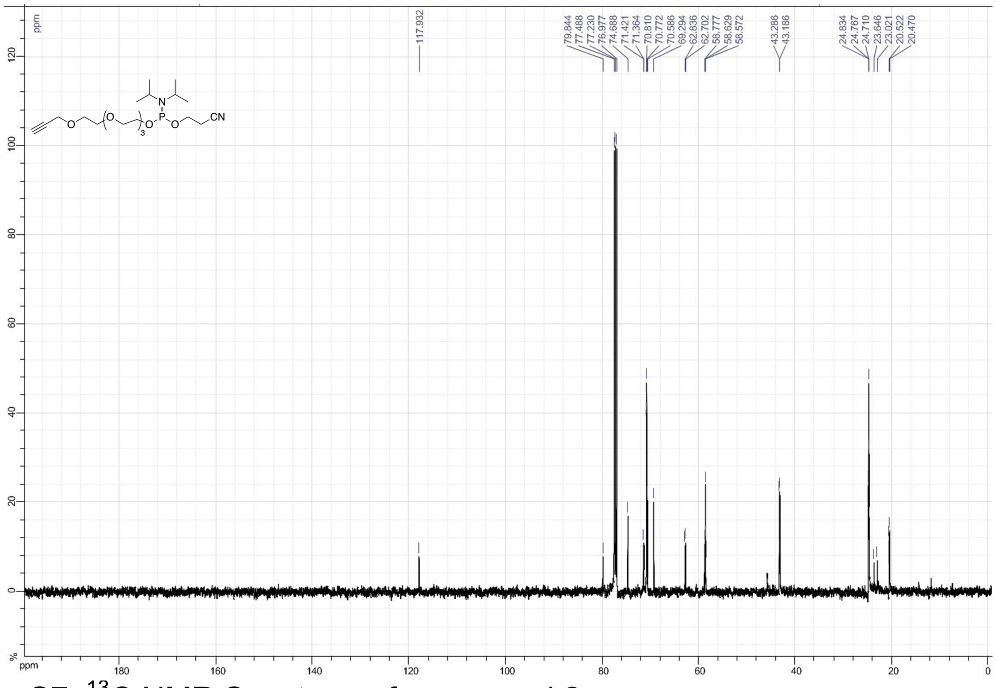

Figure S7. ^{13}C NMR Spectrum of compound **2**

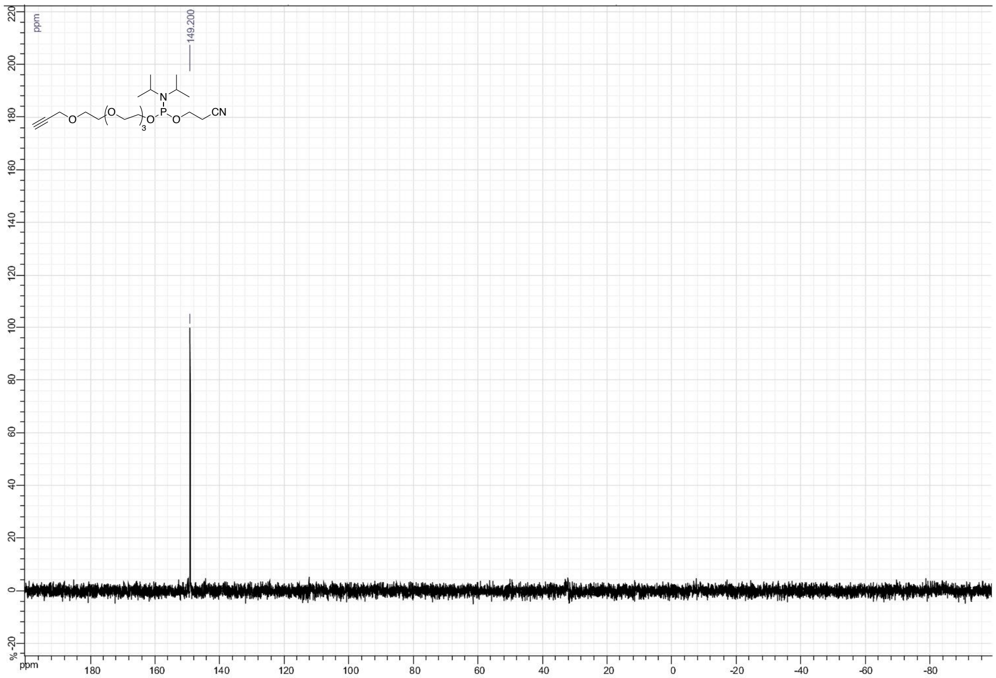

Figure S8. ^{31}P NMR Spectrum of compound **2**

Compound **3**

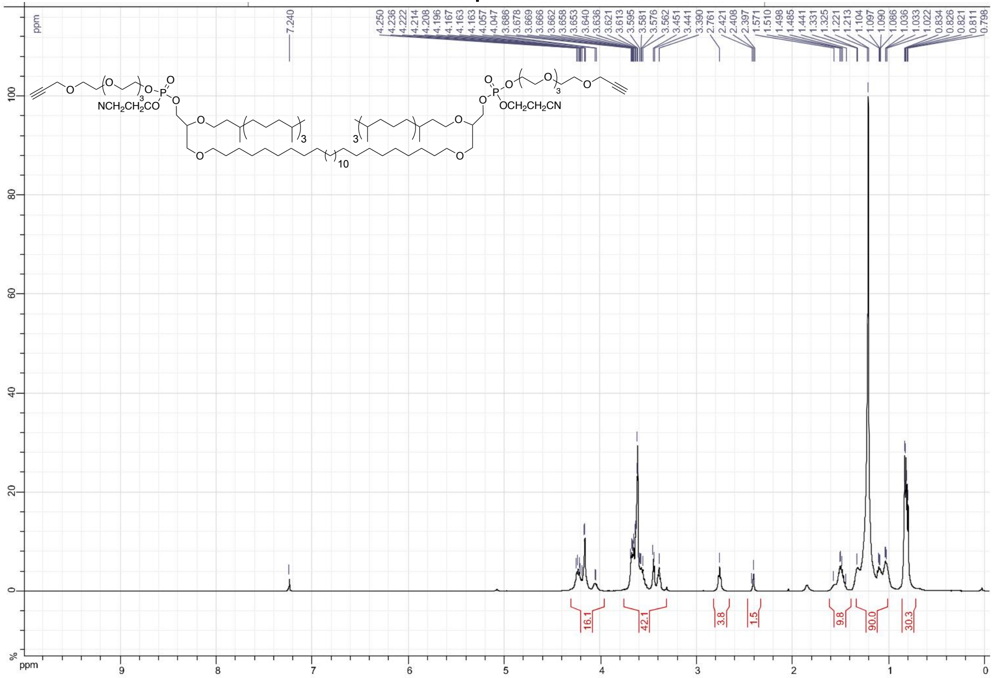

Figure S9. ^{1}H NMR Spectrum of compound **3**

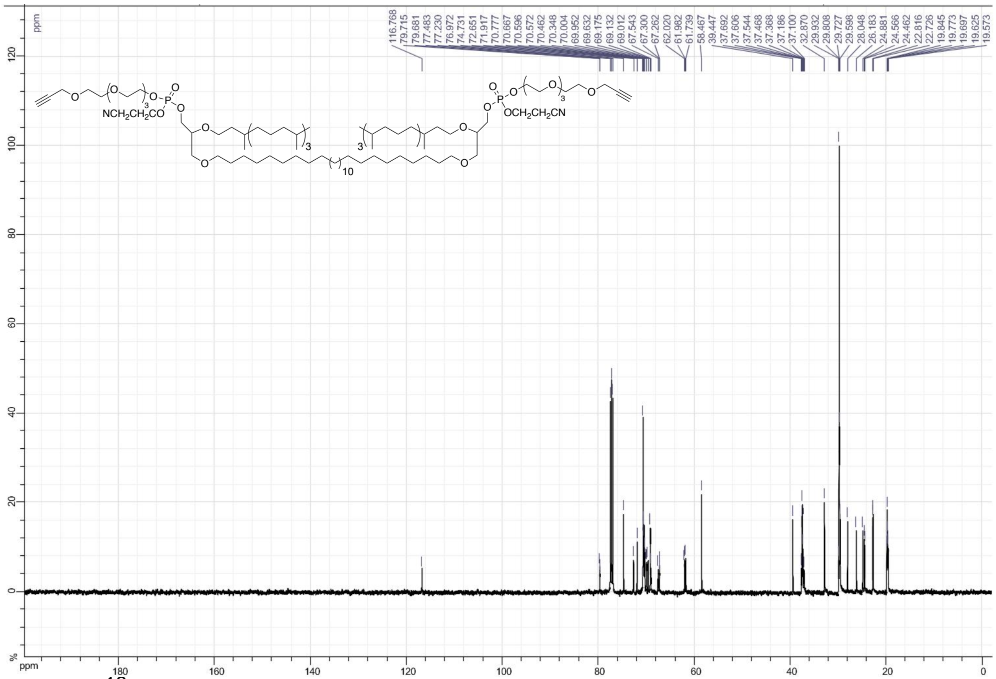

Figure S10. ^{13}C NMR Spectrum of compound **3**

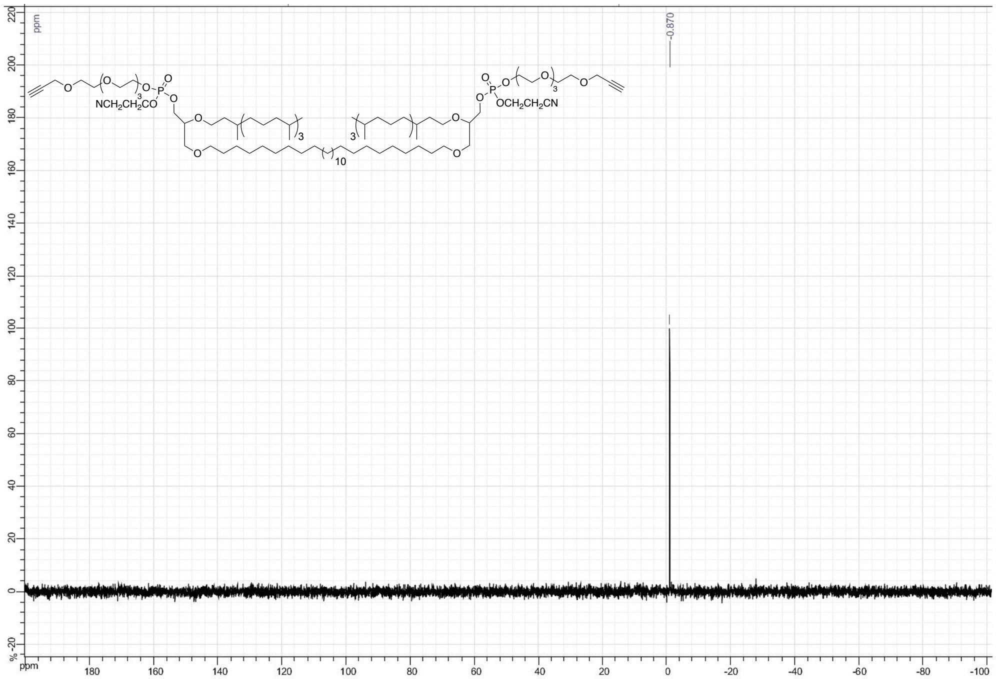

Figure S11. ^{31}P NMR Spectrum of compound **3**

NBD-N_3

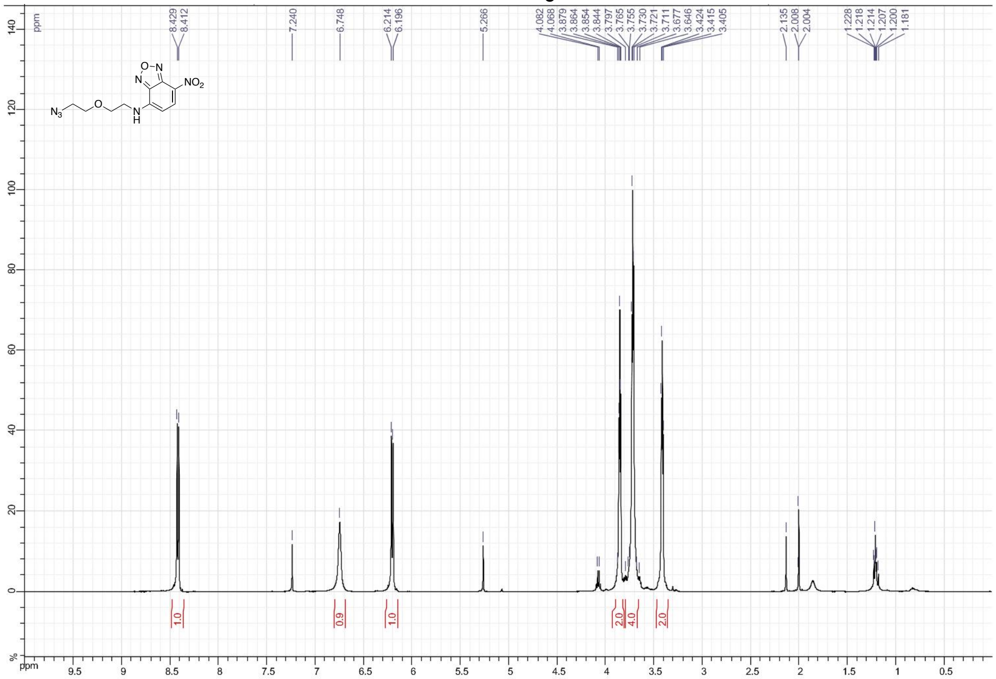

Figure S12. ^{1}H NMR Spectrum of **NBD-N_3**

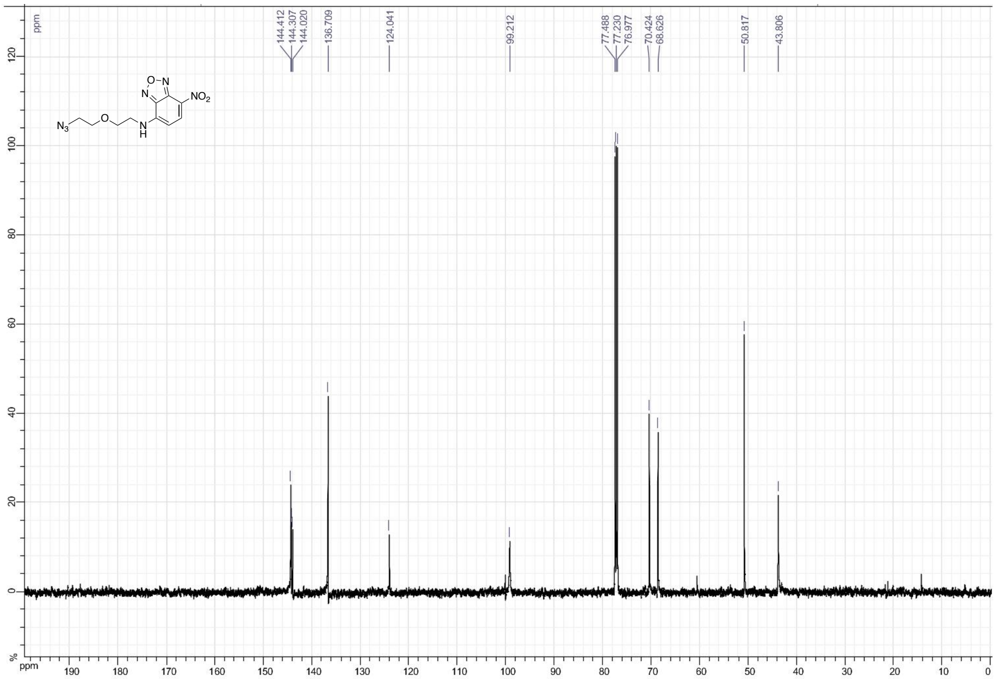

Figure S13. ^{13}C NMR Spectrum of **NBD-N_3**

Rho-N_3

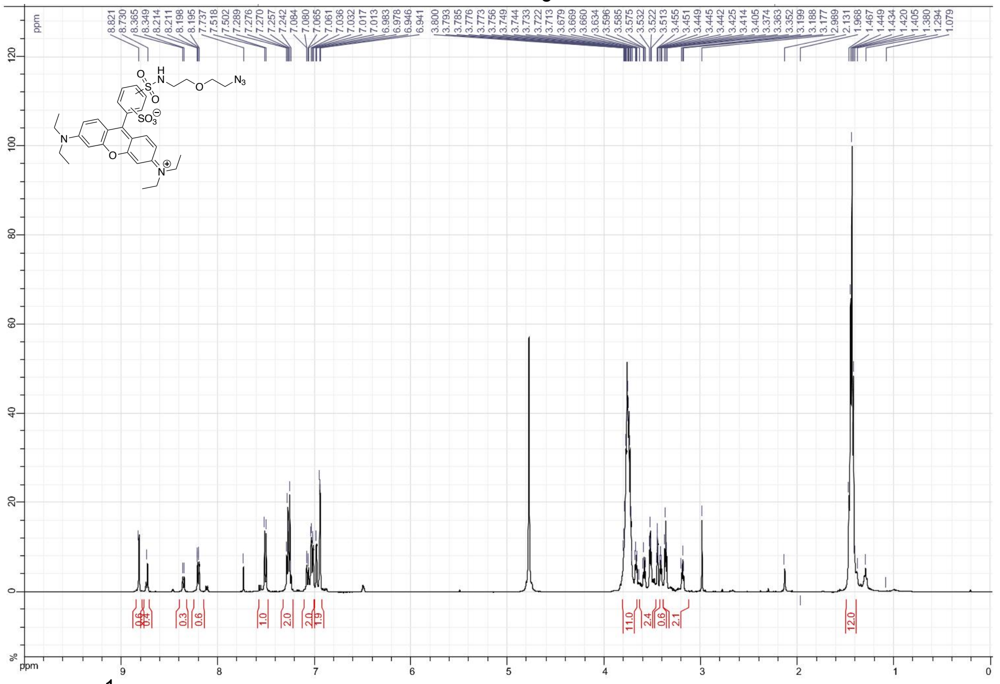

Figure S14. ^{1}H NMR Spectrum of **Rho-N_3**

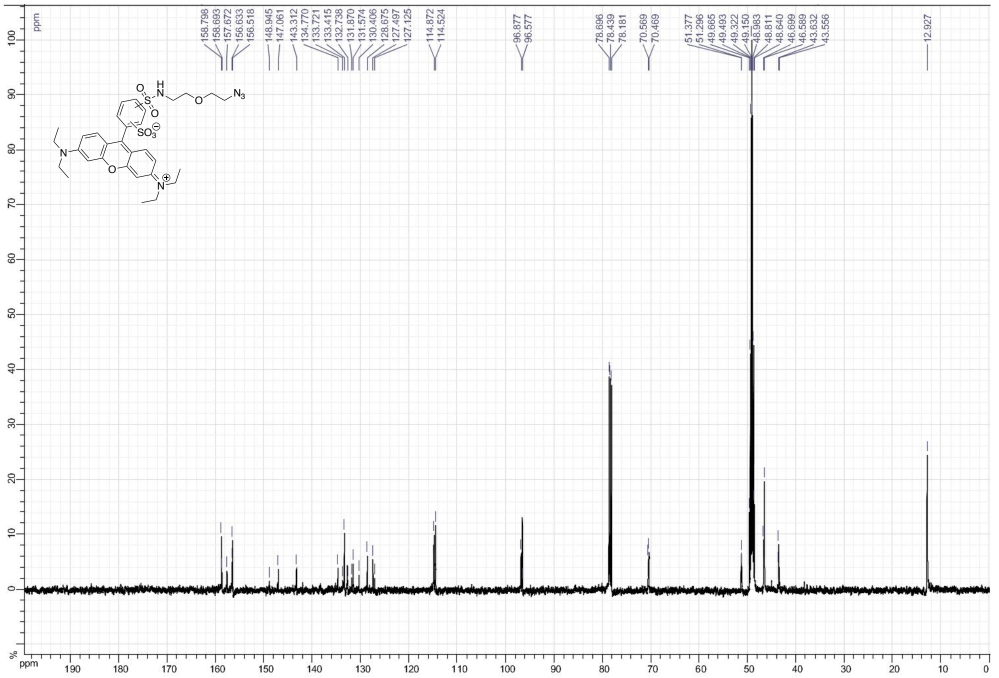

Figure S15. ^{13}C NMR Spectrum of **Rho-N_3**

Protected NBD lipid (**4**)

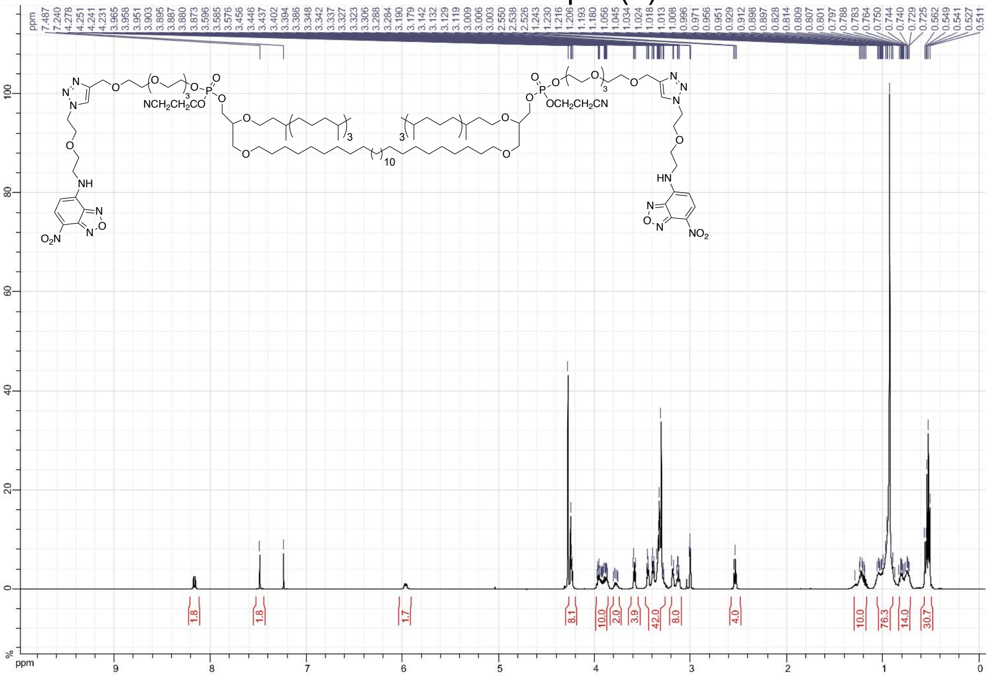

Figure S16. ^{1}H NMR Spectrum of **Protected NBD lipid (4)**

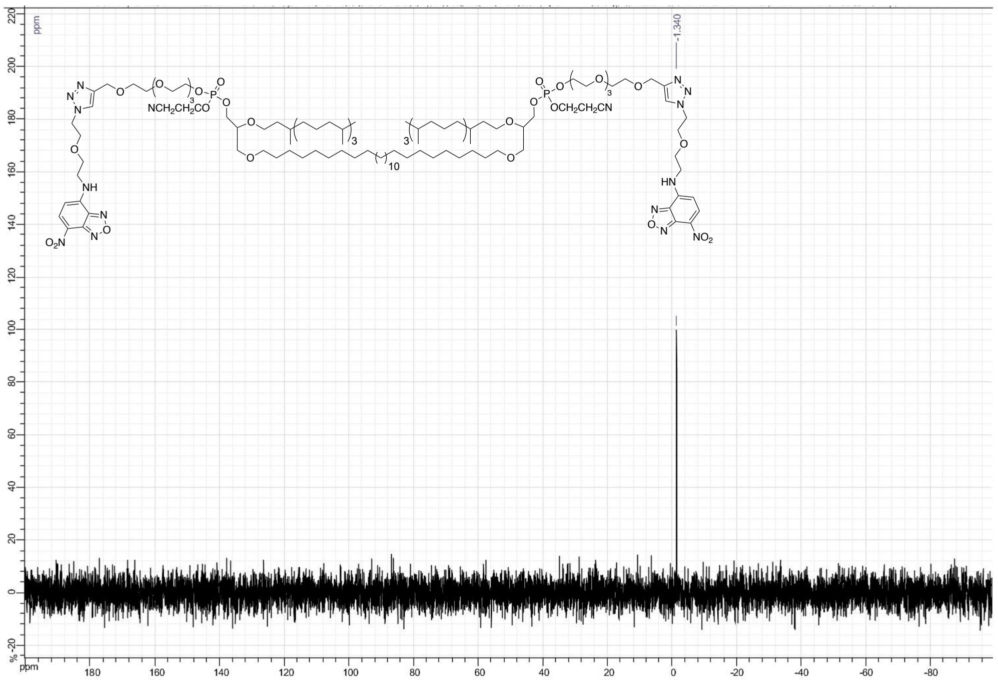

Figure S17. ^{31}P NMR Spectrum of **Protected NBD lipid (4)**

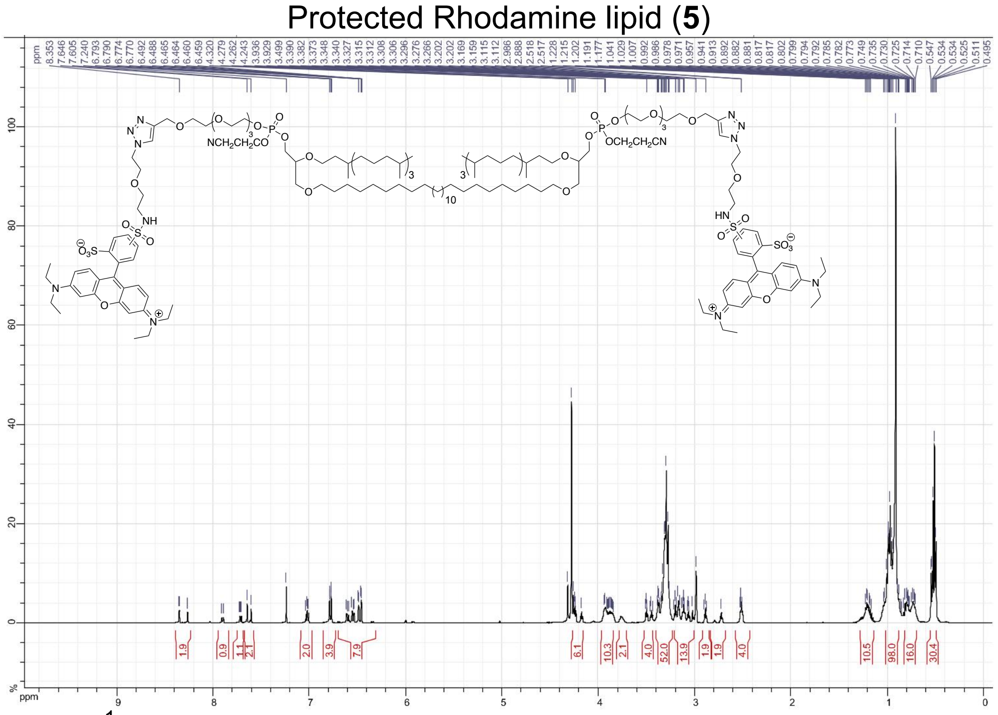

Figure S18. ^{1}H NMR Spectrum of **Protected Rhodamine lipid (5)**

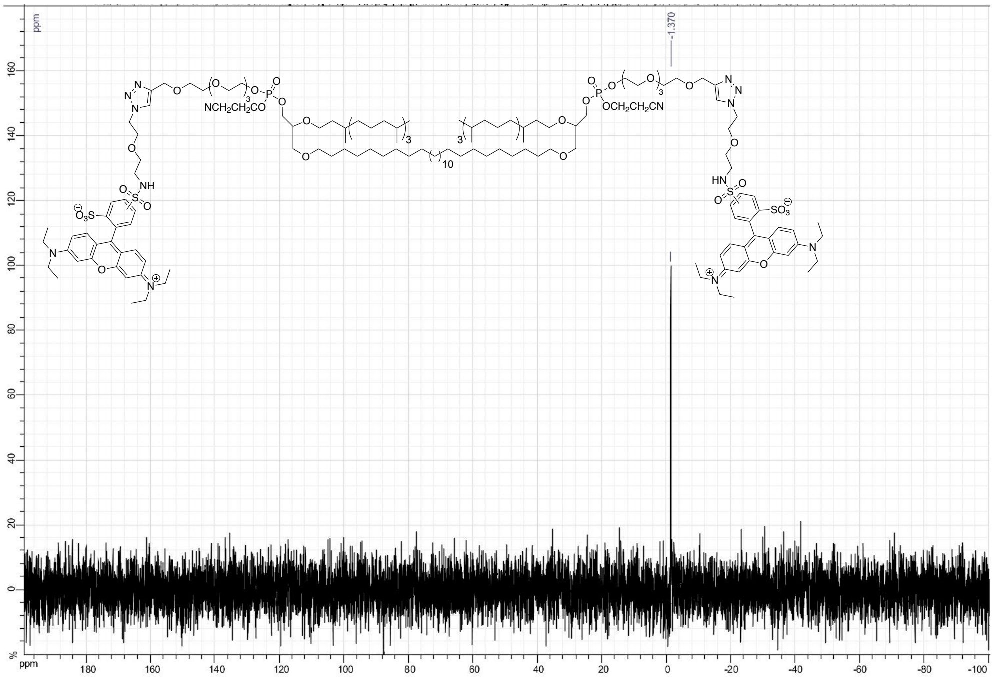

Figure S19. ^{31}P NMR Spectrum of **Protected Rhodamine lipid (4)**

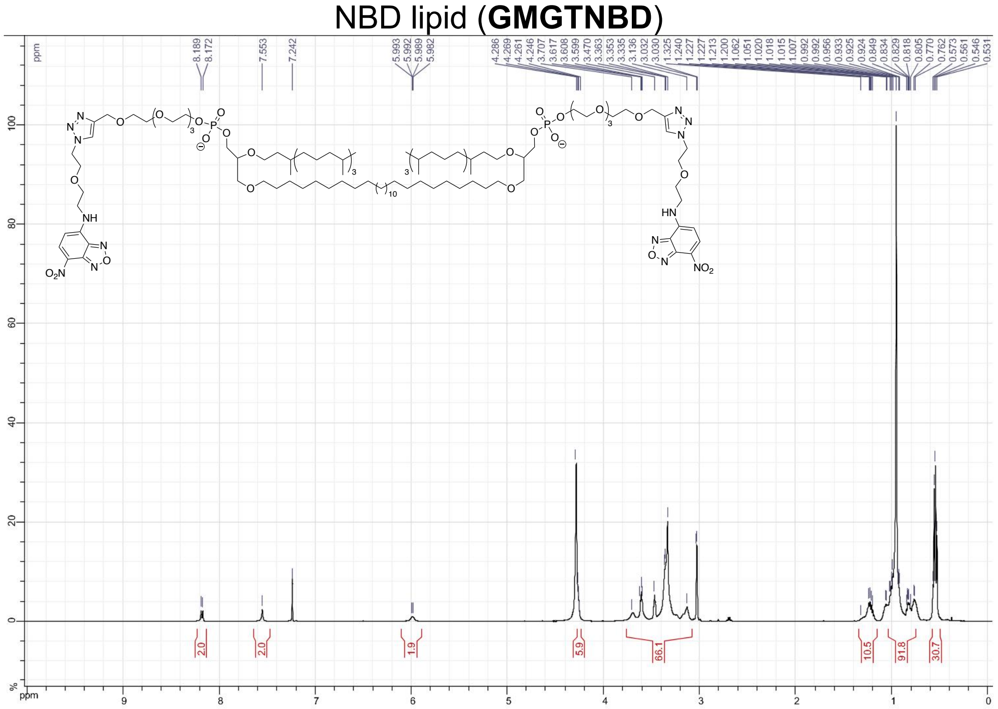

Figure S20. ^{1}H NMR Spectrum of **NBD lipid (GMGTNBD)**

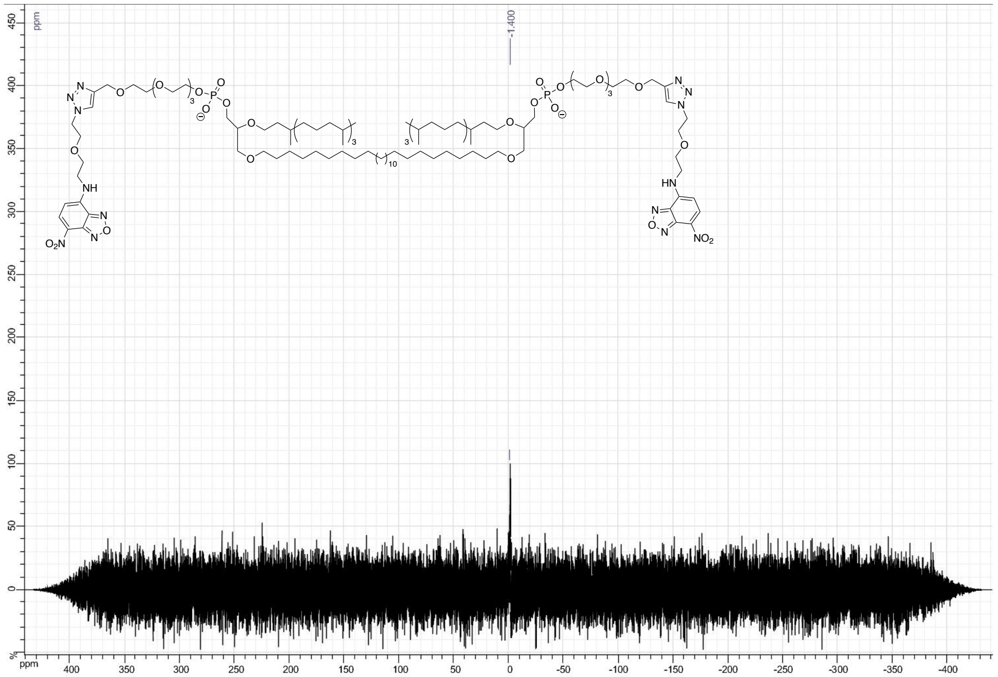

Figure S21. ^{31}P NMR Spectrum of **NBD lipid (GMGTNBD)**

Rhodamine lipid (**GMGTRho**)

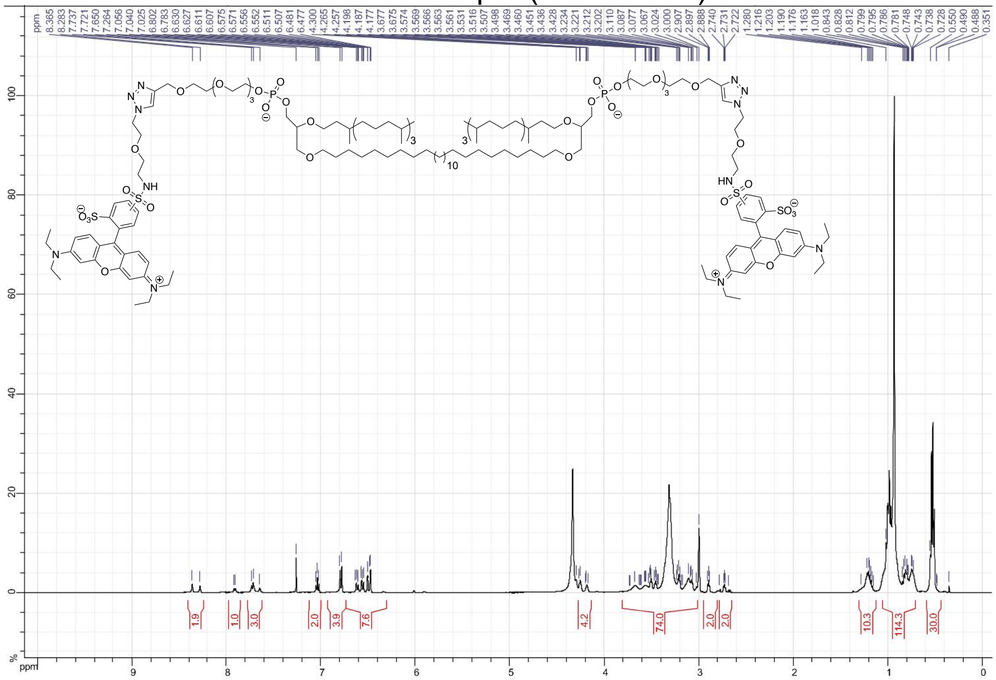

Figure S22. ^{1}H NMR Spectrum of **Rhodamine lipid (GMGTRho)**

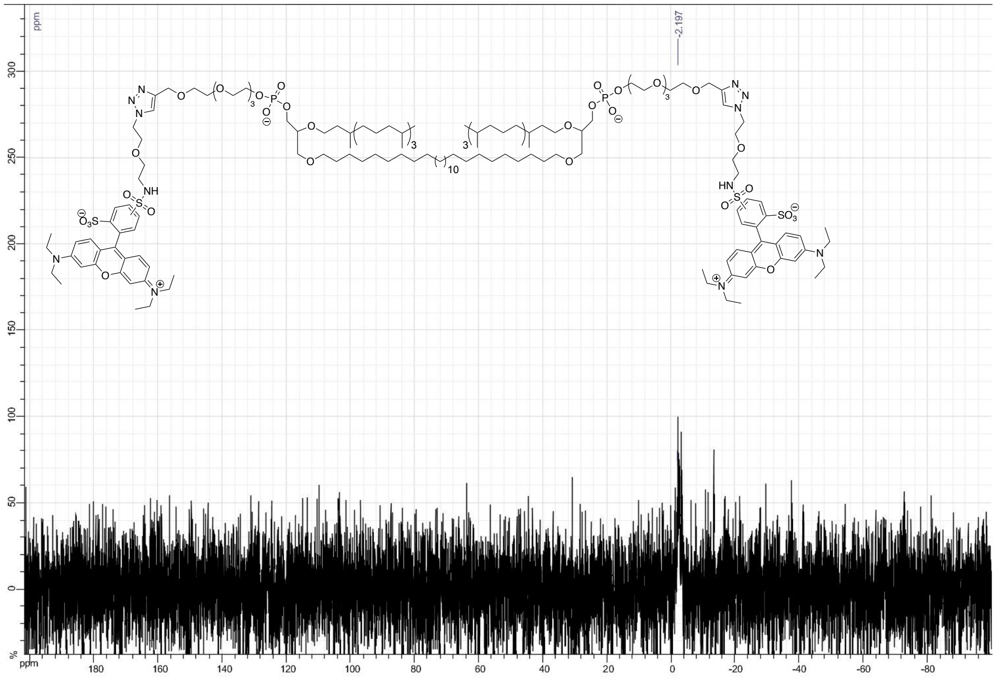

Figure S23. ^{31}P NMR Spectrum of **Rhodamine lipid (GMGTRho)**

Compound **7**

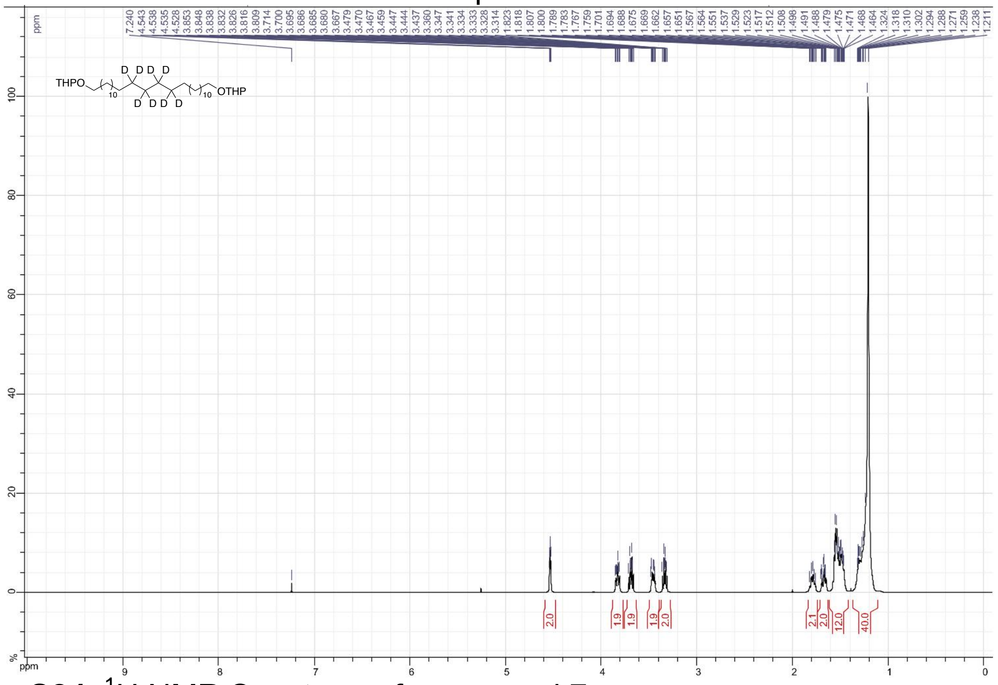

Figure S24. ^{1}H NMR Spectrum of compound **7**

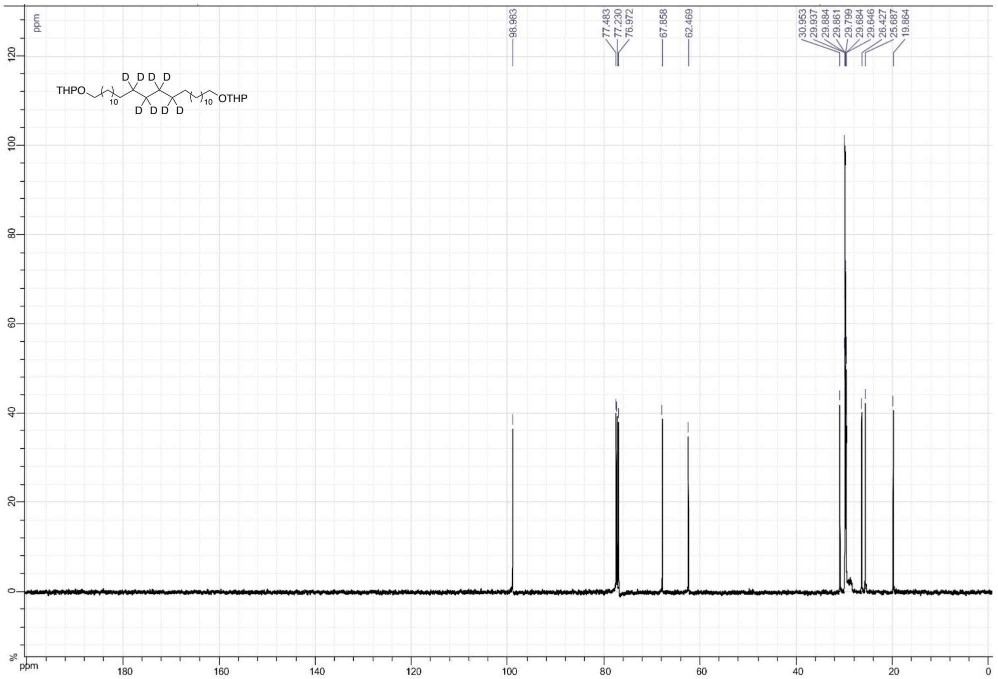

Figure S25. ^{13}C NMR Spectrum of compound **7**

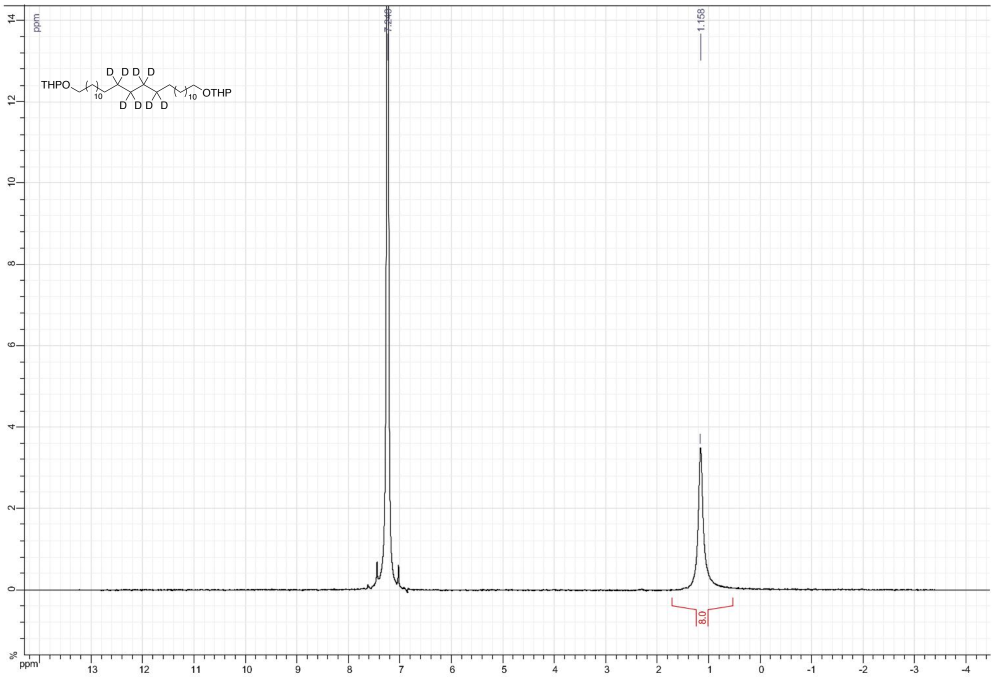

Figure S26. ^{2}H NMR Spectrum of compound **7**

Compound **8**

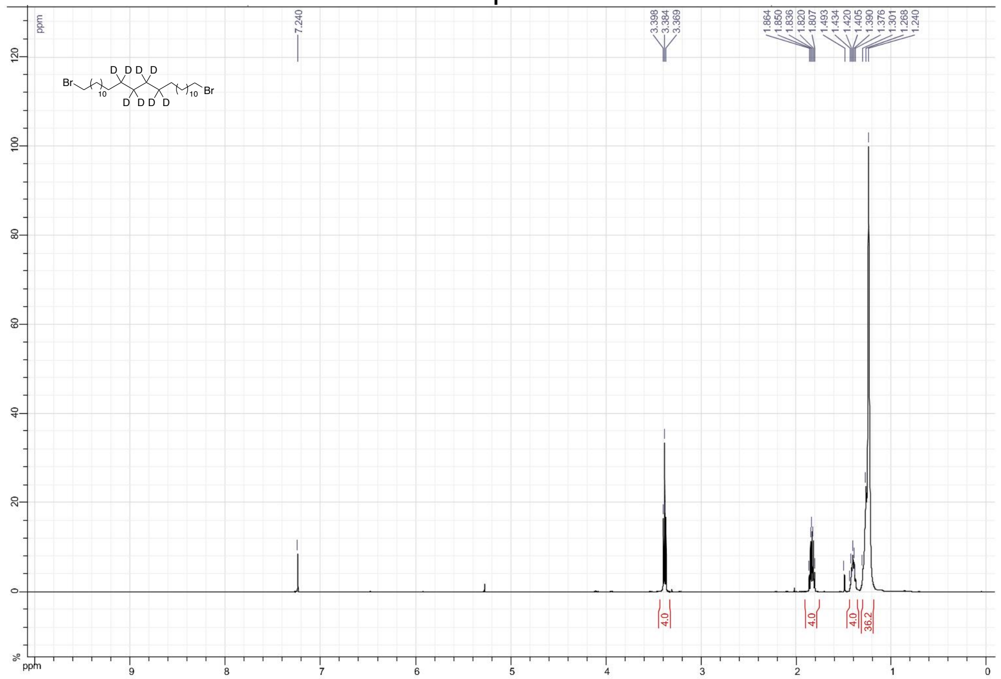

Figure S27. ^{1}H NMR Spectrum of compound **8**

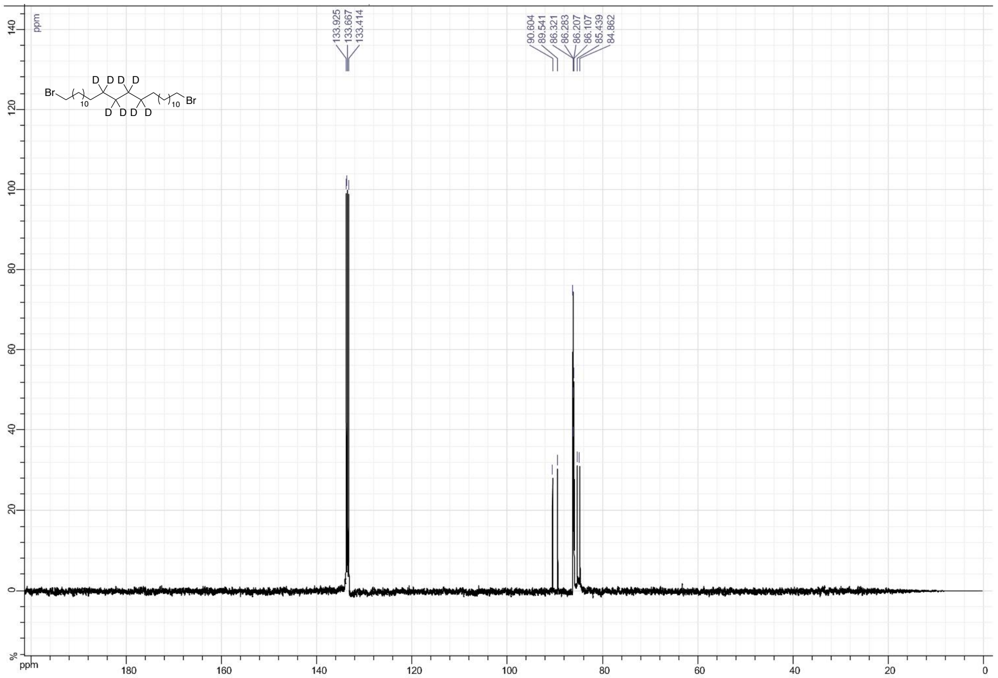

Figure S28. ^{13}C NMR Spectrum of compound **8**

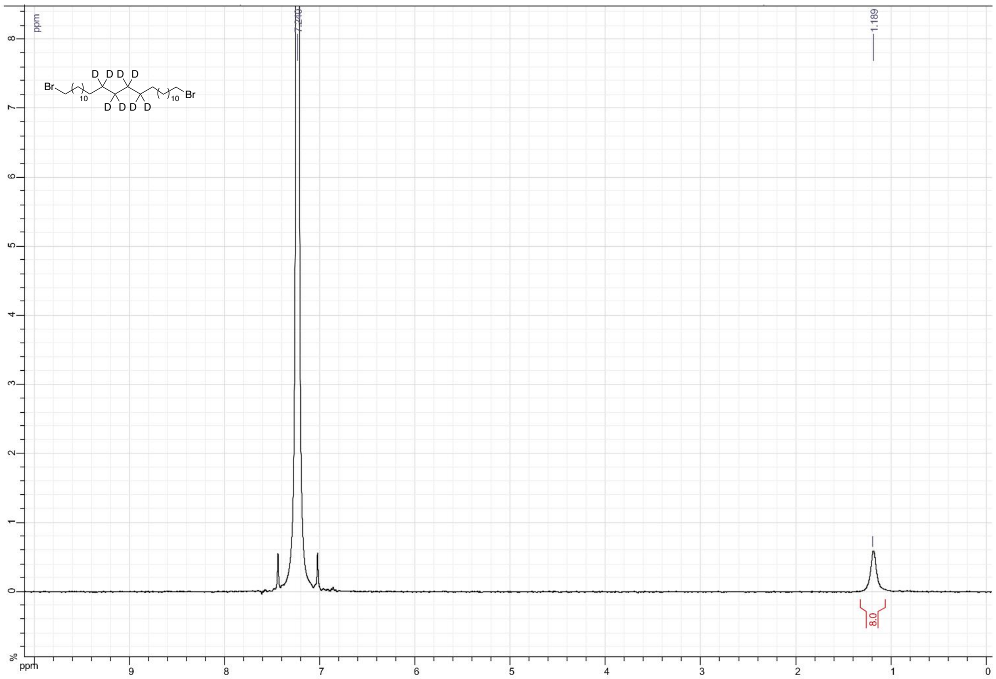

Figure S29. ^{2}H NMR Spectrum of compound **8**

Compound **10**

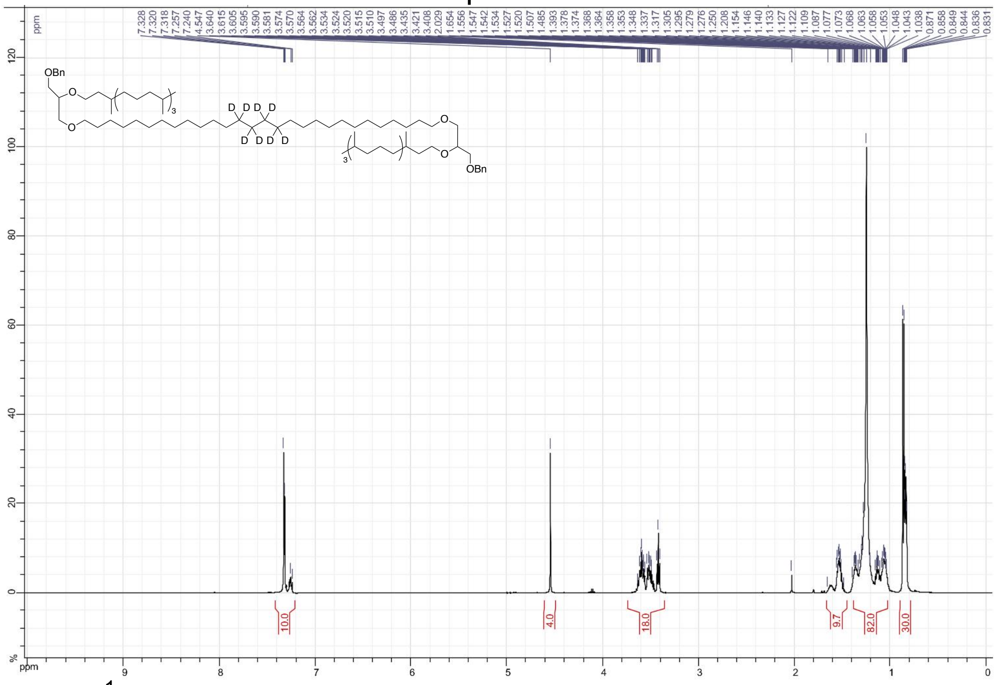

Figure S30. ^{1}H NMR Spectrum of compound **10**

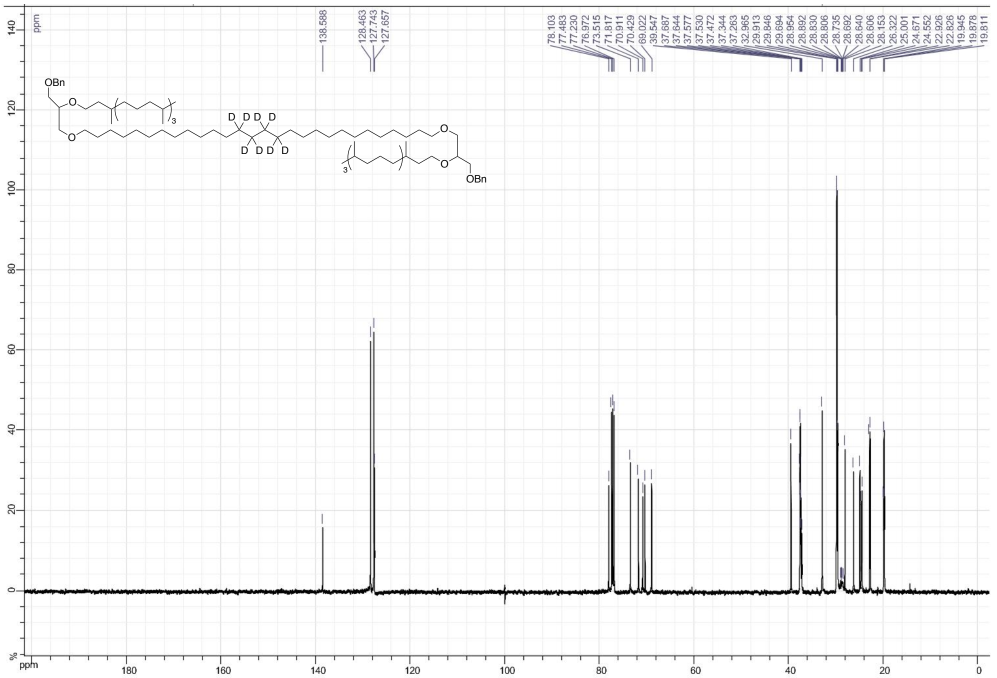

Figure S31. ^{13}C NMR Spectrum of compound **10**

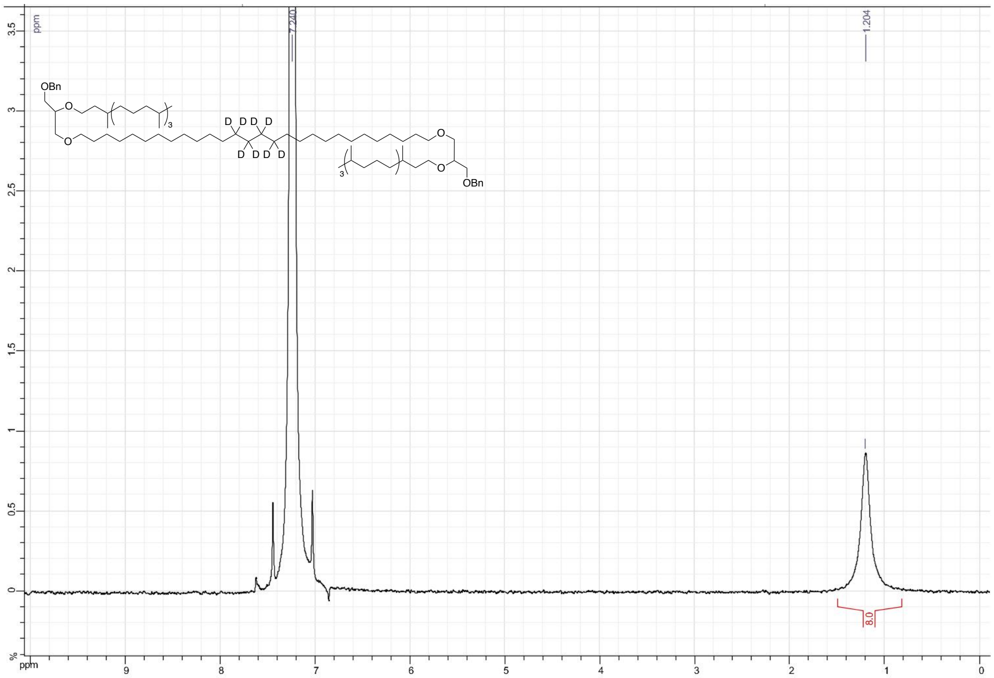

Figure S32. ^{2}H NMR Spectrum of compound **10**

Compound **11**

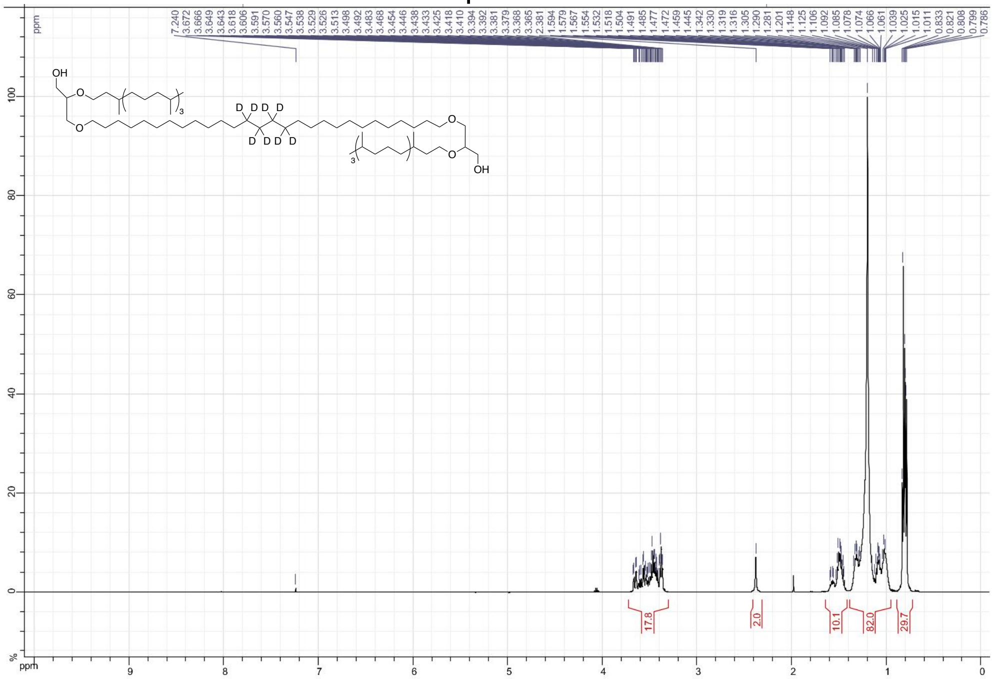

Figure S33. ^{1}H NMR Spectrum of compound **11**

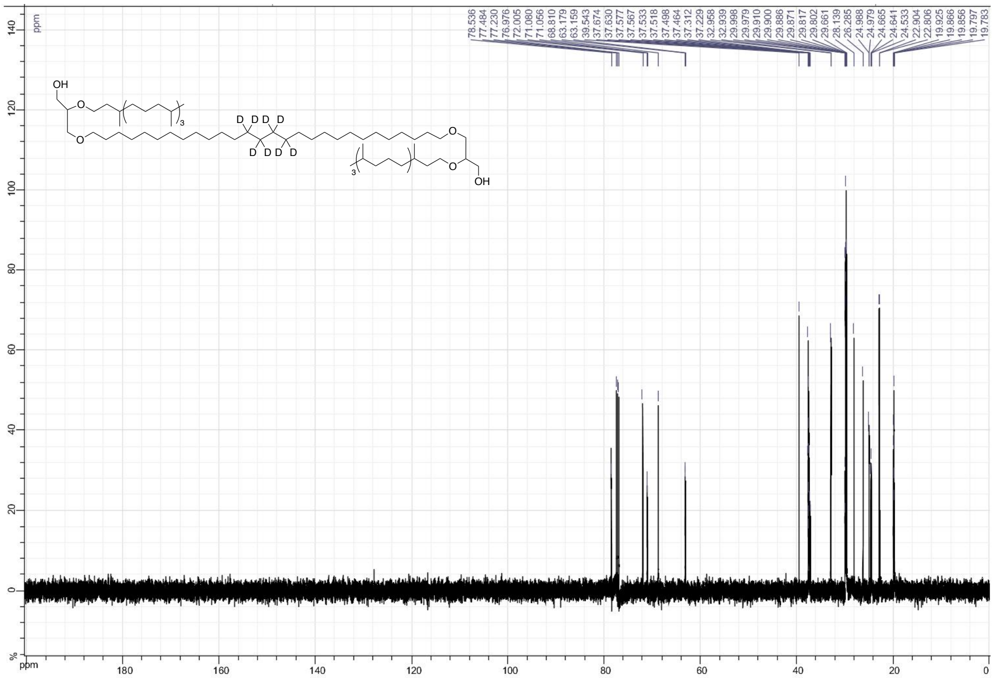

Figure S34. ^{13}C NMR Spectrum of compound **11**

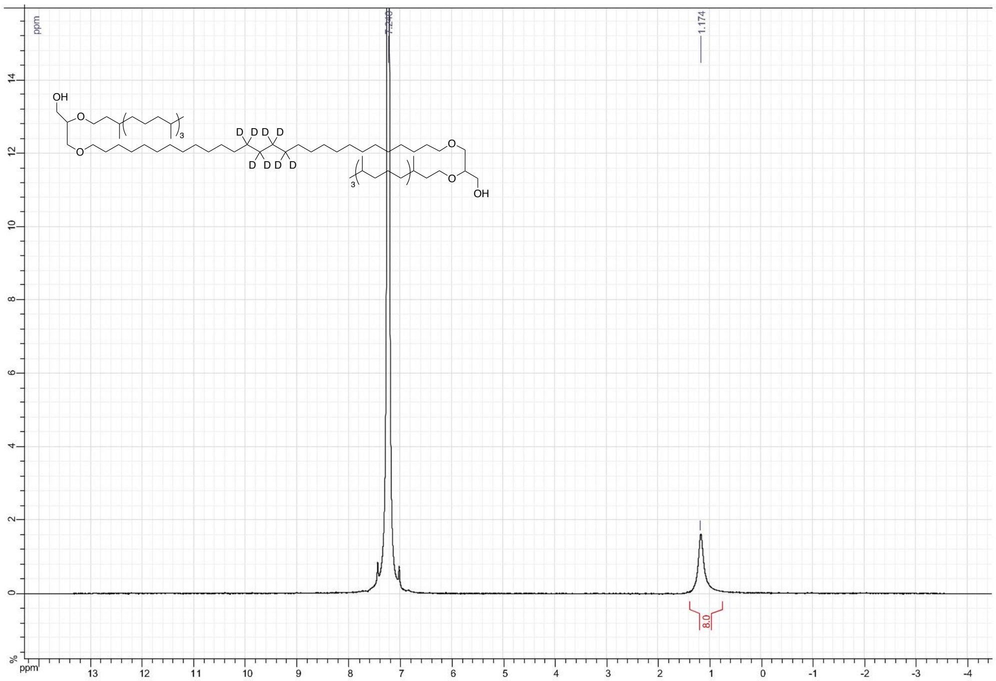

Figure S35. ^{2}H NMR Spectrum of compound **11**

Compound **12**

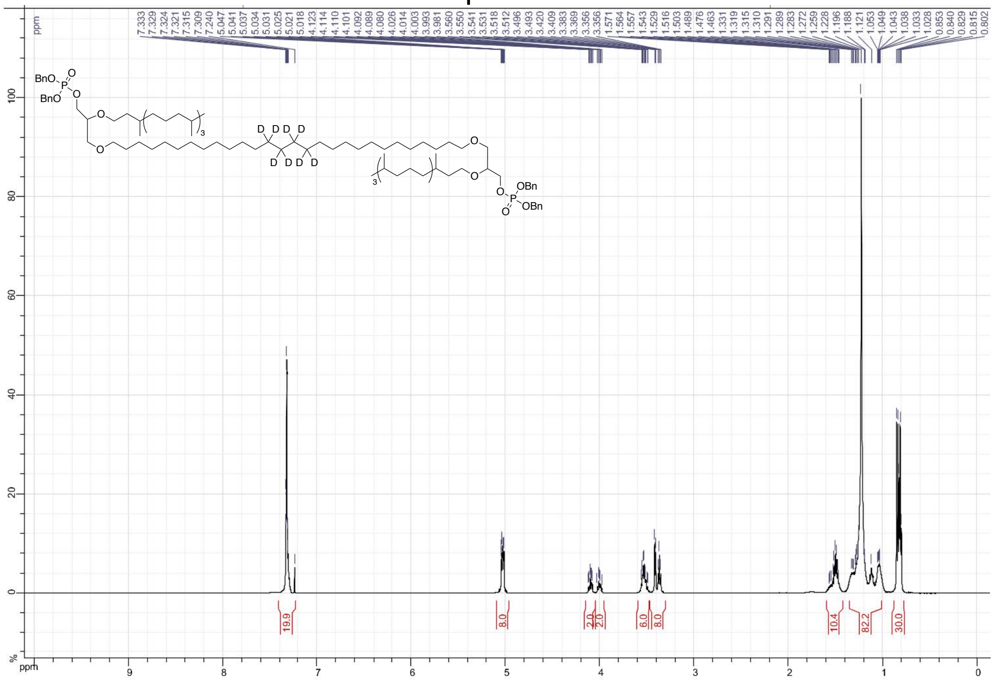

Figure S36. ^{1}H NMR Spectrum of compound **12**

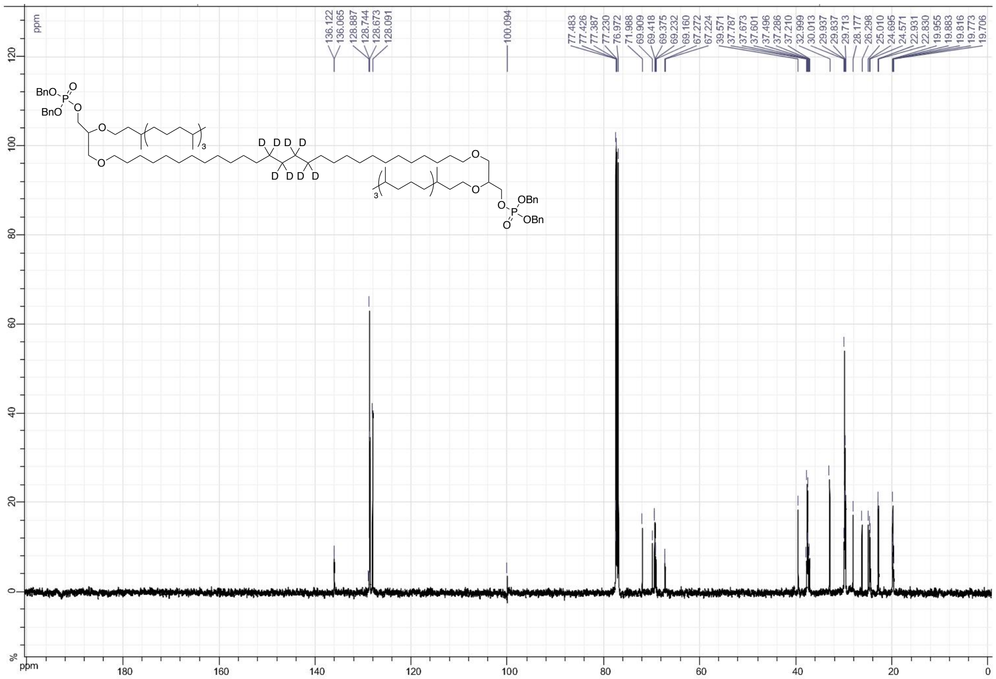

Figure S37. ^{13}C NMR Spectrum of compound **12**

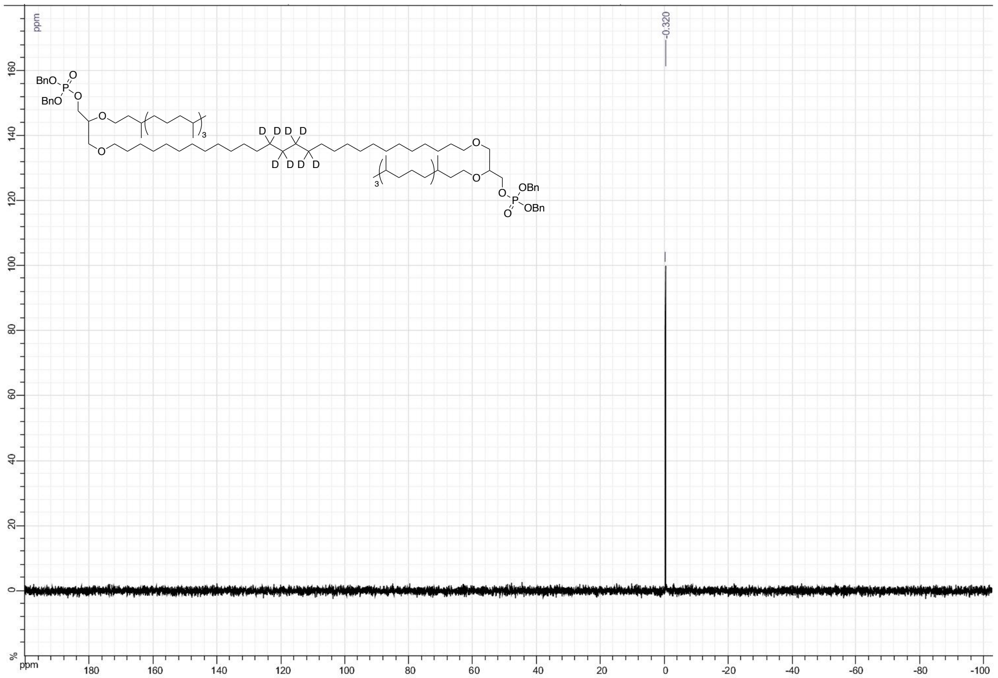

Figure S38. ^{31}P NMR Spectrum of compound **12**

D-GMGTPA

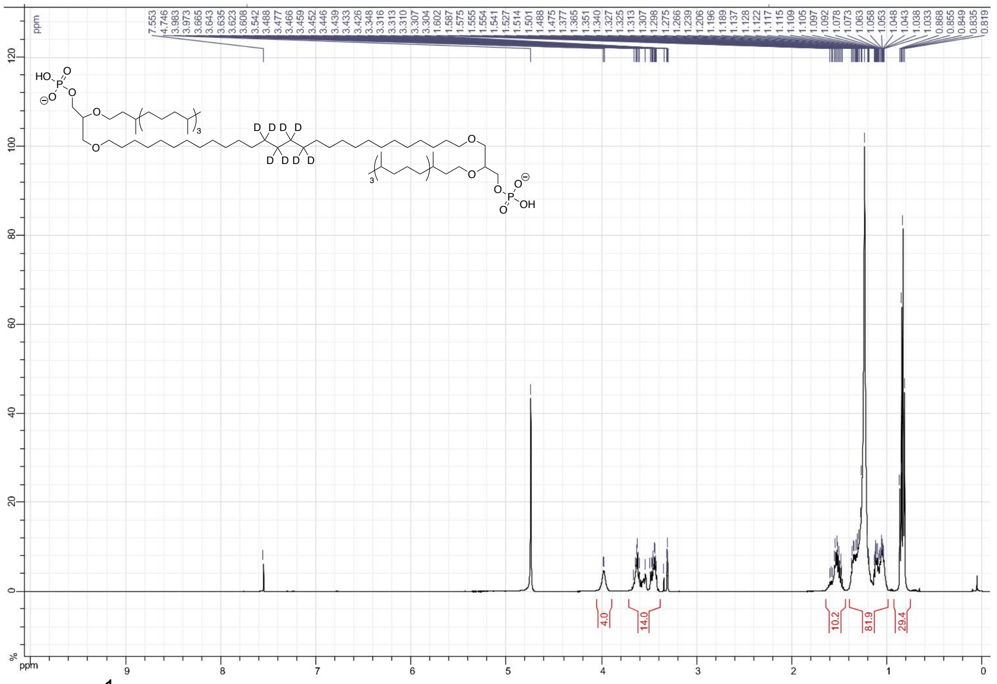

Figure S39. ^{1}H NMR Spectrum of compound **D-GMGTPA**

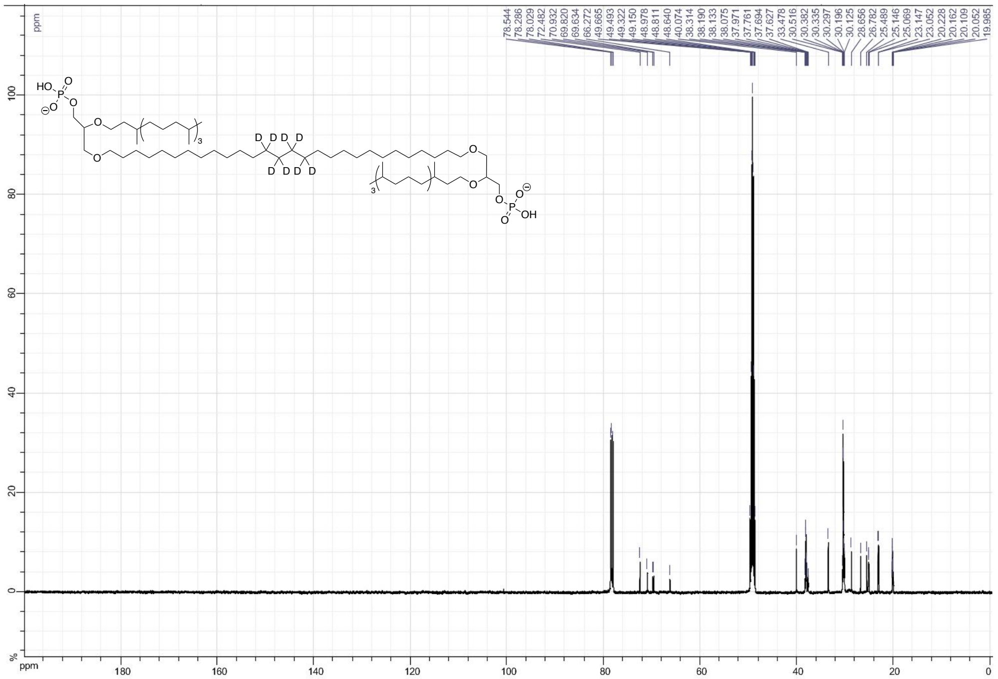

Figure S40. ^{13}C NMR Spectrum of compound **D-GMGTPA**

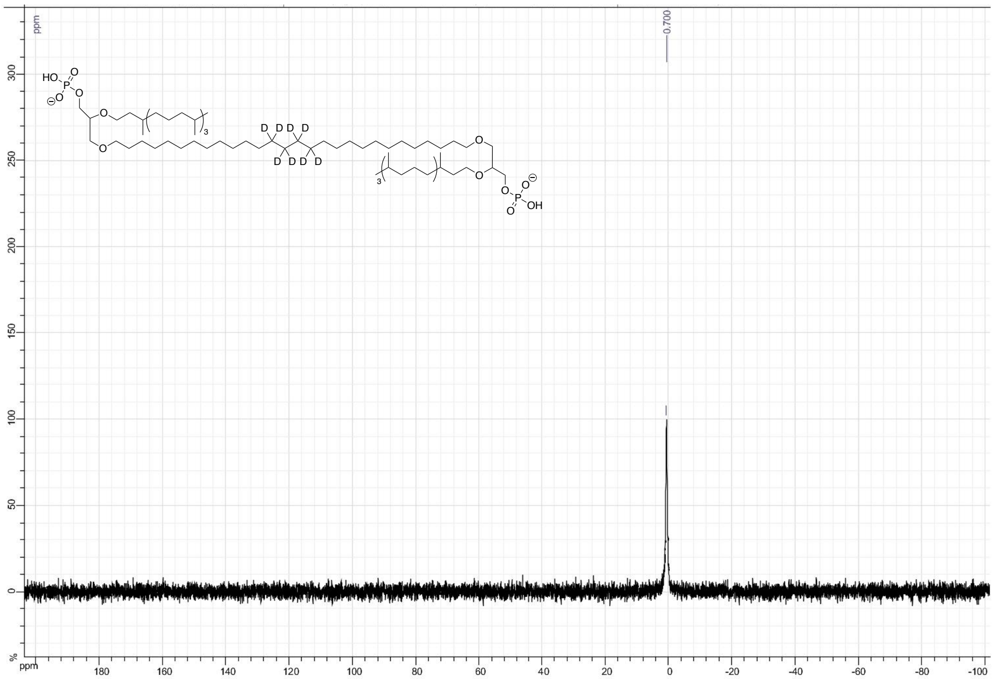

Figure S41. ^{31}P NMR Spectrum of compound **D-GMGTPA**

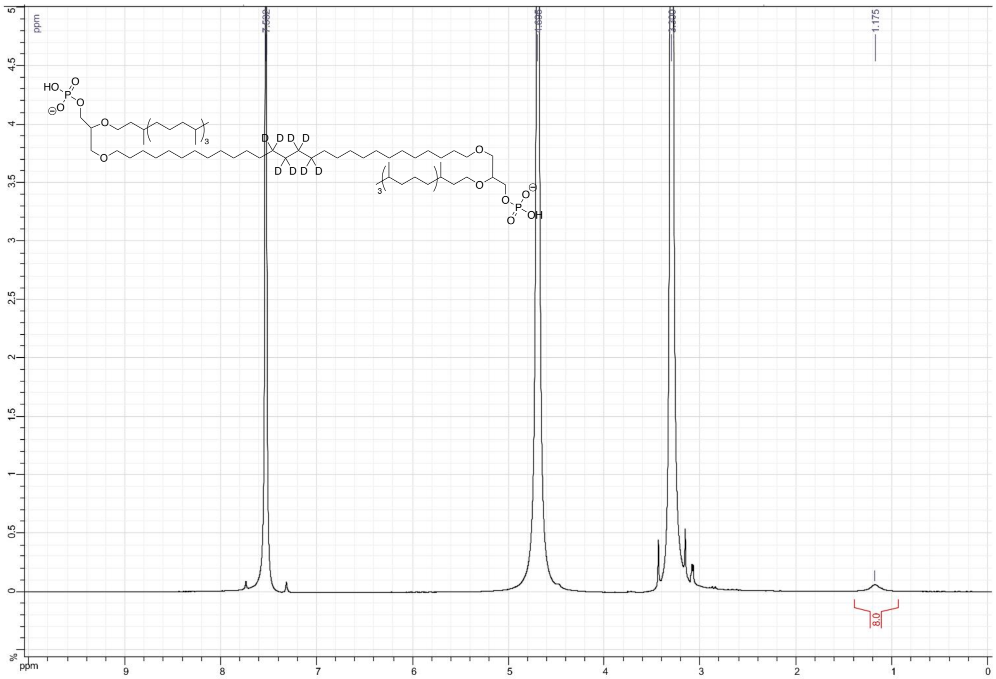

Figure S42. ^{2}H NMR Spectrum of compound **D-GMGTPA**

Compound **S3**

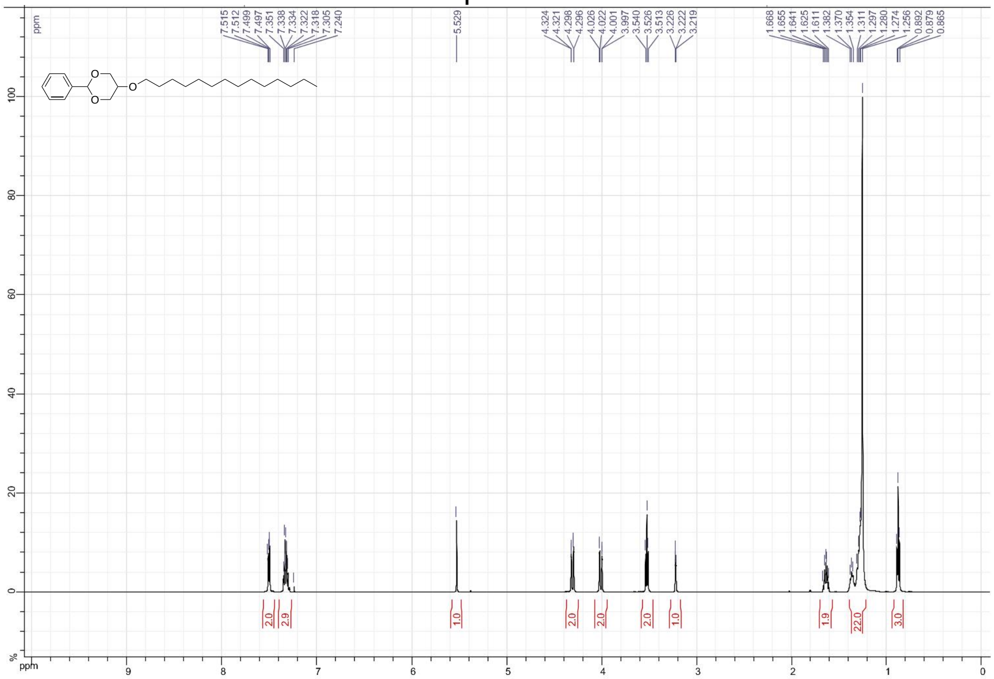

Figure S43. ^{1}H NMR Spectrum of compound **S3**

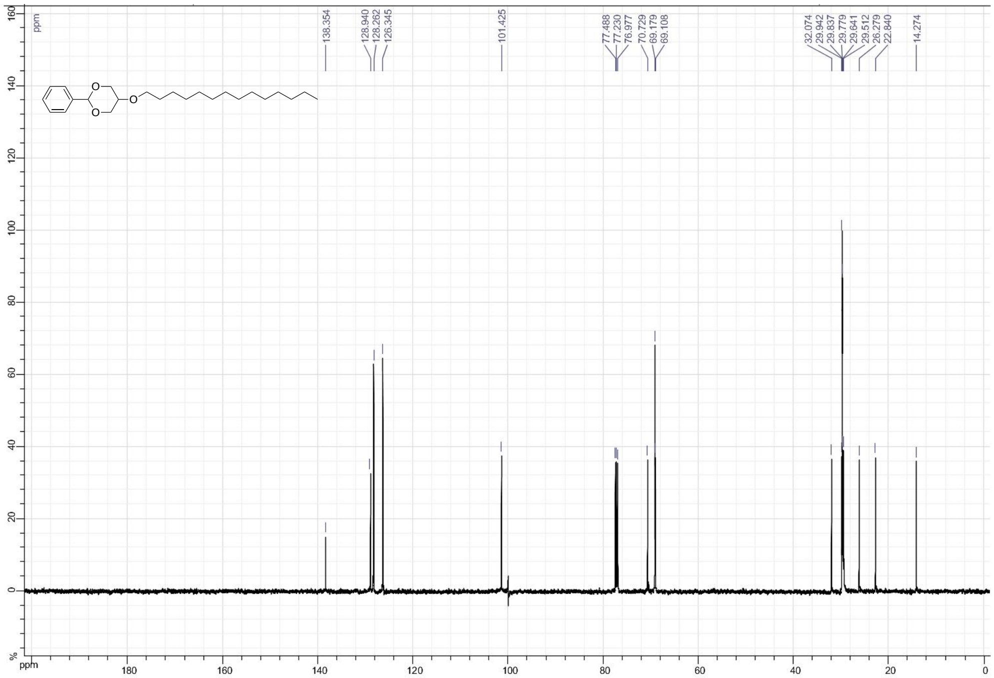

Figure S44. ^{13}C NMR Spectrum of compound **S3**

Compound **S4**

^{1}H NMR Spectrum

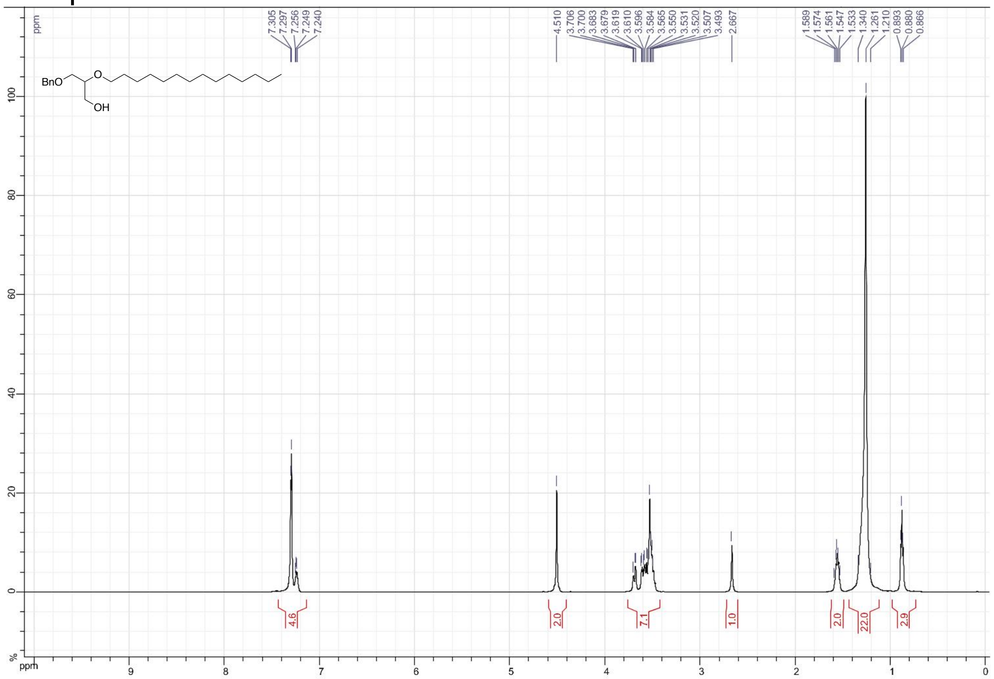

Figure S45. ^{1}H NMR Spectrum of compound **S4**

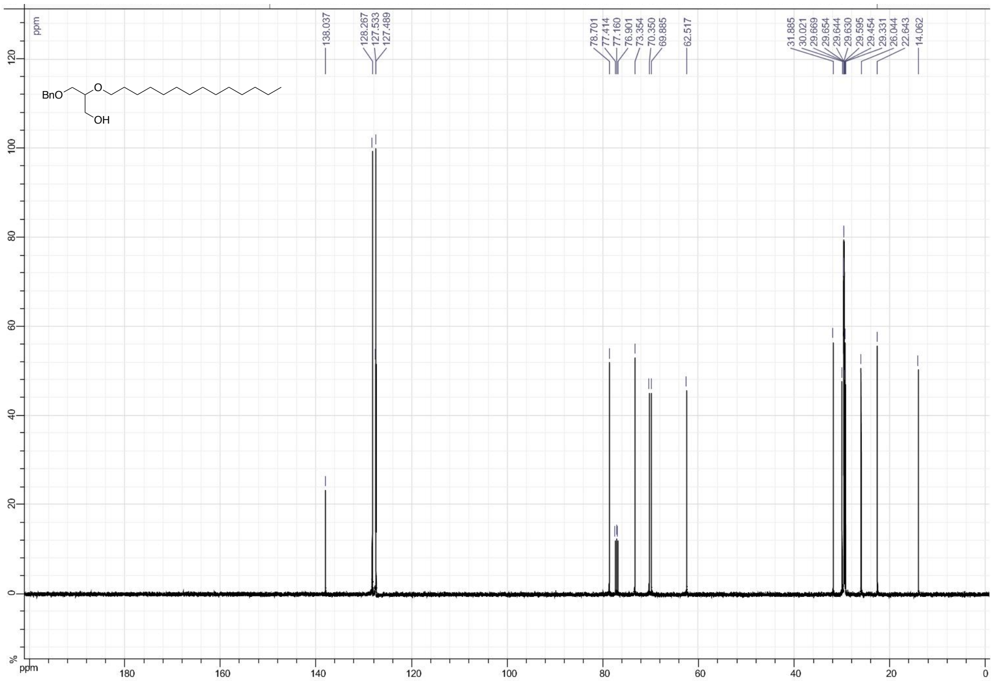

Figure S46. ^{13}C NMR Spectrum of compound **S4**

Compound **S5**

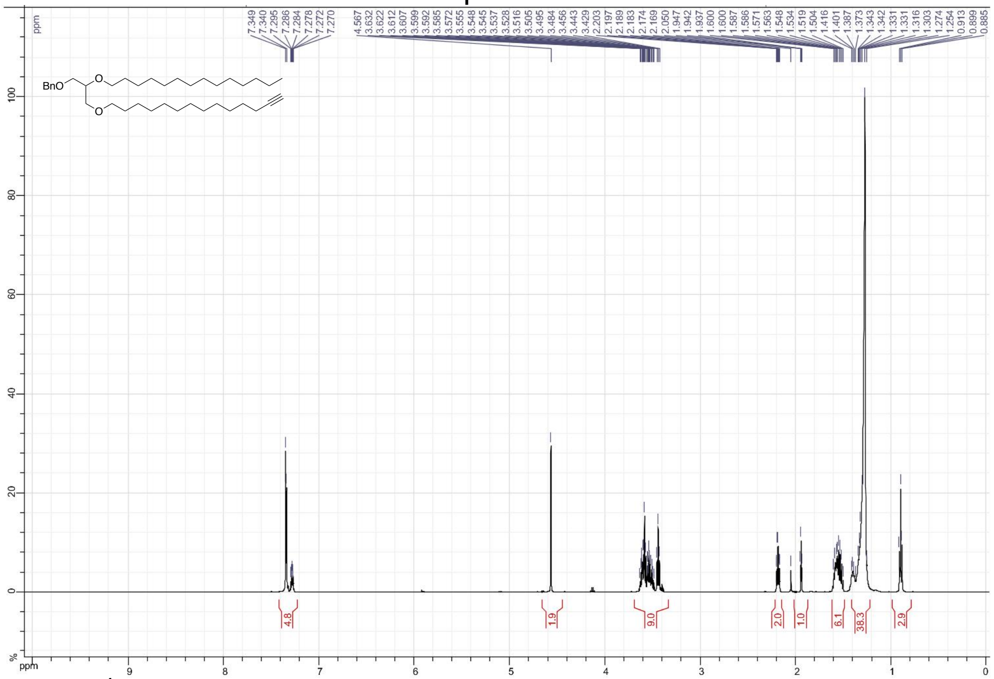

Figure S47. ^{1}H NMR Spectrum of compound **S5**

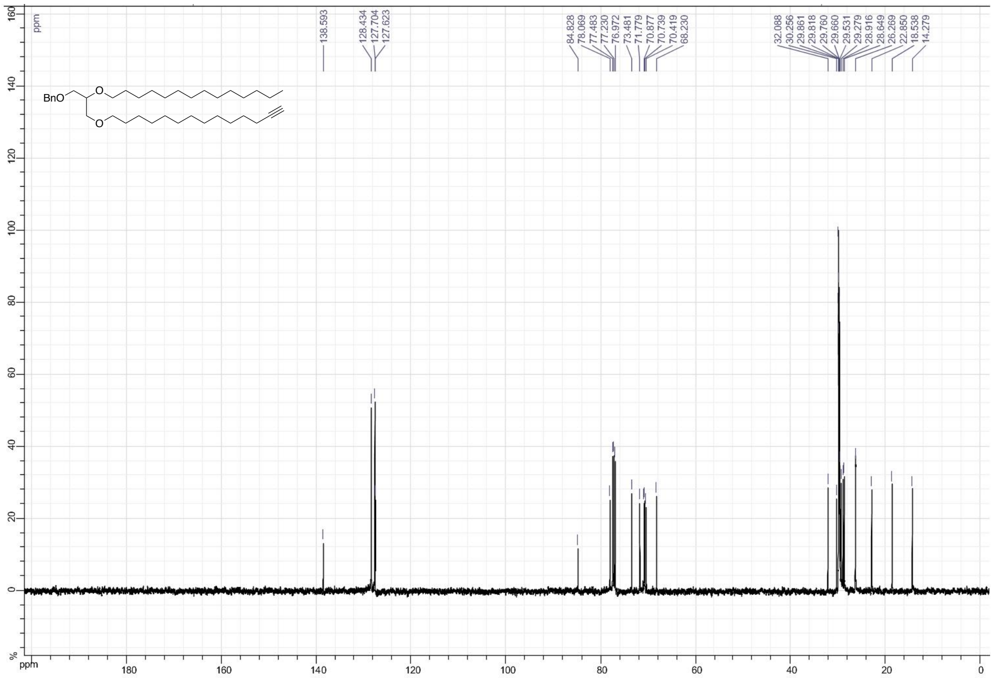

Figure S48. ^{13}C NMR Spectrum of compound **S5**

Compound **S6**

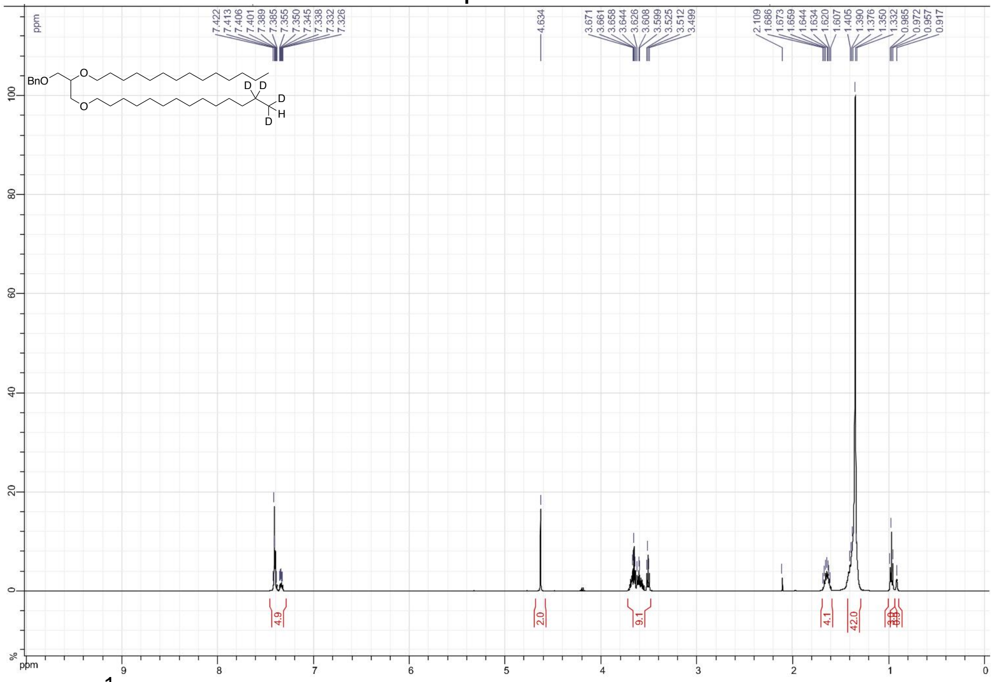

Figure S49. ^{1}H NMR Spectrum of compound **S6**

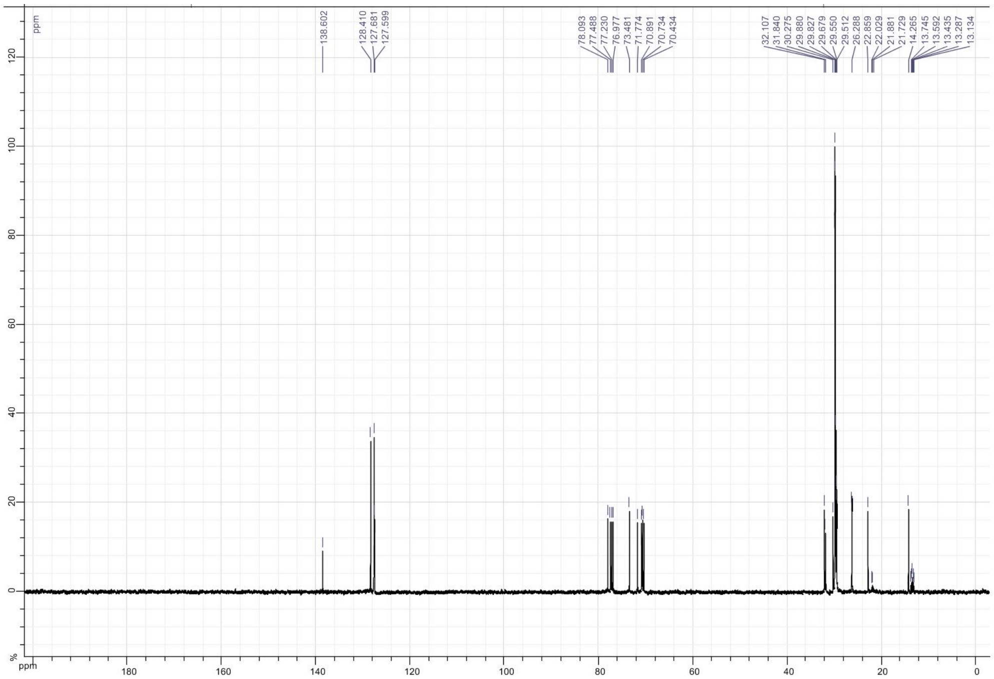

Figure S50. ^{13}C NMR Spectrum of compound **S6**

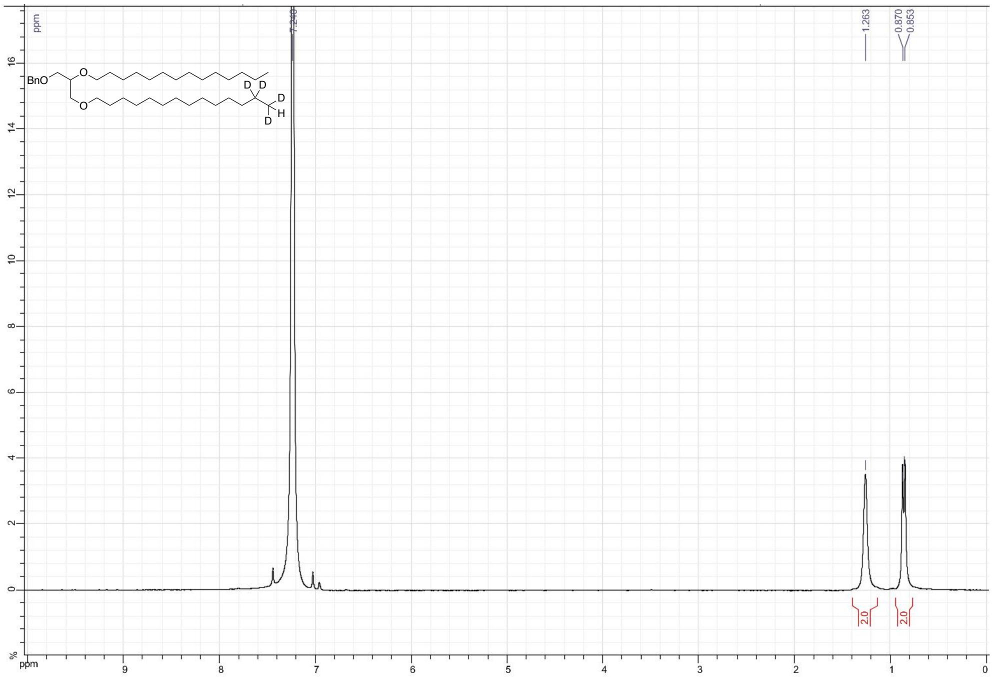

Figure S51. ^{2}H NMR Spectrum of compound **S6**

Compound **S7**

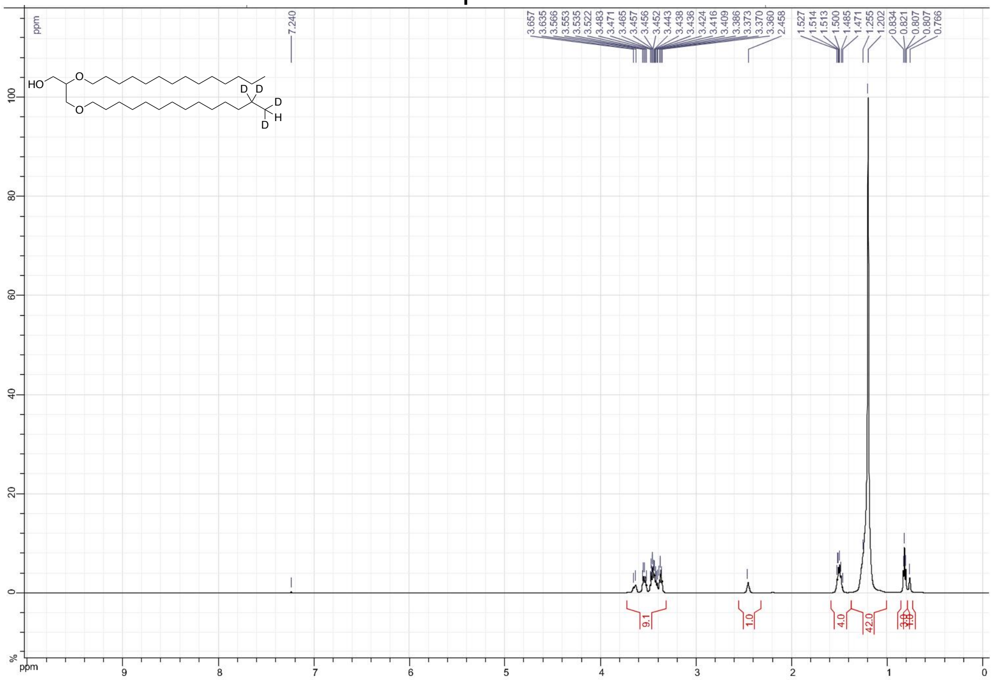

Figure S52. ^{1}H NMR Spectrum of compound **S7**

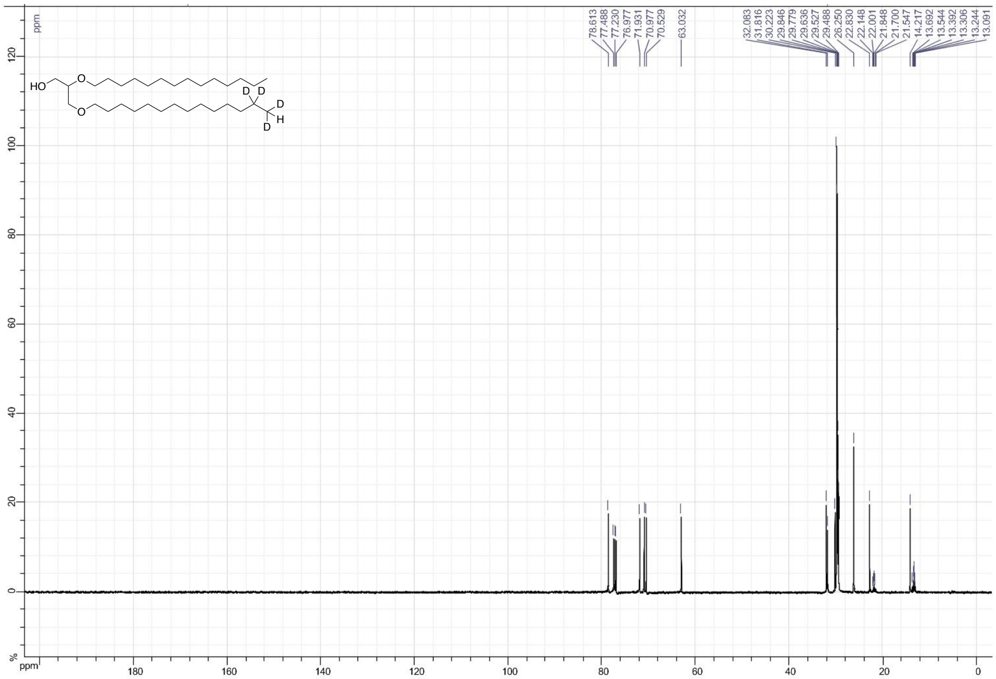

Figure S53. ^{13}C NMR Spectrum of compound **S7**

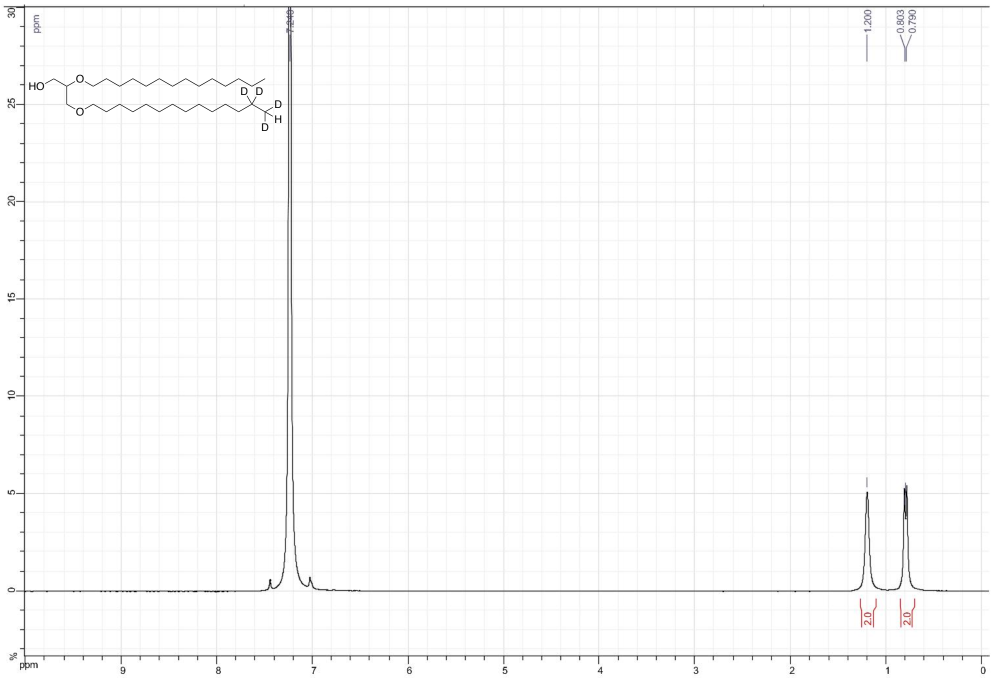

Figure S54. ^{2}H NMR Spectrum of compound **S7**

Compound **S8**

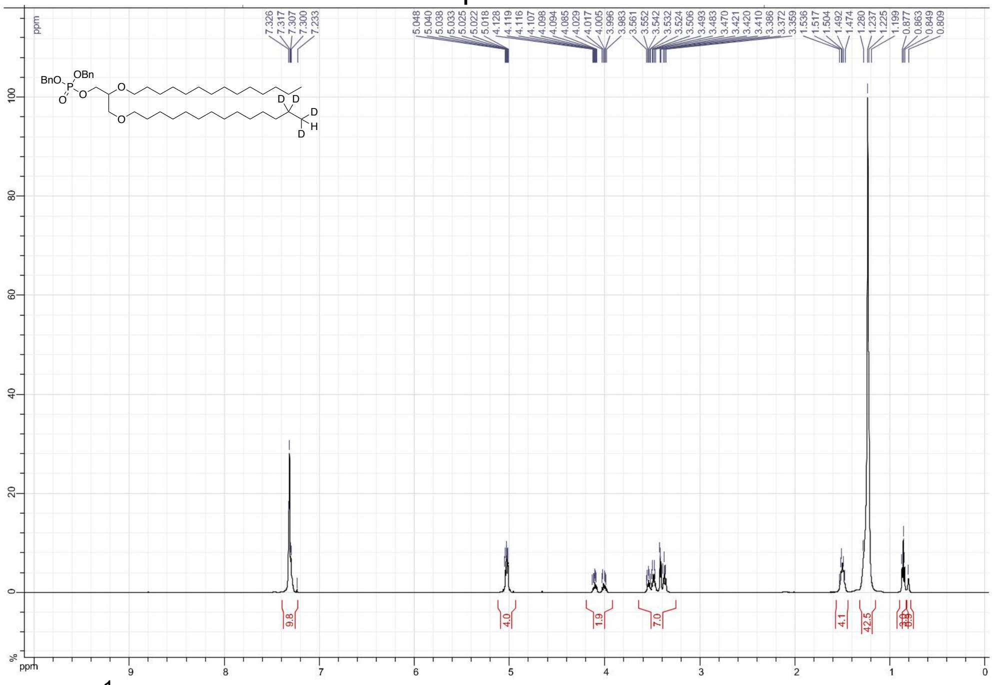

Figure S55. ^{1}H NMR Spectrum of compound **S8**

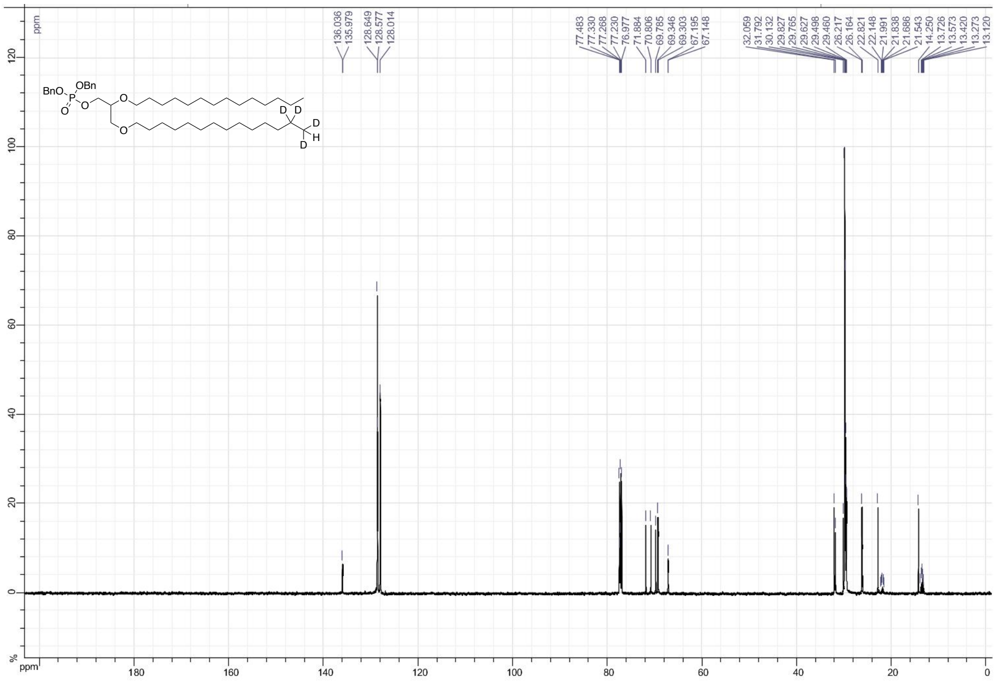

Figure S56. ^{13}C NMR Spectrum of compound **S8**

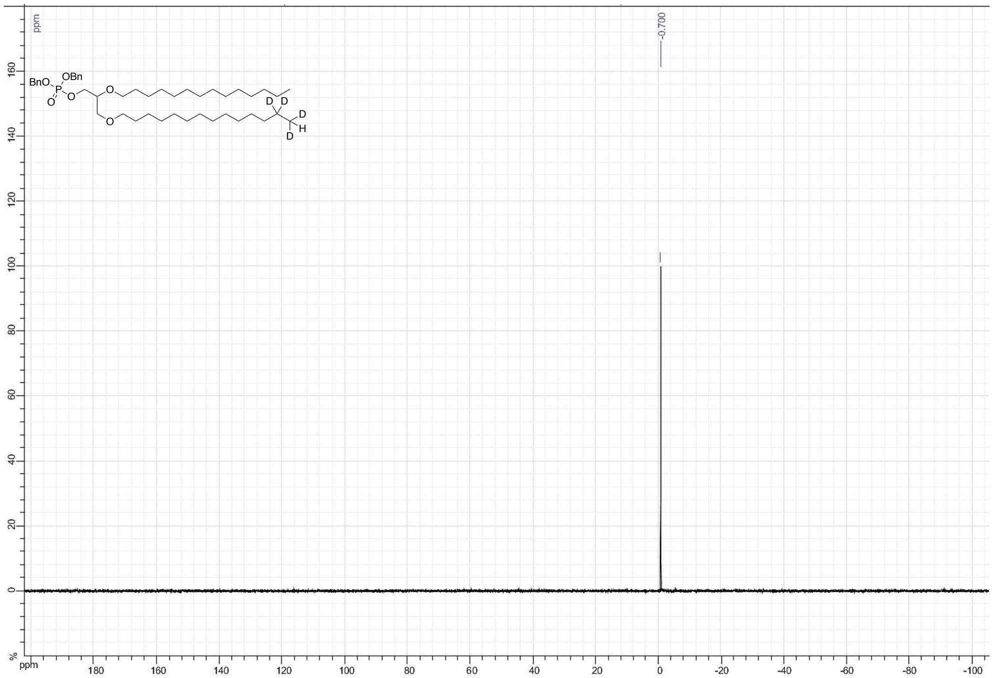

Figure S57. ^{31}P NMR Spectrum of compound **S8**

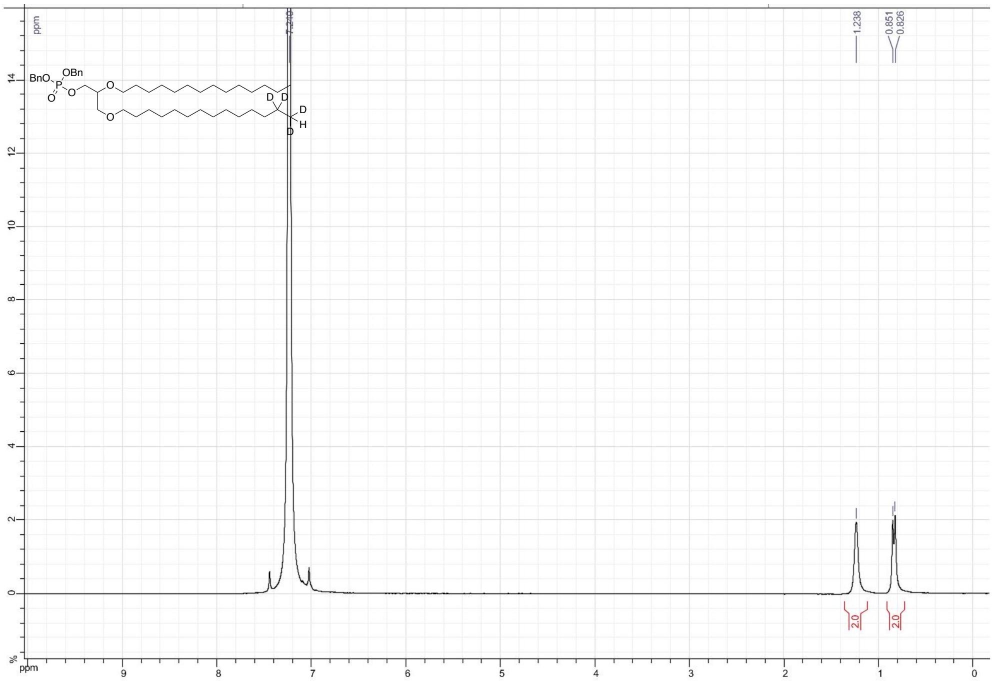

Figure S58. ^{2}H NMR Spectrum of compound **S8**

Compound **13**

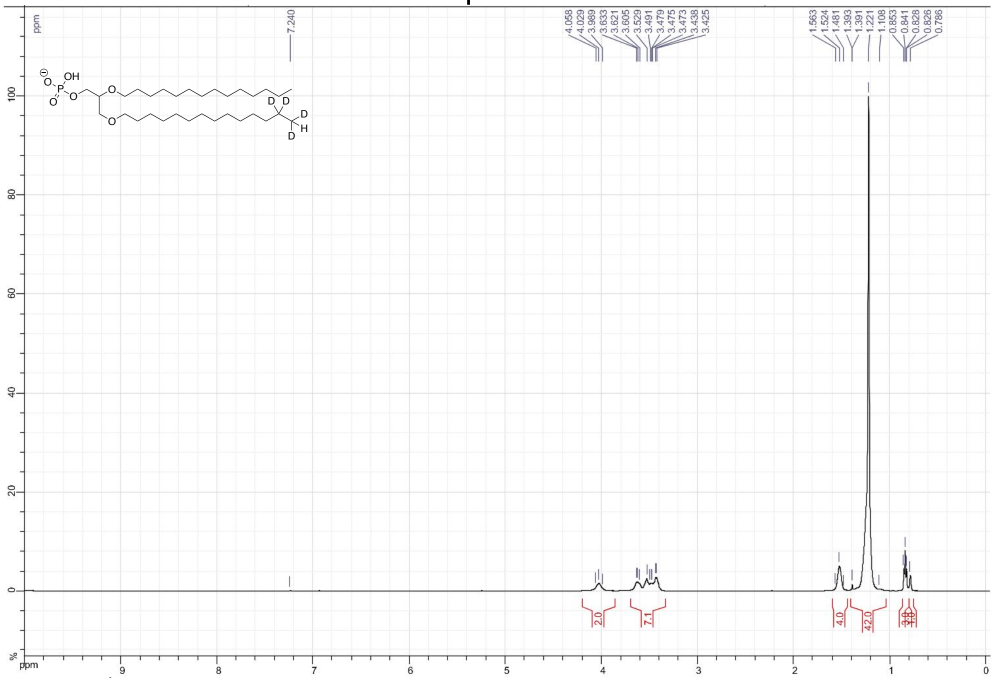

Figure S59. ^{1}H NMR Spectrum of compound **13**

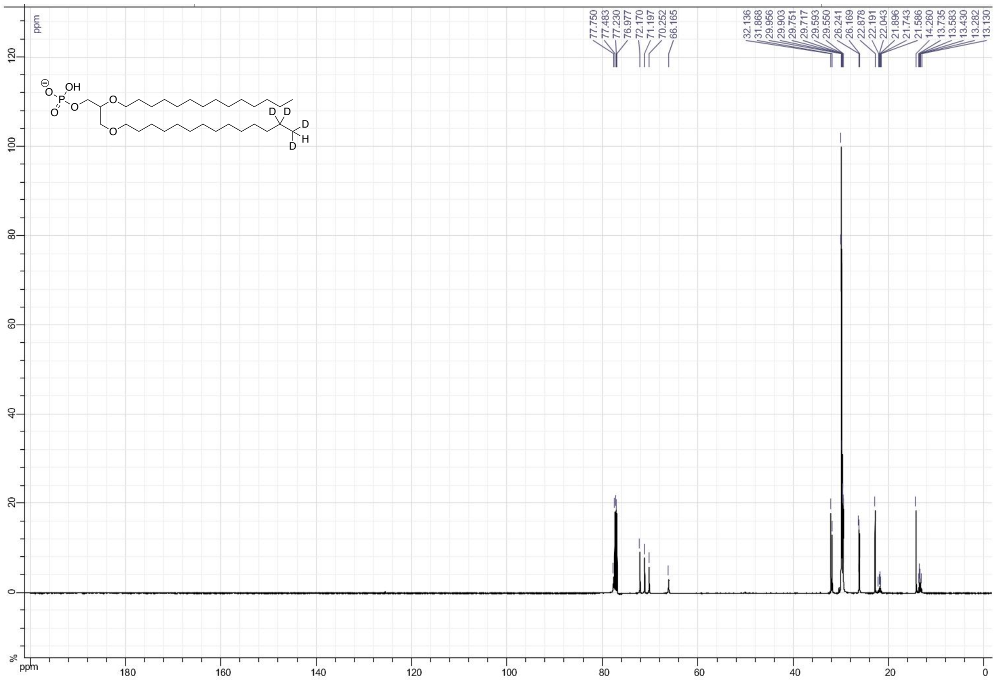

Figure S60. ^{13}C NMR Spectrum of compound **13**

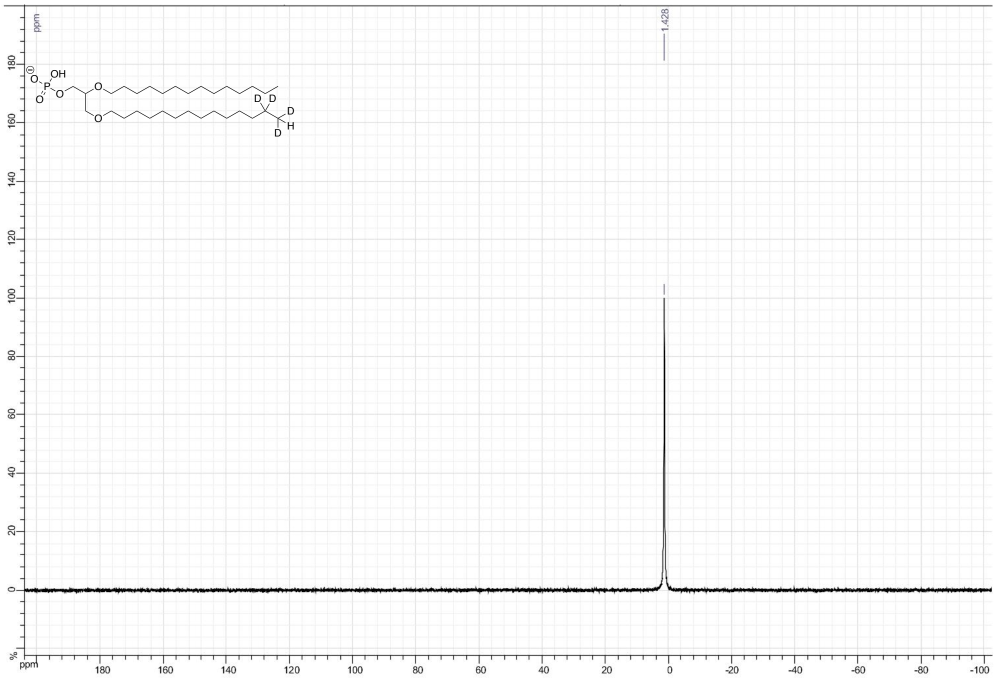

Figure S61. ^{31}P NMR Spectrum of compound **13**

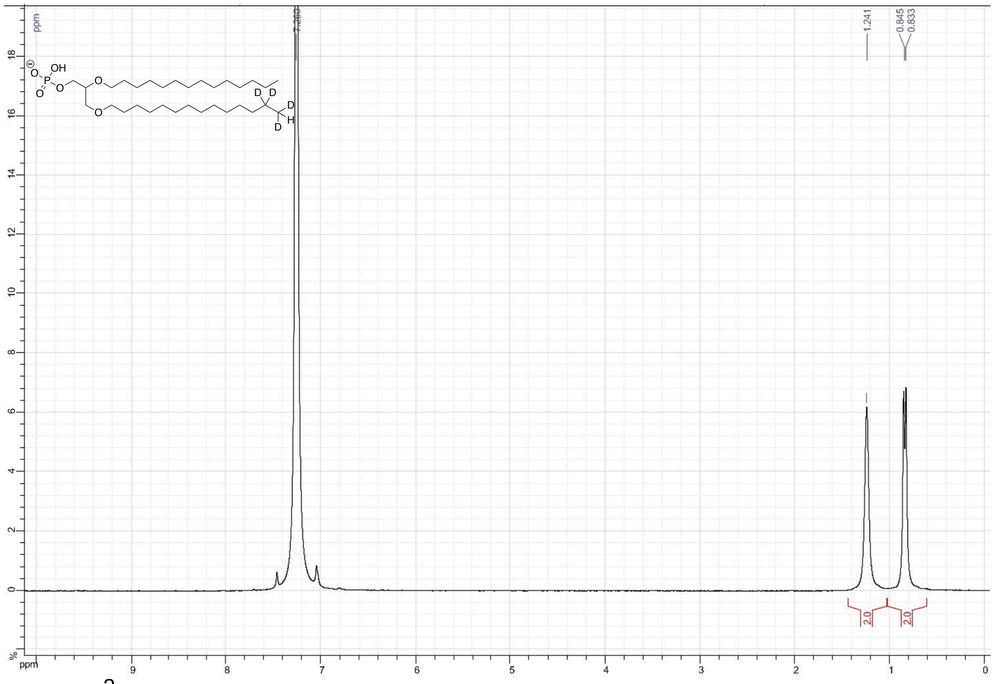

Figure S62. ^{2}H NMR Spectrum of compound **13**

Compound **S10**

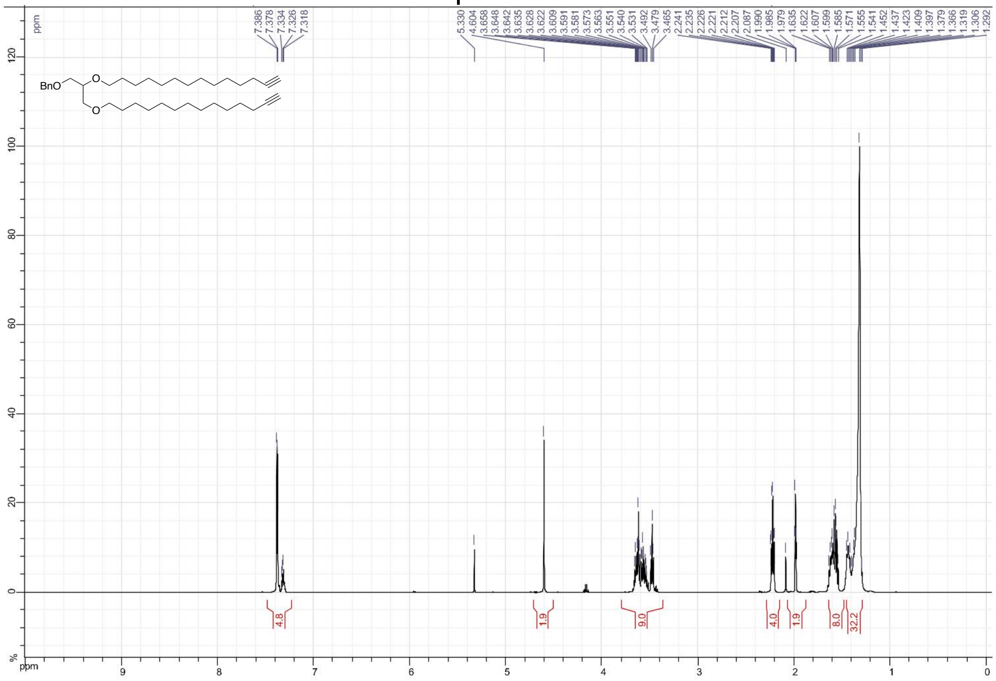

Figure S63. ^{1}H NMR Spectrum of compound **S10**

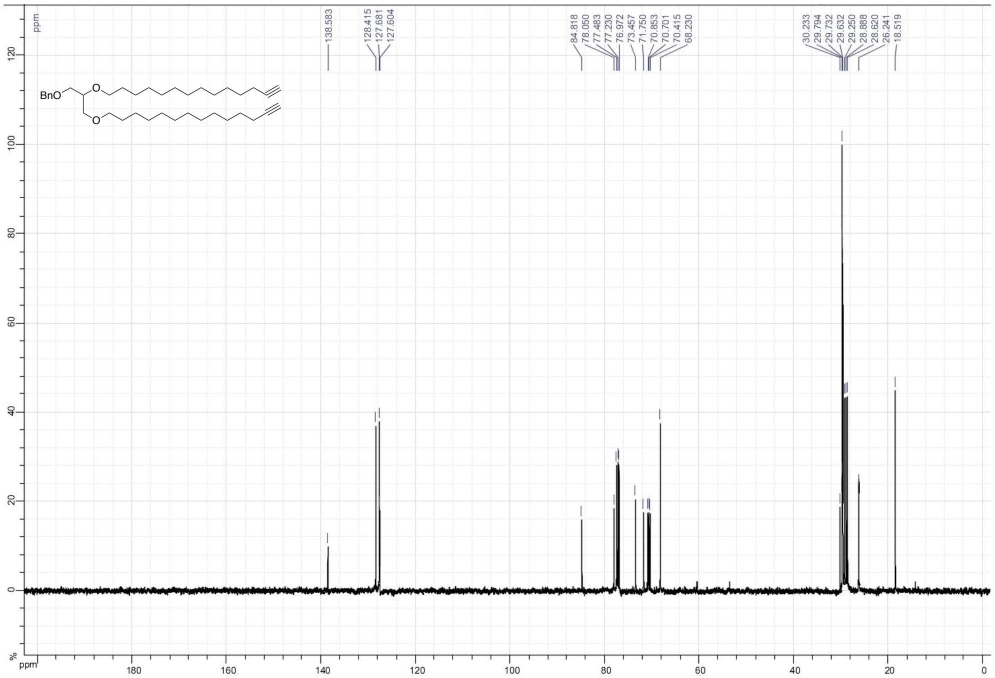

Figure S64. ^{13}C NMR Spectrum of compound **S10**

Compound **S11**

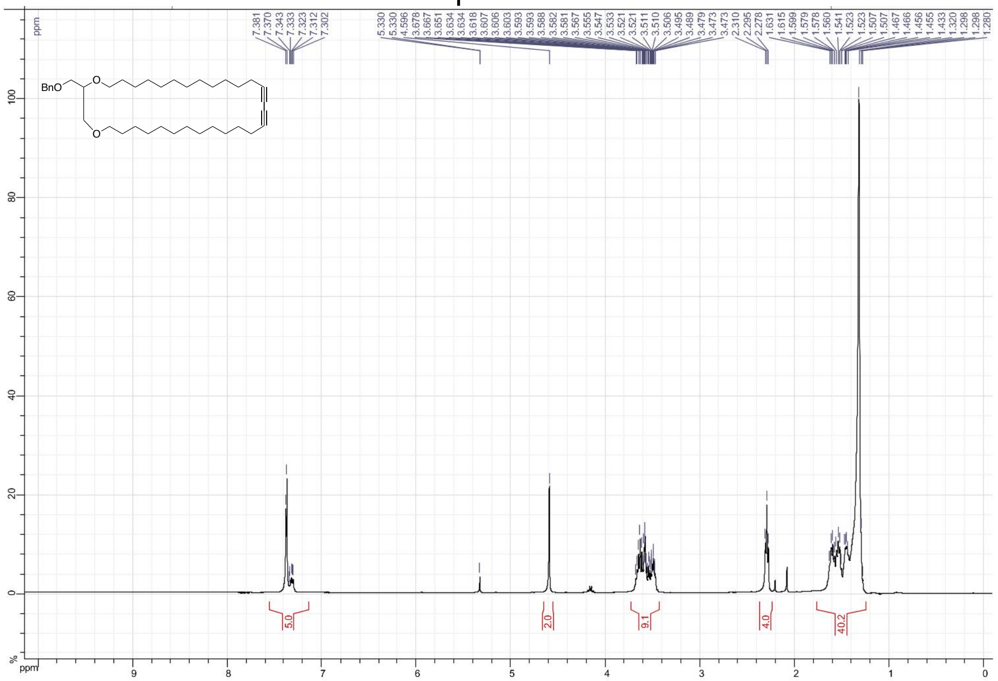

Figure S65. ^{1}H NMR Spectrum of compound **S11**

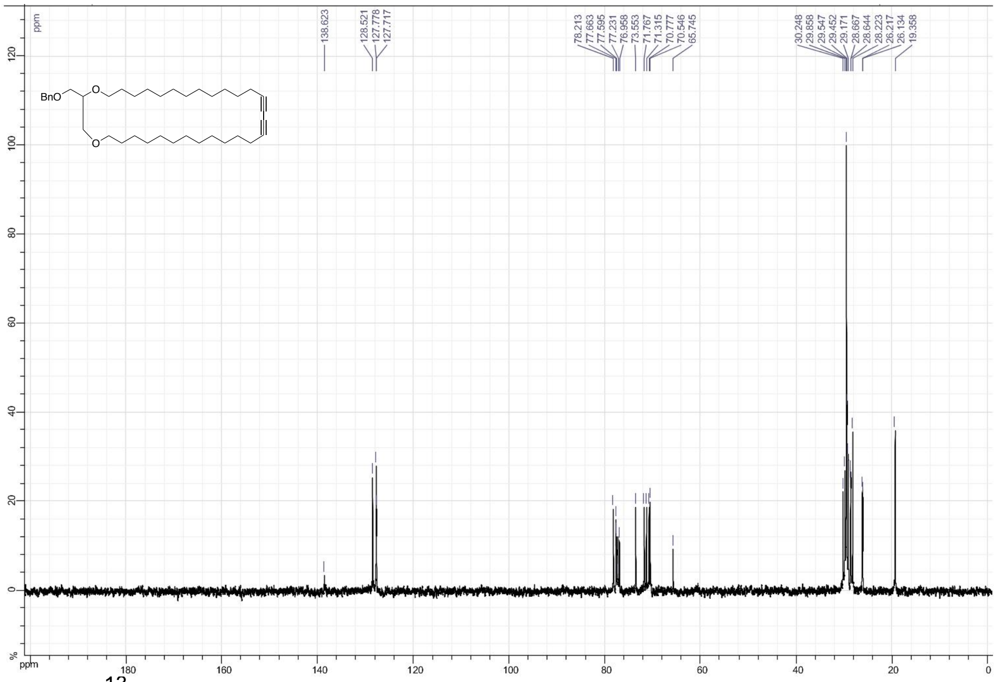

Figure S66. ^{13}C NMR Spectrum of compound **S11**

Compound **S12**

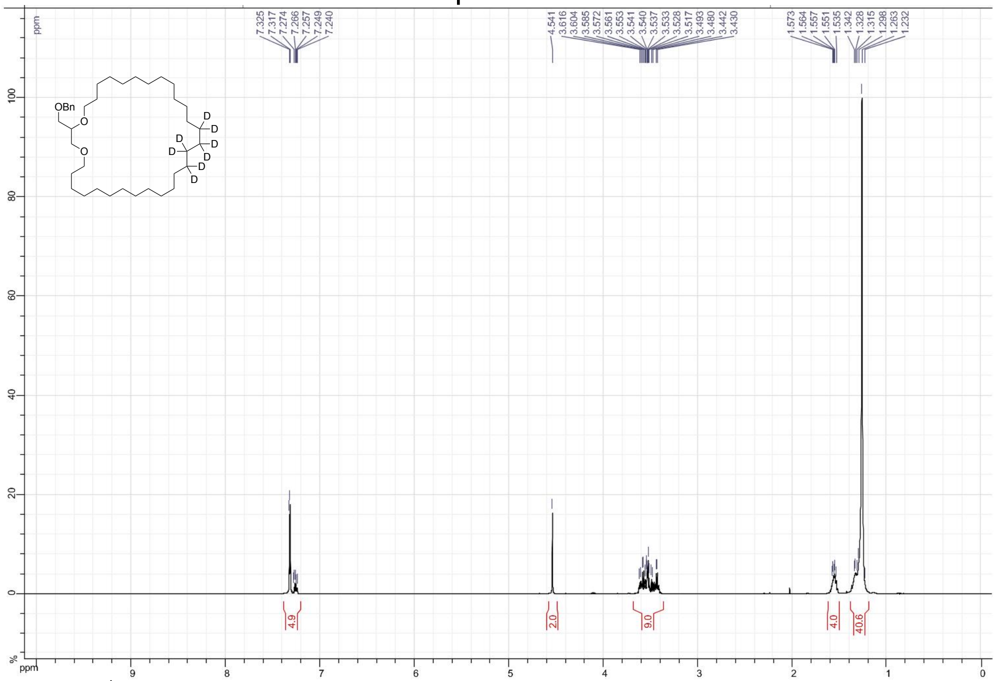

Figure S67. ^{1}H NMR Spectrum of compound **S12**

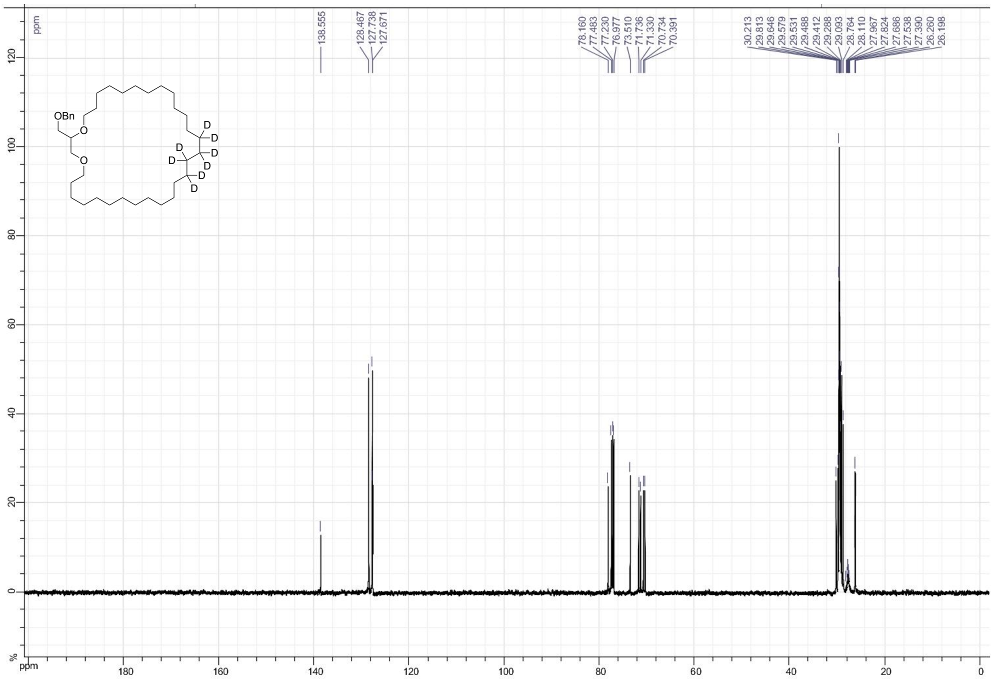

Figure S68. ^{13}C NMR Spectrum of compound **S12**

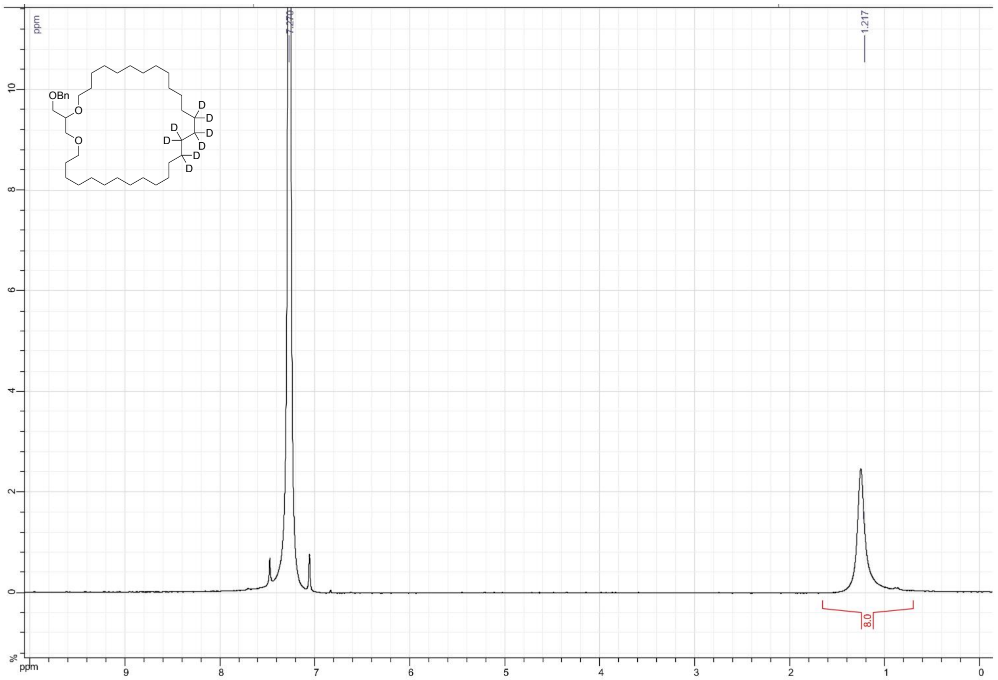

Figure S69. ^{2}H NMR Spectrum of compound **S12**

Compound **S14**

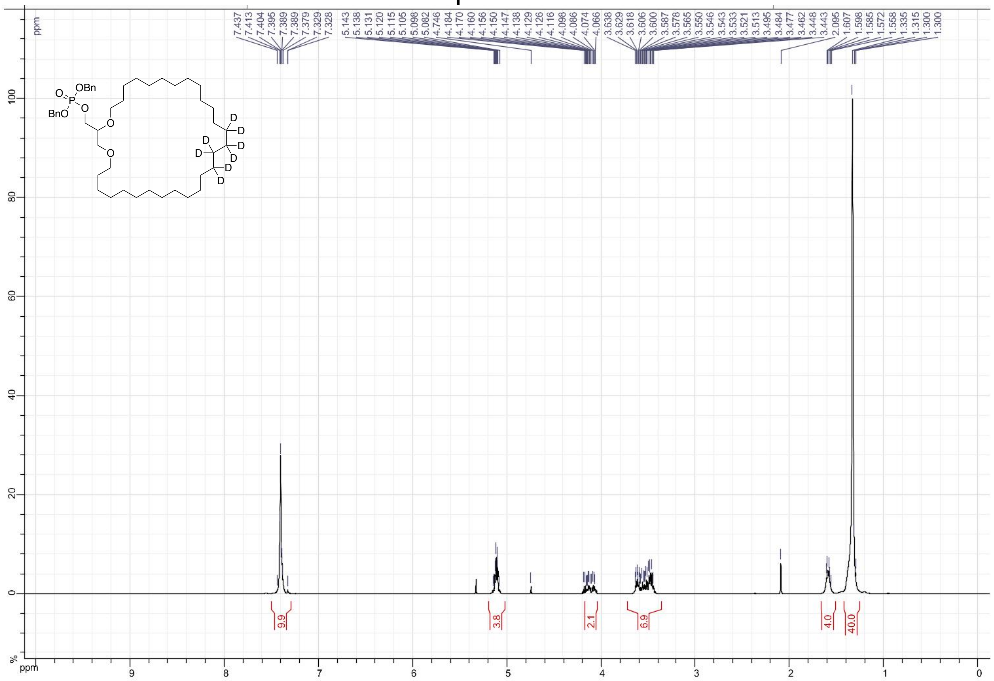

Figure S70. ^{1}H NMR Spectrum of compound **S14**

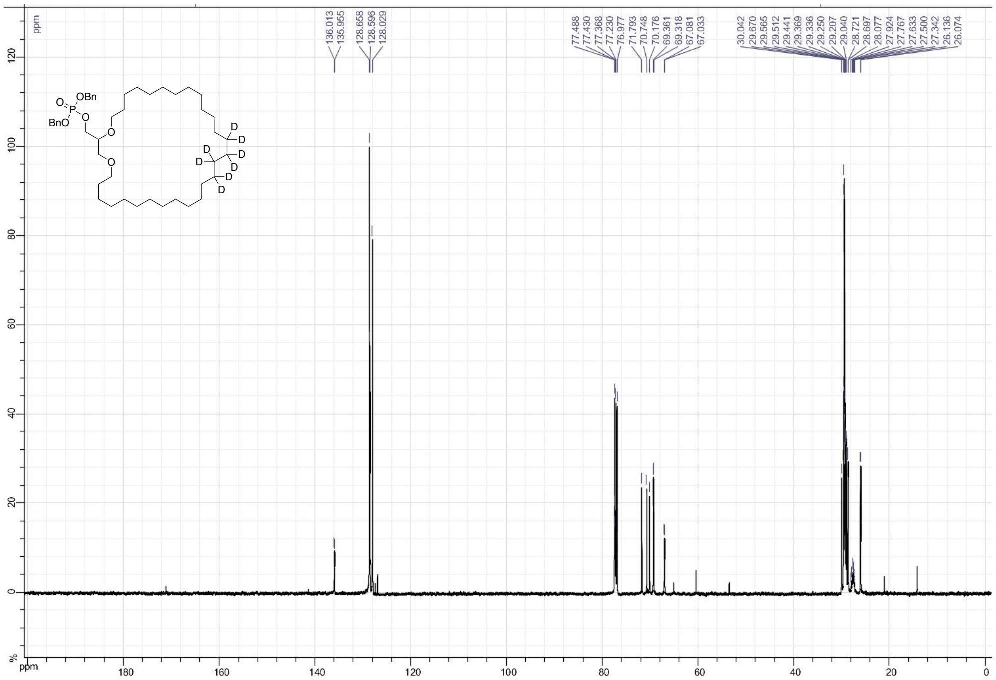

Figure S71. ^{13}C NMR Spectrum of compound **S14**

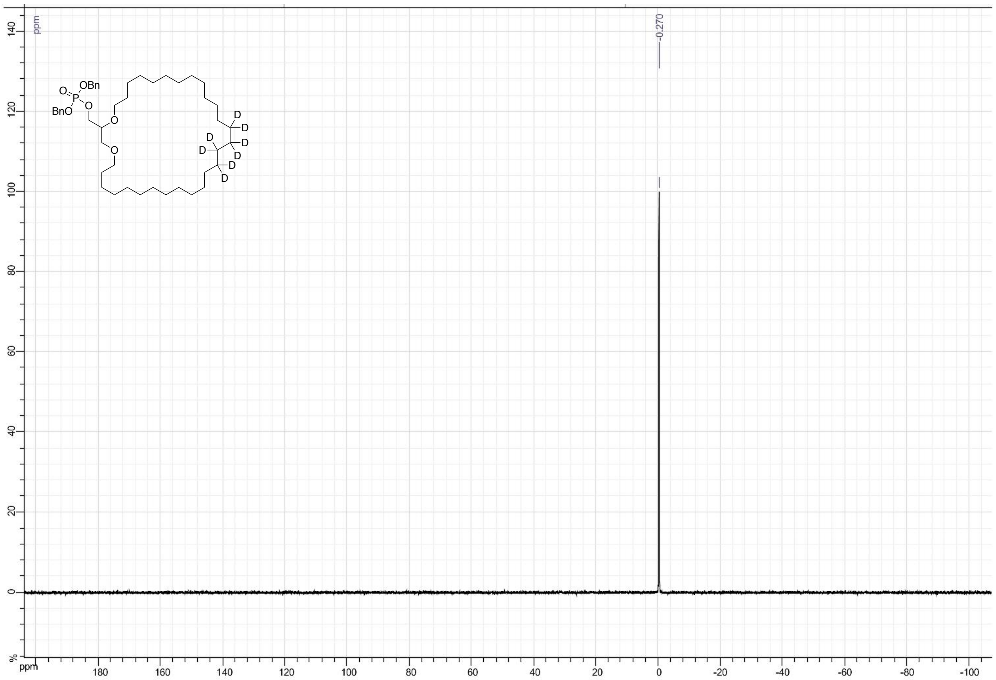

Figure S72. ^{31}P NMR Spectrum of compound **S14**

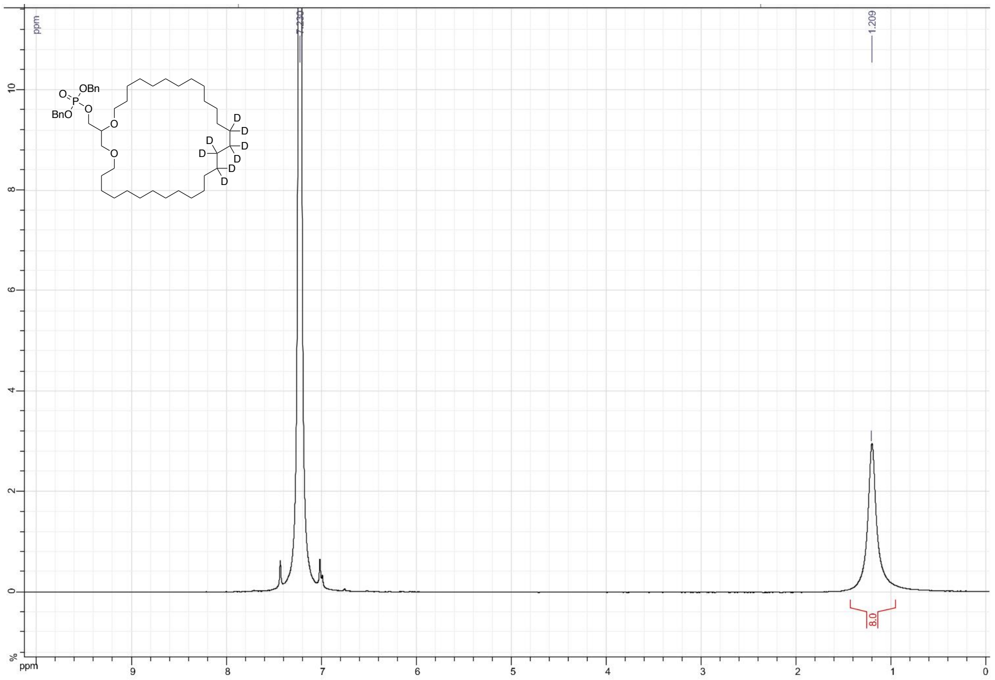

Figure S73. ^{2}H NMR Spectrum of compound **S14**

Compound **14**

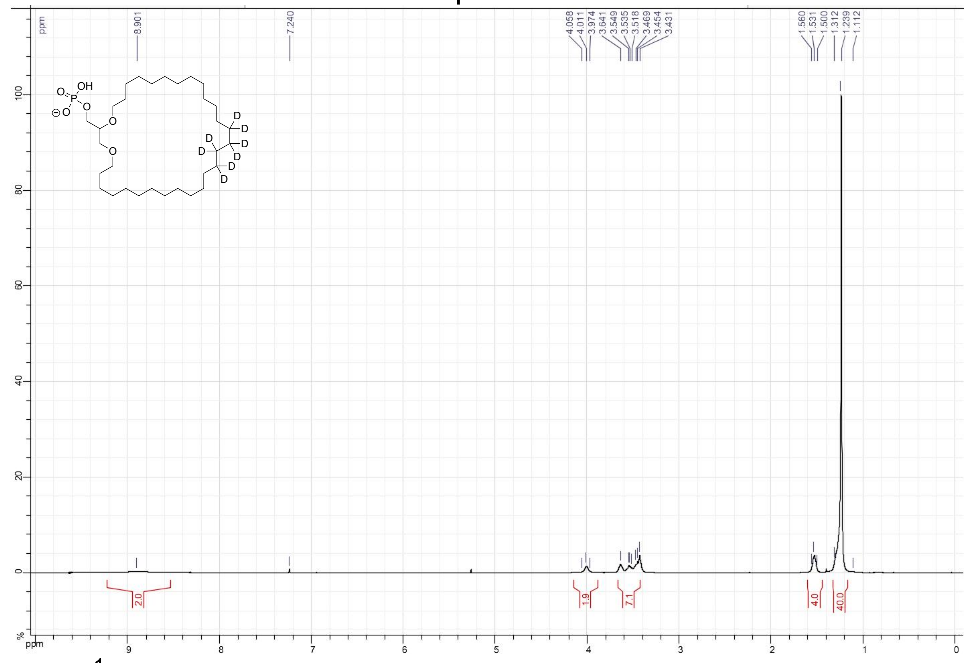

Figure S74. ^{1}H NMR Spectrum of compound **14**

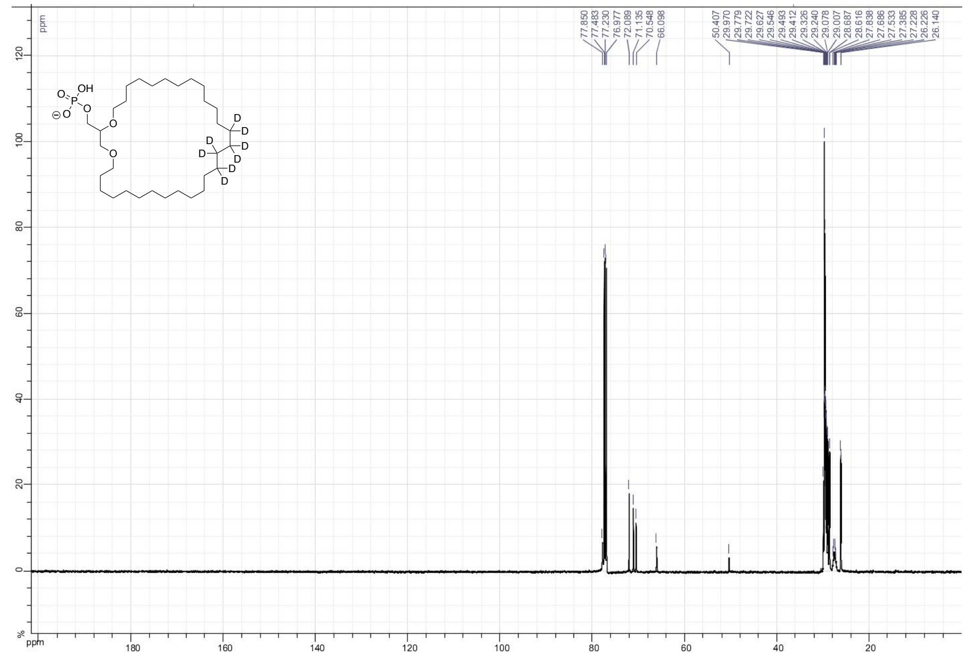

Figure S75. ^{13}C NMR Spectrum of compound **14**

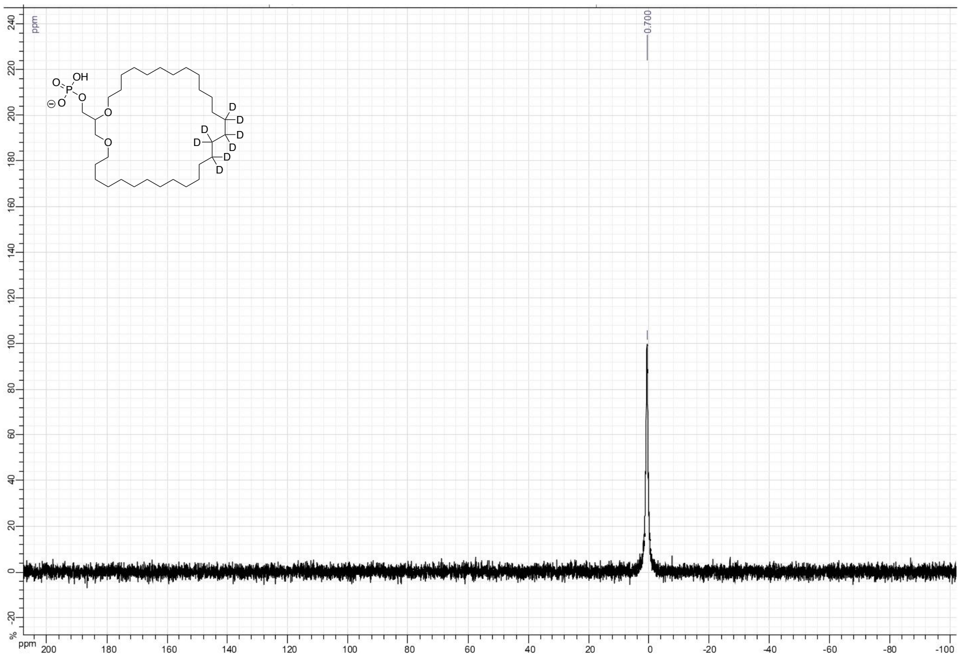

Figure S76. ^{31}P NMR Spectrum of compound **14**

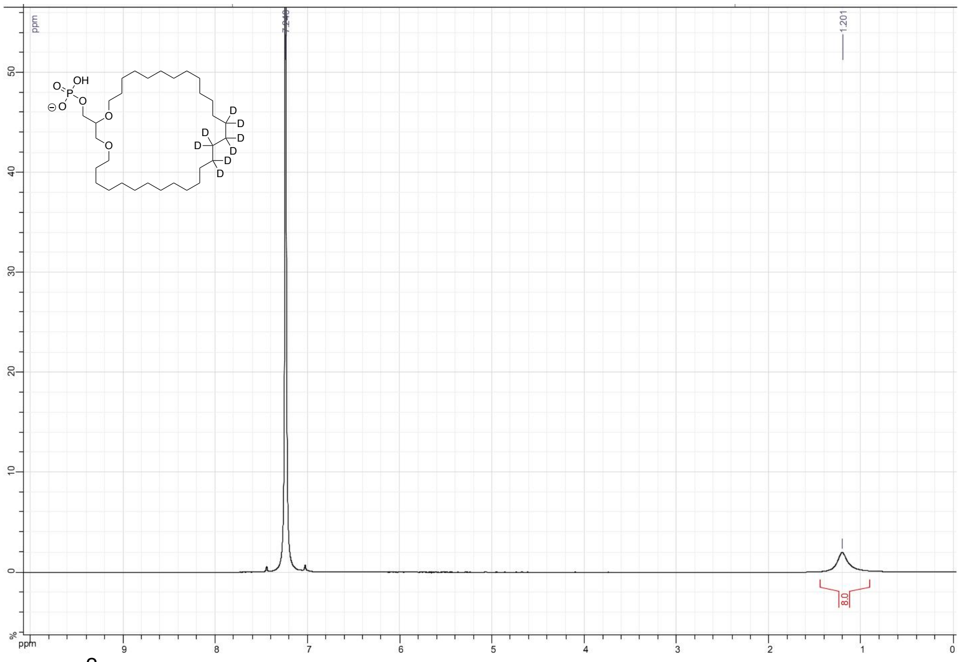

Figure S77. ^{2}H NMR Spectrum of compound **14**
